# Supplementary material for: Six domesticated PiggyBac transposases together carry out programmed DNA elimination in Paramecium
Source: eLife. 2018 Sep 18;7:e37927. doi: 10.7554/eLife.37927 (PMC6143343; doi:10.7554/eLife.37927)
Supplement: Supplementary file 1. [file elife-37927-supp1.pdf]

```
PgmL2b-Psex_PSE    1 ---------------------------QGERNAYNFWKSQVTNDAK-DIYYS-LFHRQ--
PgmL2a-Psex_PSE    1 ---------------------------QGERNAYNFWKSQVTNDAK-DIYYS-LFHRQ--
PgmL2-Ptet_PTET    1 ---------------------------QGDRNAYNFWKSQVTNDAK-DIYYS-LFHRQ--
PgmL2-Pbi_PBIGN    1 ---------------------------QGERNAYNFWKSQVTNDAK-DIYYS-LFHRQ--
PgmL1-Pcau_PCAU    1 ----------------------------------EPWNLLQKESLV-QIYKS-LFAPK--
PgmL1-Psex_PSEX    1 -----------------------------------------NETPI-GIYNN-IFPKK--
PgmL1-Ptet_PTET    1 -----------------------------------------NETPM-GIYNS-IFPKK--
PgmL1-Pbi_PBIGN    1 -----------------------------------------NETPM-GIYNT-IFPKK--
Lia5_TTHERM_006    1 ---------------------------------GKSLQLTNINDSS-RLFMY-FLGTK--
PgmL5a-Ptet_PTE    1 ----------------------------GDNDNFQSQQVDKQQKIT-QLLQQPIIPAQVS
PgmL5a-Pbi_PBIG    1 ----------------------------GENDNFQSLQVDKQQKIT-QLLQQ-PIPPP-Q
PgmL5b-Ptet_PTE    1 ----------------------------GENDNFASHQIDKQQKIT-QLLQQ-PIPPPVQ
PgmL5b-Pbi_PBIG    1 ----------------------------GENDDFSSNQIDKQQKIT-QILQQ-PIPPP-Q
PgmL5b-Psex_PSE    1 ----------------------------GEIENLTLNQIDKQQKIT-QLLQQ-PIACP-Q
PgmL5a-Psex_PSE    1 ----------------------------GENENFSSNQMDKQQKIT-QLLQQ-PTPIP-Q
PgmL3c-Psex_PSE    1 -------------------------------SQYSAKKLIQLQTPQ-NIYDH-FIPNS--
PgmL3c-Ptet_PTE    1 ---------------------------------YHAKKIVQLQTPQ-NVYDH-FFPTS--
PgmL3c-Pbi_PBIG    1 -------------------------------SQYPAKRQVQPQTPQ-NTYDH-FFPNS--
PgmL3-Pcau_PCAU    1 -------------------------------LDAPQLKMSTLQSPK-NVIHY-FIPND--
PgmL3a-Pbi_PBIG    1 ---------------------------------TPQLKMTSFQSPQ-NVLNY-FLPSS--
PgmL3a-Ptet_PTE    1 ---------------------------------TPQLKMTSLQSPQ-NVLNY-FLPNS--
PgmL3b-Ptet_PTE    1 ---------------------------------TPQLKMTSLQSPQ-NVLNY-FLPSS--
PgmL3b-Pbi_PBIG    1 ---------------------------------TPQLKMTSLQSPL-NVLNY-FLPCS--
PgmL3a-Psex_PSE    1 -------------------------------LDTPQLKMTSLQSAQ-NVLNY-FLPSS--
PgmL4b-Psex_PSE    1 MNHKFLGMNQNQSRFSNSPQRVRDMITQQYTSSYQVDRPPIMPSRQQPVFQQQTLEKN--
PgmL4a-Psex_PSE    1 LNHKFLGMNQNQSRISNSPQRVRDMITQQYTSSYQVDRPPVMPSRQQPVFQQQILEKN--
PgmL4b-Ptet_PTE    1 INHKFLGMNQNQSRFSNSPPRVRDMITQQYTSSYQVDRPPIMPSRQQPIFQQQILEKN--
PgmL4b-Pbi_PBIG    1 LNHKFLGMNQNQSRISNSPSRVRDMITQQYTSSYQVDRPPNMPSRQQPIFQQQILEKN--
PgmL4a-Ptet_PTE    1 LNHKFLEMNQNQSRISNSPSRIRDMITLQYTSSYQVDRPPIMPSRQQPVFQQQTLERN--
PgmL4a-Pbi_PBIG    1 LNHKFLGMNQNQSRLSNSPSRVRDMITQQYTSSYQVDRPPNMPSRQQPVFQQQTLEKN--
Tpb7_TTHERM_006    1 --------------------------WKFKGQDKVNLEYGKNYDAS-TIFRM-LFTQP--
Pgbd5-Rty_XP_02    1 ---------------------------------GPVHQMPLNASAI-DFFQL-FVPDN--
Pgbd5-Dre_NP_00    1 ---------------------------------GPTQKMSATATAM-DFFQL-FVPDN--
Pgbd5-Hs_NP_001    1 ---------------------------------GPTRKMPPSASAV-DFFQL-FVPDN--
Pgbd5-Gga_XP_01    1 ---------------------------------GPTRKMPLTASAM-DFFQL-FVPDN--
Tpb1_TTHERM_000    1 --------------------------------KPQVKNIPLESTPV-QIFQK-LWSDE--
Tpb6_DAA80465.1    1 --------------------------------PPQLIGLDECKTPK-NFFDK-IWDDR--
Tpb2_TTHERM_011    1 ------------------------------DLNRDFIPYNVPHSPY-HLFRM-FFDDR--
Pgm-Pcau_PCAUDP    1 --------------------------------GPRSIEKNKIKSEY-DAFRL-FFDND--
Pgm-Pbi_PBIGNP2    1 --------------------------------GPRSIDKNKIKSEY-DAFRL-FFDND--
Pgm-Psex_PSEXPN    1 --------------------------------GPRSIDKNKIKSEY-DAFRL-FFDND--
Pgm-Ptet_PTET.5    1 --------------------------------GPRSIDKSKIKSEY-DAFRL-FFDND--
Pgbd3-Hs_NP_736    1 --------------------------------APPNDFFTVMRTPT-EILEL-FLDDE--
Pgbd1-Hs_NP_001    1 ------------------------FPSWSALDSGLLNLKSEKLNPV-ELFEL-FFDDE--
Pgbd2-Hs_NP_733    1 -------------------------------DPHIEDLKSQELSPV-GLFEL-FFDEG--
Bmo_BAD11135       1 ---------------------------------QVKNIARDASTEY-ECWNI-FVTSD--
Ago_ADU04477       1 ---------------------------------GPKECARNIVSEI-DAFLK-IIDLD--
Hvi_ABD76335       1 ---------------------------------GPKGRAKEIQTIS-EAFFC-MFSMD--
Pgo_ADB45159       1 ---------------------------------GPKERAREVSEPI-DIFSL-FISED--
Tni_AAA87375       1 ---------------------------------GPTRMCRNIYDPL-LCFKL-FFTDE--
Cag_ADV17598       1 ---------------------------------GPTRMCRNIVDPL-LCFQL-FIKEE--
Aip_ADV17599       1 ---------------------------------GPTRMCRNIVDPL-LCFQL-FITDE--
Har_ABS18391       1 ---------------------------------GPTRMCRNIVDPL-LCFQL-FITDE--
Hma_XP_00421184    1 --------------------------------GPTRFAAQVCGQRLDTAFKL-FITPE--
Tru_Pigibaku1_X    1 ---------------------------------GPTRMAVTHTQDIKSSFEL-FIPDS--
Oni_XP_00545891    1 --------------------------------GPTRYATSRIVDPI-SSFAL-LLTDE--
Pny_XP_00575514    1 --------------------------------GPTHYAVARISDPL-SNFRL-FLTDE--
Mlu_Mitra          1 -----------------------------------GLNTDAVINNIEDAVKL-FIGDD--
PLE-wu_ref         1 ---------------------------------TPTRVLPSNARPI-RYFEK-FFTQE--
Aca_XP_00509140    1 ---------------------------------GPLHNLDENSKPI-DFFHL-FVPPT--
Ami_ACT79641       1 ------------------------------SPVGITFEIGNEAREL-DVLKK-LFNDE--
Nap_EQB62075_PG    1 ------------GVEKWQAVKEITSVINEYLEEEKFLYDTDCNDPF-ALYKL-FFTDY--
Goc_XP_00374069    1 -------------------------------PQSSYLGDPQNLRPA-QVYRD-FFDKD--
Mro_XP_00370823    1 ------------------------------GAKTTNEKVRICTKAG-EFYAL-FVTDE--
Xbo_BAF82021_Ur    1 ------------------GPPCNYAPEIPPFTAVSGVKVDTTNFEIMDFFNL-FITEA--
Mfa_EHH62949_PG    1 -------------------------------TPGRKVDVSDITDPL-QYFEL-FFTEE--
Ggo_XP_00405596    1 -------------------------------TPGRKVDVSDITDPL-QYFEL-FFTEE--
Pgbd4-Hs_NP_689    1 -------------------------------TPGRKVDVSDITDPL-QYFEL-FFTEE--
Sbo_XP_00393580    1 -------------------------------TPGRKVDVSDITDPL-QYFEL-FFTEE--
consensus          1                                       l  i spv  if m fl
```

```
PgmL2b-Psex_PSE   30 -----------------------------------IVQSILKMI-NDQMKDHFSEQHEKD
PgmL2a-Psex_PSE   30 -----------------------------------IVQSILKMI-NDQMKDHFSEQHEKD
PgmL2-Ptet_PTET   30 -----------------------------------IVQSILKMI-NDQMKDHFSEQHEKD
PgmL2-Pbi_PBIGN   30 -----------------------------------IVQSILKMI-NDQMKDHFSEQHEKD
PgmL1-Pcau_PCAU   23 -----------------------------------LVKYMMDQI-NQKMRESFQNQSEKQ
PgmL1-Psex_PSEX   16 -----------------------------------LIRYLIDQI-NVKMKTSFANQDEKQ
PgmL1-Ptet_PTET   16 -----------------------------------LIRYLIDQI-NVKMKTSFANQDEKQ
PgmL1-Pbi_PBIGN   16 -----------------------------------LIRYLIDQI-NVKMKTSFANQDEKQ
Lia5_TTHERM_006   24 -----------------------------------ILRQLLNKA-NVKLREIYKKQNDVK
PgmL5a-Ptet_PTE   32 NKPLNQQQRKHQALLEEIVQEDDGIVQDIENQDWTLFSQLNNMV-PPKFSGQFRMLEYKD
PgmL5a-Pbi_PBIG   30 TKPLNQQQRKHQALLEEIVQEDDGIVQDIENQDWTLFTQLNSMI-APKFSGQFRMLEYKD
PgmL5b-Ptet_PTE   31 NKPLNQQQRKHQALLEEIVQEDDGIVQDIENQDWTLFTQLNNMI-TPKFSGQFRMLEYKD
PgmL5b-Pbi_PBIG   30 NKPLNQQQRKHQALLEEIVQEDDGVVQDIESQDWTLFTQLNTMI-TPKFSGQFRMLEYKD
PgmL5b-Psex_PSE   30 NKPLNQQYKKHQALLEEIVQEDDGIVQDVDYQDWSLFNQLNNMT-VPKFSGQFRMLEYKD
PgmL5a-Psex_PSE   30 AKPLNQQQKKHQALLDDIVQEDDGIVQDIDYQDWSLFNQANNMT-APKFSGQFRMLEYKD
PgmL3c-Psex_PSE   26 -----------------------------------LIGTLTELV-NQYLLFILEQDLKSQ
PgmL3c-Ptet_PTE   24 -----------------------------------LIVTLTDLV-NQYLFSILEEDLKQQ
PgmL3c-Pbi_PBIG   26 -----------------------------------MIVTLTDLV-NQYLLAILEEDLKQQ
PgmL3-Pcau_PCAU   26 -----------------------------------FISSLTEMI-NKYLMAALLQDQKQQ
PgmL3a-Pbi_PBIG   24 -----------------------------------FVSTITEMI-NHYLLMVLQEDLKQQ
PgmL3a-Ptet_PTE   24 -----------------------------------FVSTLTEMI-NHYLLMVLQEDVKQQ
PgmL3b-Ptet_PTE   24 -----------------------------------FVSTLTEMI-NHYLLTVLQEDLKQQ
PgmL3b-Pbi_PBIG   24 -----------------------------------FISTLTEMI-NHYLLMVLQEDLKQQ
PgmL3a-Psex_PSE   26 -----------------------------------FVSTLTEMI-NHYLLMVLQEDLKQQ
PgmL4b-Psex_PSE   59 -----------------------------------INYELNQVKKNKEQLSSHQQSQLRE
PgmL4a-Psex_PSE   59 -----------------------------------INYEINQVKKNKEQLSSHQQSQLRE
PgmL4b-Ptet_PTE   59 -----------------------------------ITYELNQVKKNKEQLSSHQQSQLRE
PgmL4b-Pbi_PBIG   59 -----------------------------------INYELNQVKKNKEQLSSHQQSQLRE
PgmL4a-Ptet_PTE   59 -----------------------------------INYELNQAKKNKEQLSGHQQSQLRE
PgmL4a-Pbi_PBIG   59 -----------------------------------INYELNQAKKNKEQLSSHQQSQLRE
Tpb7_TTHERM_006   31 -----------------------------------LYTHIIQNIYKKFNKRITKRNIFKD
Pgbd5-Rty_XP_02   24 -----------------------------------VIRNMVVQT-NMYAKKYQERFGCDE
Pgbd5-Dre_NP_00   24 -----------------------------------VIQNMVTQT-NMYAKKFQERFGSDE
Pgbd5-Hs_NP_001   24 -----------------------------------VLKNMVVQT-NMYAKKFQERFGSDG
Pgbd5-Gga_XP_01   24 -----------------------------------VLKNMVVQT-NMYAKKYQERFGSDD
Tpb1_TTHERM_000   25 -----------------------------------IWKLITDET-NKYSKQSFDLNQYNL
Tpb6_DAA80465.1   25 -----------------------------------IWEMLTVYS-NIYAEQYFQKKGIRM
Tpb2_TTHERM_011   27 -----------------------------------IYKLIIEET-NRYKHQKYQEALLNL
Pgm-Pcau_PCAUDP   25 -----------------------------------IYATIIKHTRERYQQKVEEQIYSYI
Pgm-Pbi_PBIGNP2   25 -----------------------------------IYNTIIKHTRERYQQKVEEQIYSYI
Pgm-Psex_PSEXPN   25 -----------------------------------IYNTIIKHTRERYQQKVEEQIYSYI
Pgm-Ptet_PTET.5   25 -----------------------------------IYNTIIKHTRERYQQKVEEQIYSYI
Pgbd3-Hs_NP_736   25 -----------------------------------VIELIVKYS-NLYACSKGVHLG---
Pgbd1-Hs_NP_001   33 -----------------------------------TFNLIVNET-NNYASQKNVSLE---
Pgbd2-Hs_NP_733   26 -----------------------------------TINFIVNET-NRYA--------WQK
Bmo_BAD11135      24 -----------------------------------MLQEILTHT-NSSIRHRQTKTAAEN
Ago_ADU04477      24 -----------------------------------MIDEIVTCT-NMYISNMRQRVQYSR
Hvi_ABD76335      24 -----------------------------------TVNLVLQQT-NDYIKSIQEKFQRER
Pgo_ADB45159      24 -----------------------------------MLQQVVTFT-NAEMLIRKNKYKTET
Tni_AAA87375      24 -----------------------------------IISEIVKWT-NAEISLKRRESMTGA
Cag_ADV17598      24 -----------------------------------IVEEIVKWT-NVEMVQKRVNLKDIS
Aip_ADV17599      24 -----------------------------------IIHEIVKWT-NVEMIVKRQNLIDIS
Har_ABS18391      24 -----------------------------------IIHEIVKWT-NVEIIVKRQNLKDIS
Hma_XP_00421184   26 ------------------------------------IRIIVNCT-NAEARRIRLEG----
Tru_Pigibaku1_X   25 -----------------------------------IQEIILDCT-NLEGRRVFGER----
Oni_XP_00545891   25 -----------------------------------IVQHIVSMT-NLHGKRKIPG-----
Pny_XP_00575514   25 -----------------------------------IMHHIVEMT-NLHGRRTISD-----
Mlu_Mitra         23 -----------------------------------FFEFLVEES-NRYYNQNRNNFKLSK
PLE-wu_ref        24 -----------------------------------VFELIITET-NRYACQNNVIG----
Aca_XP_00509140   24 -----------------------------------FLDEIVVQT-NLYAEQCQNNKGKRD
Ami_ACT79641      27 -----------------------------------ILNVIVRET-NRHARQKLAGDALDK
Nap_EQB62075_PG   45 -----------------------------------ILEMIVEET-NKYATQGIKNSSSSS
Goc_XP_00374069   26 -----------------------------------LVRMIVAET-NRNAKNYIDANTIGR
Mro_XP_00370823   27 -----------------------------------IFQHISEQT-NHYATQCRIISKRTT
Xbo_BAF82021_Ur   40 -----------------------------------ILQDMVHFT-NLYAEQYLASHSLPV
Mfa_EHH62949_PG   26 -----------------------------------LVSKITRET-NAQAALLASKPPGPK
Ggo_XP_00405596   26 -----------------------------------LVSKITRET-NAQAALLASKPPGPK
Pgbd4-Hs_NP_689   26 -----------------------------------LVSKITRET-NAQAALLASKPPGPK
Sbo_XP_00393580   26 -----------------------------------LVSKITRET-NAQAALLASKPPGPK
consensus         61                                    iv  il  t n y
```

```
PgmL2b-Psex_PSE   54 VKKRL-----------------------IKLDQIYEYFGVKILMGYNRMP----------
PgmL2a-Psex_PSE   54 VKKRL-----------------------FKLDQIYEYFGVKILMGYNRMP----------
PgmL2-Ptet_PTET   54 VKKRL-----------------------FKLDQIYEYFGVKILMGYNRMP----------
PgmL2-Pbi_PBIGN   54 VKKRL-----------------------FKLDQIYEYFGVKILMGYNRMP----------
PgmL1-Pcau_PCAU   47 IKKKL-----------------------IKIDEIYDFFGIKIIMGHIKMP----------
PgmL1-Psex_PSEX   40 IRKKL-----------------------VKIDQIFDFFGIKIIMGYIKMP----------
PgmL1-Ptet_PTET   40 IRKKL-----------------------VKIDQIFDFFGIKIIMGYIKMP----------
PgmL1-Pbi_PBIGN   40 IRKKL-----------------------VKIDQIFDFFGIKIIMGYIKMP----------
Lia5_TTHERM_006   48 E---------------------------YTIEEIQVYQALKILMGLQQNS----------
PgmL5a-Ptet_PTE   91 NPQDMIRLLFGET----RFK-DLMKTCQTSEPEFWLYLGTKLIMGYMRLP----------
PgmL5a-Pbi_PBIG   89 NSQDMIRLLFGET----RFK-ELMKTCQTSEPEFWLYLGTKLIMGYMRLP----------
PgmL5b-Ptet_PTE   90 NTQDMIRLLFGET----RFK-DLMKTCQTSESEFWLYLGTKLIMGYMRLP----------
PgmL5b-Pbi_PBIG   89 NTQDMIRLLFGET----RFK-DLMKTCQTSESEFWLYLGTKLIMGYMRLP----------
PgmL5b-Psex_PSE   89 NPQEMIRLLFGES----RFK-DLMKTCQTNESEFWLYLGTKLIMGYMRLP----------
PgmL5a-Psex_PSE   89 NPQDMIRLLFGEI----RFK-DLMKTCQTSESEFWLYLGTKLIMGYMRLP----------
PgmL3c-Psex_PSE   50 PSNIQRQFI--------KKK--------YKETEIQLYFGLQILFGIYRFP----------
PgmL3c-Ptet_PTE   48 PSKIASQYN--------KKK--------YKETEIQLYFGLQILFGIFRFP----------
PgmL3c-Pbi_PBIG   50 PSKIPSQYH--------KKK--------YKETEIQLYFGLQILFGIFRFP----------
PgmL3-Pcau_PCAU   50 TQQTQQLYN--------KKK--------YKDTEIQFYLGLQILFGMYRFP----------
PgmL3a-Pbi_PBIG   48 HQTQQIYH---------KKK--------YKESEIQFYLGLQILFGVYRFP----------
PgmL3a-Ptet_PTE   48 QQTQQIYH---------KKK--------YKEAEIQFYLGLQILFGVYRFP----------
PgmL3b-Ptet_PTE   48 QQQTQQIYH--------KKK--------YKEAEIQFYLGLQILFGVYRFP----------
PgmL3b-Pbi_PBIG   48 QQQTQQIYH--------KKK--------YKEAEIQFYLGLQILFGVYRFP----------
PgmL3a-Psex_PSE   50 QQQTQQMYH--------KKK--------YKEAEIQFYLGLQILFGVYRFP----------
PgmL4b-Psex_PSE   84 SQIKLQAKLQESVK---RQSVAQPIIEDNDSDFMDDVMESQAFVDFKRAGNLINGSQQ--
PgmL4a-Psex_PSE   84 SQIKLQAKLQESVK---RQSVAQPTIEENDSDFMDDIMDSQAFIDFKRAGNLMCGSQQSL
PgmL4b-Ptet_PTE   84 SQIKLQAKLQESVK---RQSVAQPTIEENDSDFLDEVMDSQAFIDFKKAGNLICGTQQS-
PgmL4b-Pbi_PBIG   84 SQIKLQAKLQESVK---RQSVAQPTIEENDSDFMDDVMDSQAFIDFKRAGNLICGSQQQ-
PgmL4a-Ptet_PTE   84 SQIKLQAKIQESVK---RQSVVQPTIEENDSDFMDDGMDSQPFIDFKRAGNIPCASQQ--
PgmL4a-Pbi_PBIG   84 SQIKLQAKLQESVK---RQSNVQPTIEENDSDFMDDVMDSQAFIDFKRAGNLMCGSQQ--
Tpb7_TTHERM_006   56 PIE-------------------------INSYDIDAYISLLLHSQCLQHP----------
Pgbd5-Rty_XP_02   48 G----------------WSD--------VDLPEMKTFLGYMISTSIHHCE----------
Pgbd5-Dre_NP_00   48 G----------------WTN--------VTLAEMKAFLGYVTSTSVNRCE----------
Pgbd5-Hs_NP_001   48 A----------------WVE--------VTLTEMKAFLGYMISTSISHCE----------
Pgbd5-Gga_XP_01   48 T----------------WID--------VTLTEMKAFLGYMISTSIHHCE----------
Tpb1_TTHERM_000   49 DSQSLKKQKICF----------------FSQDQIKRFIICEILMGIQRLP----------
Tpb6_DAA80465.1   49 DQTFKDQIKQDKI----LYQ-LLKHFAPFTKNDIKRFIICEILQGIKRLP----------
Tpb2_TTHERM_011   51 PPDKTLPKE--------AMD--------IDSIDLDAYLSILIFMGVQRMK----------
Pgm-Pcau_PCAUDP   50 HGMVHMGIRAKKPSLMQWE---------FTEYELEAYFAVQIFFGIVRLS----------
Pgm-Pbi_PBIGNP2   50 HGMVHMGIRAKKPSLMQWE---------FTEYELEAYFAVQIFFGIVRLS----------
Pgm-Psex_PSEXPN   50 HGMVHMGIRAKKPSLMQWE---------FTEYELEAYFAVQIFFGIVRLS----------
Pgm-Ptet_PTET.5   50 HGMVHMGIRAKKPTLMQWE---------FTEYELEAYFAVQIFFGIVRLS----------
Pgbd3-Hs_NP_736   46 ----------------------------LTSSEFKCFLGIIFLSGYVSVP----------
Pgbd1-Hs_NP_001   54 ----------------------------VTVQEMRCVFGVLLLSGFMRHP----------
Pgbd2-Hs_NP_733   42 NVNLS-----------------------LTAQELKCVLGILILSGYISYP----------
Bmo_BAD11135      48 SSAETSFY---------MQE--------TTLCELKALIALLYLAGLIKSNRQ--------
Ago_ADU04477      48 PRDCLD----------------------TSRCEILAYFGLLFLIGIKKAHHA--------
Hvi_ABD76335      48 DCKV------------------------LEYEELLAYLGLLYMSGVLRSSHL--------
Pgo_ADB45159      48 FTVSP-----------------------TNLEEIRALLGLLFNAAAMKSNHL--------
Tni_AAA87375      48 TFRD------------------------TNEDEIYAFFGILVMTAVRKDNHM--------
Cag_ADV17598      48 AS---------------YRD--------TNEMEIWAIISMLTLSAVMKDNHL--------
Aip_ADV17599      48 AS---------------YRD--------TNTMEMWALVGILTLTAVMKDNHL--------
Har_ABS18391      48 AS---------------YRD--------TNTMEIWALVGILTLTAVMKDNHL--------
Hma_XP_00421184   45 -----------------WVD--------TTVNELFEFIGVLLLAGVFHSKNQ--------
Tru_Pigibaku1_X   45 -----------------WKE--------LDQTQLHAYFGVLILAGVFRSKGE--------
Oni_XP_00545891   44 -----------------WRD--------IDAEEFRAYVGLLVLSGVYRSKHE--------
Pny_XP_00575514   44 -----------------WRD--------LDNDEILAYVGLLILAGVYRSKHE--------
Mlu_Mitra         47 KSLK-------------WKD--------ITPQEMKKFLGLIVLMGQVRKD----------
PLE-wu_ref        44 -----------------WTI--------LDIKELKAFLGILIIMGYNILP----------
Aca_XP_00509140   48 TY---------------WKA--------VTVSDIRKFLYLNIMFGIHHVP----------
Ami_ACT79641      51 -----------------WQD--------VTLEEIKAFLGVSVVMGVNILP----------
Nap_EQB62075_PG   69 RIHQKA-----------WQS--------VTKDEVNTFIGILLIMGVVQLP----------
Goc_XP_00374069   50 SSRAQR-----------WED--------TSSDEIEKFFGIVLYMGLVPYP----------
Mro_XP_00370823   51 N----------------WTP--------TNKNELKRLFGLLIWMGMVNLP----------
Xbo_BAF82021_Ur   64 YSRAQA-----------WYP--------TNVNEIKKFLALTLAMGLVELN----------
Mfa_EHH62949_PG   50 GFSRMDK----------WKD--------TDNDELKVFFAVMLLQGIVQKP----------
Ggo_XP_00405596   50 GFSRMDK----------WKD--------TDNDELKVFFAVMLLQGIVQKP----------
Pgbd4-Hs_NP_689   50 GFSRMDK----------WKD--------TDNDELKVFFAVMLLQGIVQKP----------
Sbo_XP_00393580   50 GFSRMDK----------WKD--------TDNDELKVFFAVMLLQGIVQKP----------
consensus        121                                 ei  ylgv ilmglir p
```

```
PgmL2b-Psex_PSE    81 -DPEDYFNYK--------------------------------------------------
PgmL2a-Psex_PSE    81 -DPEDYFNDK----------------------------L------PFRNKI-GEL-----
PgmL2-Ptet_PTET    81 -DPEDYFNDK----------------------------L------PFRNKI-GEL-----
PgmL2-Pbi_PBIGN    81 -DPEDYFNDK----------------------------L------PFRNKI-GEL-----
PgmL1-Pcau_PCAU    74 -TLEDYFDET----------------------------Q------LFQCKIFDC------
PgmL1-Psex_PSEX    67 -HLEDYFNEK----------------------------Q------LFQCKIFEC------
PgmL1-Ptet_PTET    67 -HLEDYFNEK----------------------------Q------LFQCKIFEC------
PgmL1-Pbi_PBIGN    67 -HLEDYFNEK----------------------------Q------LFQCKIFEC------
Lia5_TTHERM_006    71 -SNFDFFLLK----------------------------D------LQKIPPYHQL-----
PgmL5a-Ptet_PTE   136 -DINEYFYGE----------------------------D------WIAGGGIKSI-----
PgmL5a-Pbi_PBIG   134 -DINEYFYGE----------------------------D------WIAGGGIKSI-----
PgmL5b-Ptet_PTE   135 -DINEYFYGE----------------------------D------WIAGGGIKSI-----
PgmL5b-Pbi_PBIG   134 -DINEYFYGE----------------------------D------WIAGGGIKSI-----
PgmL5b-Psex_PSE   134 -DINEYFYGE----------------------------D------WIAGGGIKSI-----
PgmL5a-Psex_PSE   134 -DINEYFYGE----------------------------D------WIAGGGIKSI-----
PgmL3c-Psex_PSE    84 -QIDDYWNAE----------------------------P------WLRSGI-EYM-----
PgmL3c-Ptet_PTE    82 -YIDDYWNAE----------------------------P------WLRCGI-EYM-----
PgmL3c-Pbi_PBIG    84 -YIDDYWNQE----------------------------P------WLRCGI-EHM-----
PgmL3-Pcau_PCAU    84 -QLDEYWNAE----------------------------P------WLRGGI-EIS-----
PgmL3a-Pbi_PBIG    81 -SLEEYWNAE----------------------------S------WLKGGV-EVT-----
PgmL3a-Ptet_PTE    81 -SLDDYWNAE----------------------------S------WLKGGV-EVT-----
PgmL3b-Ptet_PTE    82 -QLDDYWNAE----------------------------S------WLRGGV-EVT-----
PgmL3b-Pbi_PBIG    82 -SLDDYWNAE----------------------------S------WLKGGV-EVT-----
PgmL3a-Psex_PSE    84 -SLDDYWNVD----------------------------S------WLKGGV-EVT-----
PgmL4b-Psex_PSE   139 SNQQQYWQEINFNTDIPKEYYMNEKFRVEISQDIEISDPYELFKLYFDQSIFKYICQISN
PgmL4a-Psex_PSE   141 QQQQQYWQEINFNTDIPKDYYMNEKFRVDVVEDIDINDPYELFKMYFDQSIFKYICQISN
PgmL4b-Ptet_PTE   140 -IQQQHWQEINFNTDIPKDYYMNEKFRVDVGEDIEICDPYELFKLYFDQSIFKYICQISN
PgmL4b-Pbi_PBIG   140 QIQQYYWQEINFNTDIPKDYYMNEKFRVDVGQDIEIFDPYELFKLYFDQSIFKYICQISN
PgmL4a-Ptet_PTE   139 QTYQQYWQEINFNTDIPKDYYMNEKFRVDLEQEIETSDPYELFKLYFDQSIFKYICLISN
PgmL4a-Pbi_PBIG   139 QMQQQYWQEINFNTDIPKDYYMNEKFRVDLEQEIETSDPYELFKLYFDQSISKYICLISN
Tpb7_TTHERM_006    81 -NLINTINKI------------------------------------------NQD-----
Pgbd5-Rty_XP_02    74 -SVLSIWSGG-----------------------------------FYSNKSIALM-----
Pgbd5-Dre_NP_00    74 -SVLSIWSSG-----------------------------------FFSNRSIALK-----
Pgbd5-Hs_NP_001    74 -SVLSIWSGG-----------------------------------FYSNRSLALV-----
Pgbd5-Gga_XP_01    74 -SVLSIWSSG-----------------------------------FYSNKSIALI-----
Tpb1_TTHERM_000    83 -SFSDYFSSD----------------------------P------LLSGGL-NRI-----
Tpb6_DAA80465.1    94 -CFLDYWSTN----------------------------P------LISGGL-NRF-----
Tpb2_TTHERM_011    85 -SVKDYWKRK----------------------------N------YINSEI-ACI-----
Pgm-Pcau_PCAUDP    91 -NQRDYWKSSARQRPIKKAETGRRKLRELAMEKMDRYAH------WVTQRM-SSI-----
Pgm-Pbi_PBIGNP2    91 -NQRDYWKSSARQKPIKKAETGRRKLRELAQEKMDRYAH------WVTQRM-SSI-----
Pgm-Psex_PSEXPN    91 -NQRDYWKSSARQKPIKKAETGRRKLRELAQEKMDRYAH------WVTQRM-SSI-----
Pgm-Ptet_PTET.5    91 -NQRDYWKSSARQKPIKKAETGRRKLRELAQEKMDRYAH------WVTQRM-SSI-----
Pgbd3-Hs_NP_736    68 -RRRMFWEQR----------------------------T------DVHNVLVSAA-----
Pgbd1-Hs_NP_001    76 -RREMYWEVS-----------------------------------DTDQNLVRDA-----
Pgbd2-Hs_NP_733    69 -RRRMFWETS----------------------------P------DSHHHLVADA-----
Bmo_BAD11135       83 -SLKDLWRTD-----------------------------------GTGVDIFRTT-----
Ago_ADU04477       78 -NVKELWSAD-----------------------------------GSGTEITRAT-----
Hvi_ABD76335       76 -NFKDLWATD-----------------------------------GTGIEFFQNT-----
Pgo_ADB45159       77 -PTRMLFNTH------------------------------------RSGTIFKAC-----
Tni_AAA87375       76 -STDDLFDRS-------------------------------------LSMVYVSV-----
Cag_ADV17598       77 -STDELFNVS------------------------------------YGTRY-VSV-----
Aip_ADV17599       77 -STDELFDAT------------------------------------FSGTRYVSV-----
Har_ABS18391       77 -STDELFDAT------------------------------------FSGTRYVSV-----
Hma_XP_00421184    72 -NIKEFWSK------------------------------------LDGIPIFSIS-----
Tru_Pigibaku1_X    72 -SAESLWDAE------------------------------------TGREIFRAT-----
Oni_XP_00545891    71 -STISLWSEK------------------------------------WGRSIFRAT-----
Pny_XP_00575514    71 -ATTSLWSEK------------------------------------TGRSIFRAA-----
Mlu_Mitra          76 -RRDDYWTTE----------------------------P------WTETPYFGKT-----
PLE-wu_ref         69 -TFELYWSSD----------------------------P------EFRVDEIAST-----
Aca_XP_00509140    75 -DSRLYWSVD----------------------------P------VLRVPAVADV-----
Ami_ACT79641       76 -SISDYWSSN----------------------------Q------FLGNEGIQKV-----
Nap_EQB62075_PG   100 -EIRLYWSNK----------------------------D------MYANARIKKA-----
Goc_XP_00374069    81 -SIPDYWSTN----------------------------D------LYGNRIVATT-----
Mro_XP_00370823    77 -SLRLYWSQD----------------------------P------LFSQTFPRTI-----
Xbo_BAF82021_Ur    95 -TIASYWDTT----------------------------T------VISVPLFSAV-----
Mfa_EHH62949_PG    82 -ELEMFWSTR----------------------------P------LLDTPYLRQI-----
Ggo_XP_00405596    82 -ELEMFWSTR----------------------------P------LLDTPYLRQI-----
Pgbd4-Hs_NP_689    82 -ELEMFWSTR----------------------------P------LLDTPYLRQI-----
Sbo_XP_00393580    82 -ELEMFWSTR----------------------------P------LLDTPYLRQI-----
consensus         181   l dyw                                      w    i   l
```

```
PgmL2b-Psex_PSE   90 --------------------------------INKSRKIQIFRINTNLRDVVMIQAKYHN
PgmL2a-Psex_PSE  100 --------------------------------IKVGR-FKFLEQNTNLRDEVMIQAKYHN
PgmL2-Ptet_PTET  100 --------------------------------IKVGR-FKFLEQNTNLRDEVMIQAKYHN
PgmL2-Pbi_PBIGN  100 --------------------------------IKVGR-FKFLEQNTNLRDEVMIQAKYHN
PgmL1-Pcau_PCAU   93 --------------------------------FKEGR-FKCLEQWSCFTDQELLRKE-NK
PgmL1-Psex_PSEX   86 --------------------------------FKEGR-FKCLEQWSNFTDESLLKNINKV
PgmL1-Ptet_PTET   86 --------------------------------FKEGR-FKCLEQWSNFTDECLLKNINKA
PgmL1-Pbi_PBIGN   86 --------------------------------FKEGR-FKCLEQWSNFSDECLLKNINKV
Lia5_TTHERM_006   91 --------------------------------FTNER-FQFLLDCER------------E
PgmL5a-Ptet_PTE  156 --------------------------------ITQQQ-FDDIDQKVEIIQPKIQTES-QL
PgmL5a-Pbi_PBIG  154 --------------------------------ITQQQ-FDDIDQRVEIIQPKLQTES-QL
PgmL5b-Ptet_PTE  155 --------------------------------ITQQQ-FDDIDQRVEIIQPKLQTES-QL
PgmL5b-Pbi_PBIG  154 --------------------------------ITQQQ-FEDIDQRLEIIQPKLQTES-QL
PgmL5b-Psex_PSE  154 --------------------------------ITQQQ-FEDIDQKVEIIQPKLQTES-QL
PgmL5a-Psex_PSE  154 --------------------------------ITQQQ-FDDIDQKVEIIQPKLQTES-QL
PgmL3c-Psex_PSE  103 --------------------------------MPIGR-FKFVDTHLFL-----------Y
PgmL3c-Ptet_PTE  101 --------------------------------MPIGR-FKFLDTHLFM-----------Y
PgmL3c-Pbi_PBIG  103 --------------------------------MPIGR-FKFLDTHLFM-----------Y
PgmL3-Pcau_PCAU  103 --------------------------------MPIGR-LKFVDMYLCSHDEET-------
PgmL3a-Pbi_PBIG  100 --------------------------------MPIGR-FKFVDLHIFS-----------F
PgmL3a-Ptet_PTE  100 --------------------------------MPIGR-FKFVDLHIFS-----------Y
PgmL3b-Ptet_PTE  101 --------------------------------MPIGR-FKFVDLHIFS-----------Y
PgmL3b-Pbi_PBIG  101 --------------------------------MPIGR-FKFVDLHIFS-----------Y
PgmL3a-Psex_PSE  103 --------------------------------MPIGR-FKFVDLHIFS-----------Y
PgmL4b-Psex_PSE  199 KRQCIRIDEDILESFVSALIYIFYIQLLGMREIKRVR-FDHILDYVTFGHICK------E
PgmL4a-Psex_PSE  201 KRQCIRIDEDILESFISALIYIFYIQLLGMREIKRVR-FDHIIDYVTFGHICK------E
PgmL4b-Ptet_PTE  199 KRQCIRIDEDILESFITALIYIFYIQLLGMREIKRVR-FDHIIDYVTFGHICK------E
PgmL4b-Pbi_PBIG  200 KRQCIRIDEDILESFISALIYIFYIQLLGMREIKRVR-FDHILDYVTFGHICK------E
PgmL4a-Ptet_PTE  199 KRQCIRIDEDILESFISALIYIFYIQLLGMREIKRVR-FDHILDYVTFGHICK------E
PgmL4a-Pbi_PBIG  199 KRQCIKIDEDILESFISALIYIFYIQLLGMREIKRVR-FDHILDYVTFGHICK------E
Tpb7_TTHERM_006   93 --------------------------------MDHHI-LEFIQNNFSVGAKVQDE----N
Pgbd5-Rty_XP_02   93 --------------------------------MTQMR-FEKILKYFHIVAFRS------S
Pgbd5-Dre_NP_00   93 --------------------------------MSQAR-FEKILKYFHVVAFRSS-----Q
Pgbd5-Hs_NP_001   93 --------------------------------MSQAR-FEKILKYFHVVAFRS------S
Pgbd5-Gga_XP_01   93 --------------------------------MTQSR-FEKILKYFHIVAFRS------S
Tpb1_TTHERM_000  102 --------------------------------LGREN-YQLLTRYLHISDNQSR-----M
Tpb6_DAA80465.1  113 --------------------------------ISKMK-YNICVMFFHASDSSDI-----S
Tpb2_TTHERM_011  104 --------------------------------MKYSR-FEQIDKHLHLADNEDP-----K
Pgm-Pcau_PCAUDP  138 --------------------------------VSYER-FKTIRNCLNISGAEALK----L
Pgm-Pbi_PBIGNP2  138 --------------------------------VSYEK-FKTIRNCLNISGAEALK----L
Pgm-Psex_PSEXPN  138 --------------------------------VSYEK-FKTIRNCLNISGAEALK----L
Pgm-Ptet_PTET.5  138 --------------------------------VSYEK-FKTIRNCLNISGAEALK----L
Pgbd3-Hs_NP_736   88 --------------------------------MRRDR-FETIFSNLHVADNAN------L
Pgbd1-Hs_NP_001   95 --------------------------------IRRDR-FELIFSNLHFADNGH------L
Pgbd2-Hs_NP_733   89 --------------------------------IRRDR-FELIFSYLHFADNNE------L
Bmo_BAD11135     102 --------------------------------MSLQR-FQFLQNNIRFDDKSTRDE---R
Ago_ADU04477      97 --------------------------------MSYKR-FLFLCRCLRFDDRGTRAE---R
Hvi_ABD76335      95 --------------------------------MSFNR-FLFISRCVRFDDKNTKSE---R
Pgo_ADB45159      95 --------------------------------MSAER-LNFLIKCLRFDDKLTRNV---R
Tni_AAA87375      93 --------------------------------MSRDR-FDFLIRCLRMDDKSIRPT---L
Cag_ADV17598      94 --------------------------------MSRER-FEFLLRLLRMGDKLLRPN---L
Aip_ADV17599      95 --------------------------------MSRER-FEFLIRCMRMDDKTLRPT---L
Har_ABS18391      95 --------------------------------MSRER-FEFLIRCIRMDDKTLRPT---L
Hma_XP_00421184   90 --------------------------------MQRDR-FVNLQRCIRFDEREIRNQ---R
Tru_Pigibaku1_X   90 --------------------------------MSLEN-FHIISRIMRFDNRDDRPA---R
Oni_XP_00545891   89 --------------------------------MSQKR-FQHISRALRFDDKLSRP----P
Pny_XP_00575514   89 --------------------------------MSQKR-FSHITSALRFDDKLSRP----R
Mlu_Mitra         96 --------------------------------MTRDR-FRQIWKAWHFNNNADI-----V
PLE-wu_ref        89 --------------------------------MTFRR-FKQILRCLHLNDNSKQPAR-LS
Aca_XP_00509140   95 --------------------------------MSRQR-FEKINQYFHLNDSTKMPNR-GE
Ami_ACT79641      96 --------------------------------MTKNR-YENISRFFHFNDSSVEPRR-GE
Nap_EQB62075_PG  120 --------------------------------MKRDR-FLSILKFLHFADNTT------A
Goc_XP_00374069  101 --------------------------------MSRNR-FQLLLRFVHFSNNQK------E
Mro_XP_00370823   97 --------------------------------MSRDR-FEILMRMVHFADNEA------A
Xbo_BAF82021_Ur  115 --------------------------------MPRNR-YQILLRFLHFNDNAAAVPP-NE
Mfa_EHH62949_PG  102 --------------------------------MTGER-FLLLFRCLHFVNNSSISAG-QS
Ggo_XP_00405596  102 --------------------------------MTGER-FLLLFRCLHFVNNSSISAG-QS
Pgbd4-Hs_NP_689  102 --------------------------------MTGER-FLLLFRCLHFVNNSSISAG-QS
Sbo_XP_00393580  102 --------------------------------MTGER-FLLLFRCLHFVNNSSISAG-QS
consensus        241                                 mt  r f  i   vrl d
```

```
PgmL2b-Psex_PSE   118 LKQEDVENKFKVEGEVYSYLTKKLNKKFRILYDAGQCLAIIK--------------NNLF
PgmL2a-Psex_PSE   127 LKQEDVEKKFKVEGEVYSYLTKKLNKKFRISHDAGQCLAIIK--------------NNLF
PgmL2-Ptet_PTET   127 LKQEDVEKKFKVEGEVYSYLTKKLNKKFRISHDAGQCLAVIK--------------NNLF
PgmL2-Pbi_PBIGN   127 LKQEDVEKKFKVEGEVYSYLTKKLNKKFRISHDAGQCLAVIK--------------NNLF
PgmL1-Pcau_PCAU   119 QSLNEFSATRVC---------KKIGKIAISVKNPGRDLILQV--------NPGFIVQNG-
PgmL1-Psex_PSEX   113 KPSNEFQTIRVC---------KKIGKTLKNFKAPGKDLILQV--------NTGFIIESQI
PgmL1-Ptet_PTET   113 KPINEFQTIRVC---------KKIGKTLRNIKTPGKDLILQV--------NSGFIIEQSI
PgmL1-Pbi_PBIGN   113 KPINEFQTIRVC---------KKIGKTLRNIKTSGKDLILQV--------NSGFMIEQSI
Lia5_TTHERM_006   106 LNKDILNNEQLI---------QDFVQRAQNSQTSDQELVLIS--------KKGKVGEEII
PgmL5a-Ptet_PTE   182 AVKLEFNAQSFFRQEFLPQFTQELNDRFKKLILPGQELFLISNF------------YTII
PgmL5a-Pbi_PBIG   180 AVKLEFNAQSFFRQEYLPQFTQELNDRFKKLILPGQELFLISNFYT------------LI
PgmL5b-Ptet_PTE   181 AVKLEFNAQTFFRQEYLPQFTQELNDRFKKLILPGQELFLISNF------YT-----LIY
PgmL5b-Pbi_PBIG   180 AVKLEFNAQAFFRQEYLPQFTQELNDRFKKLILPGQELFLIS----------NFYTL-IY
PgmL5b-Psex_PSE   180 AVKLEFNAQTFFRQEFLPQFTQELNDRFKKLILPGQELFLISNF------YT-----LIY
PgmL5a-Psex_PSE   180 AVKLEFNAQTFFRQEYLPQFTQELNDRFKKLILPGQELFLIS----------NFYTL-IY
PgmL3c-Psex_PSE   119 LNEKAKSKLQDEI--------QRLQKSVKCLCQPEQELILIEQI------YKAYIA----
PgmL3c-Ptet_PTE   117 LNQNAKSKLLNEV--------LQFQKQVKSICQPEQELILIEQI------YKAYTA----
PgmL3c-Pbi_PBIG   119 LNEKSKSKLQNEL--------QQLQKSVKSICQPEQELILIEQI------YKAYTV----
PgmL3-Pcau_PCAU   123 ----QRATISSQL--------KKFKQKIKALYNPDQELVVTQQI------YKAYTT----
PgmL3a-Pbi_PBIG   116 FDELQRAQLQLEV--------SKFSKKLKALYNPDQELIVVE------------------
PgmL3a-Ptet_PTE   116 FDEQQRAKLQLEV--------SKFSKKLKSLYNPDQELIIVE------------------
PgmL3b-Ptet_PTE   117 FDEVQRAKLQLEV--------RKFSKKLKALYNPDQELIVVE------------------
PgmL3b-Pbi_PBIG   117 FDELQRAKLQLEV--------RKFSRKLKALYNPDQELIVVE------------------
PgmL3a-Psex_PSE   119 FDELQRAKLQLEV--------RKFSKKLKALYNPDQELIVVE------------------
PgmL4b-Psex_PSE   252 IRIDELEDYSFIF--------EKISKNFKNYYQPEEFLTLDSPIF-----YQNHSVEGL-
PgmL4a-Psex_PSE   254 IRIDELEDYSFIF--------EKISKNFKNLYQPDEFLTLDSPIF-----YQNHCVEGL-
PgmL4b-Ptet_PTE   252 IRIDELEDYSFIF--------EKISKNFKNNYQPEEFLTLDSPIF-----YQNHSVEGL-
PgmL4b-Pbi_PBIG   253 IRIDELEDYSFIF--------EKISKNFKNNYQPEEFLTLDSPIF-----YQNHSVEGL-
PgmL4a-Ptet_PTE   252 IRIDELEDYSFIF--------EKISKNFKSHYQPEEFLTLDSPIF-----YQNHSVDGL-
PgmL4a-Pbi_PBIG   252 IRIDELEDYSFIF--------EKISKNFKNHYQPEEFLTLDSPIF-----YQNHSVEGL-
Tpb7_TTHERM_006   116 GCFNEIAKIEQFI--------SMVNQRLKQLVQPKEYLQLLH-----------KTIEGNY
Pgbd5-Rty_XP_02   114 QTTHGLYKIQPFL--------DCLQMTFDTAFKPSQTQVLHEPLIDEDPLFIATCTDREL
Pgbd5-Dre_NP_00   115 GGSQGLYKIQPFL--------DSLQQSFSSSFRPSQTQVLHEPLIDEDPVFITTCTEREL
Pgbd5-Hs_NP_001   114 QTTHGLYKVQPFL--------DSLQNSFDSAFRPSQTQVLHEPLIDEDPVFIATCTEREL
Pgbd5-Gga_XP_01   114 QTTHGLYKIQPFL--------DSLQNGFDSAFRPSQAQVLHEPLIDEDPVFIATCTEREL
Tpb1_TTHERM_000   124 VHVDDHTKFKQFQ--------SILNRNYQQFYVPSNYLAIDEGIIP----FKGKTKFKVY
Tpb6_DAA80465.1   135 LKKDEIGKIKELQ--------NLLNQNFSKYYKHSNFLAIDEGVIP----FKGKSHLKVY
Tpb2_TTHERM_011   126 IRSDPIGKVRQYM--------DYLNENFKKYYYPGEFLAIDEGMIP----FNGKVAFKVY
Pgm-Pcau_PCAUDP   161 KGRDPIWKIRDFL--------NQMNTKFAKYYYPGEFITIDEGMIP----FAGKVQFKVY
Pgm-Pbi_PBIGNP2   161 KGRDPIWKIRDFL--------NQMNMRFAKYYYPGEFITIDEGMIP----FAGKVQFKVY
Pgm-Psex_PSEXPN   161 KGRDPIWKIRDFL--------NQMNMRFAKYYYPGEFITIDEGMIP----FAGKVQFKVY
Pgm-Ptet_PTET.5   161 KGRDPIWKIRDFL--------NQMNMRFAKYYYPGEFITIDEGMIP----FAGKVQFKVY
Pgbd3-Hs_NP_736   109 DPVDKFSKLRPLI--------SKLNERCMKFVPNETYFSFDEFMVP----YFGRHGCKQF
Pgbd1-Hs_NP_001   116 DQKDKFTKLRPLI--------KQMNKNFLLYAPLEEYYCFDKSMCE-------CFDSDQF
Pgbd2-Hs_NP_733   110 DASDRFAKVRPLI--------IRMNCNFQKHAPLEEFYSFGESMCE----YFGHRGSKQL
Bmo_BAD11135      126 KQTDNMAAFRSIF--------DQFVQCCQNAYSPSEFLTIDEMLLS----FRGRCLFRVY
Ago_ADU04477      121 RVIDKLSPIRTTF--------DLFLKNINKNYNLSEYTTIDEMLHP----FRGRCQWIQY
Hvi_ABD76335      119 LKTDKLAAVREFT--------DLMNNNFINNYCASENVTLDEQLPA----FRGRFSGVVY
Pgo_ADB45159      119 QRDDRFAPIRDLW--------QALISNFQKWYTPGSYITVDEQLVG----FRGRCSFRMY
Tni_AAA87375      117 RENDVFTPVRKIW--------DLFIHQCIQNYTPGAHLTIDEQLLG----FRGRCPFRMY
Cag_ADV17598      118 RQEDAFTPVRKIW--------EIFINQCRLNYVPGTNLTVDEQLLG----FRGRCPFRMY
Aip_ADV17599      119 RSDDAFIPVRKLW--------EIFINQCRLNYVPGGNLTVDEQLLG----FRGRCPFRMY
Har_ABS18391      119 RSDDAFLPVRKIW--------EIFINQCRQNHVPGSNLTVDEQLLG----FRGRCPFRMY
Hma_XP_00421184   114 RFEDKFAPLRNIM--------EMFITKCKSNCNPSAYLTVDEQLVT----FRGRCPFKMF
Tru_Pigibaku1_X   114 RQRDKLGAIRTVW--------DKWVRRLPLLYNPGPNVTIDGQLMP----FRGHCPFRQY
Oni_XP_00545891   112 RRVDKLAPFRKVW--------NMWTHRLEMLFSPDRDLCVDEQLVS----FKGRCSFRQY
Pny_XP_00575514   112 RHTDKLAPFHEVW--------DMWTHRLMMLFSPDRDLTVDEQLVP----FKARCSFRQY
Mlu_Mitra         118 NESDRLCKVRPVL--------DYFVPKFINIYKPHQQLSLDEGIVP----WRGRLFFRVY
PLE-wu_ref        115 PEYDKLFKIRPLL--------TLINTSFQENAHNSSSQSIDESMIL----FKGRSTLKQY
Aca_XP_00509140   121 ENYDPLFKLRPLL--------DTVRTACGSSYKPGRNISIDEAMIG----FNGRLHFKQY
Ami_ACT79641      122 DGYDRLYKVRPIL--------SHFNAKIQEIYKPGKNISVDEGMIG----FKGRLSFRQY
Nap_EQB62075_PG   141 RTEDRLYKIRNIF--------ETIVDSFKSSIRPGKDIVIDESMVP----WRGRLRFRQY
Goc_XP_00374069   122 NASGRSSKFIRVL--------EALQDKFCLAYTPGDMLVVDETMVP----FRGRLSFRQY
Mro_XP_00370823   118 VANNRLSKIQFII--------DELNTNFQKYYDPPELLCIDESLIP----FRGRIVFRQY
Xbo_BAF82021_Ur   141 PGHDRLYKLRPLI--------DSLSQRFAEVYTPSQNICVDESLLL----FKGRLKFRQY
Mfa_EHH62949_PG   128 KAHISLQKIKPVF--------DFLVNKFSTVYTPNRNIAVDESLML----FKGPLAMKQY
Ggo_XP_00405596   128 KAQISLQKIKPVF--------DFLVNKFSTVYTPNRNIAVDESLML----FKGPLAMKQY
Pgbd4-Hs_NP_689   128 KAQISLQKIKPVF--------DFLVNKFSTVYTPNRNIAVDESLML----FKGPLAMKQY
Sbo_XP_00393580   128 KAQISLQKIKPVF--------DFLVNKFSTVYTPNRNIAVDESLML----FKGPLAIKQY
consensus         301    d v kir v        e ln kfr ly p   l iDe ii     f gk   rly
```

```
PgmL2b-Psex_PSE  164 EAQDLQGRSIECILFMDVQTQYIIAIRFCYRENTTN--------------------TIFN
PgmL2a-Psex_PSE  173 EAQDLQGRSIECILLMDVQTQYIIAIRFCY-----RENISN---------------TIFN
PgmL2-Ptet_PTET  173 EAQDLQGRSIECILLMDVQTQYIIAIRFCFRENVSN--------------------TIFN
PgmL2-Pbi_PBIGN  173 EAQDLQGRSIECILLMDVQTQYIIAIRFCFRENISN--------------------TIFN
PgmL1-Pcau_PCAU  161 ----------QQIILCDAQNKLILWSHFCE---------TTENFTNL---------EIVQ
PgmL1-Psex_PSEX  156 NAQQ--------IILCDPKNKLIVWQYFCN--------NIQEEKVGL---------QIVY
PgmL1-Ptet_PTET  156 NSQQ--------IMICDPKSKLIVWQYFCNNIQEEKVGL-----------------QIVH
PgmL1-Pbi_PBIGN  156 HSQQ--------IMICDPKSKLIVWQYFCN--------NIQDEKVGL---------QIVY
Lia5_TTHERM_006  149 HNNSVYTQ----IFLCELSSAFVFGYFVVKDMSSFAN-------------------QICI
PgmL5a-Ptet_PTE  230 YSTK-----IQWYQLIDKESGIILQQFFWATPINKALDLNSHRDLQK---------RLRM
PgmL5a-Pbi_PBIG  228 YSTK-----IQWYQLIDKESGIILQQFFWATPINKALDLNSHRDLQK---------RLRM
PgmL5b-Ptet_PTE  230 STK------IQWYQLIDKESGIILQQFFWATPLSKALDLNNHRDLQK---------RLRM
PgmL5b-Pbi_PBIG  229 STK------IQWYQLIDKESGIILQQFFWATPINKALDLNNHRDLQK---------RLRM
PgmL5b-Psex_PSE  229 STK------IQWYQLIDKESGIILQQFFWATPINKTLDLNNHRDLQK---------RLRM
PgmL5a-Psex_PSE  229 STK------IQWYQLIDKESGIILQQFFWATPMNKTLDLNNHRDLQK---------RLRM
PgmL3c-Psex_PSE  161 ------------YYLFDFYKLFVIDLIIVS------HRVKQEDRIN----------RLMK
PgmL3c-Ptet_PTE  159 ------------YYLFDFSRLIIIDLIVVS------DRIQQEDRIN----------RLMR
PgmL3c-Pbi_PBIG  161 ------------YYLFEFQRLFIIDLIVVS------HRIHFEDRIN----------RLMR
PgmL3-Pcau_PCAU  161 ------------YYLFDCESSQILDLLVVV-------HGNEEEKLN----------KIIR
PgmL3a-Pbi_PBIG  150 ----QNQKAYKTYYIFDYDSSQIIDLLVVC------NNVKNEDRIN----------RVMR
PgmL3a-Ptet_PTE  150 ----QNQKAYKAYYIFDYDSSQIIDLLVVC------NNVKNEDRIN----------RVMR
PgmL3b-Ptet_PTE  151 ----QNQKAYNAYYIFDYDSSQIIDLLVVCNNVKNEDRIN----------------RVMR
PgmL3b-Pbi_PBIG  151 ----QNQKVYKAYYIFDYDSSQIIDLLVVC------NNVKNEDRIN----------RVMR
PgmL3a-Psex_PSE  153 ----QNQKAYKAYYIFDYDSSQIIDLLVVC------NNVKTEDRIN----------RVMR
PgmL4b-Psex_PSE  298 ------------ISLSDGMKGYILDFIYGIKDQ-----------------------KIIQ
PgmL4a-Psex_PSE  300 ------------ISLSDGMKGYVLDFIYGIKDQ-----------------------KIIQ
PgmL4b-Ptet_PTE  298 ------------ISLSDGMKGYVLDFIYGIKDQ-----------------------KILQ
PgmL4b-Pbi_PBIG  299 ------------ISLSDGMKGYVLDFIYGIKDQ-----------------------KILQ
PgmL4a-Ptet_PTE  298 ------------ISLSDGMKGYVLDFIYGI-----KEQ------------------KILQ
PgmL4a-Pbi_PBIG  298 ------------ISLSDGMKGYVLDFIYGIKDQ-----------------------KIIH
Tpb7_TTHERM_006  157 FKSN-SQYFLDEFRLYDYSNSFIVDVQFDKSTPELKKSQDYVQKLMR---------VQAN
Pgbd5-Rty_XP_02  166 RKRKKRKFSL--WVRQCAATGYICQVYVNLKEGTASDGLDTLKNRPQLHSL-----VAKS
Pgbd5-Dre_NP_00  167 RKRKKRKFSL--WVRQCSSTGFICQIYVHLKEGPGPDGLDTLKNKPQLHSL-----VAKQ
Pgbd5-Hs_NP_001  166 RKRKKRKFSL--WVRQCSSTGFIIQIYVHLKEGGGPDGLDALKNKPQLHSM-----VARS
Pgbd5-Gga_XP_01  166 RKRKKRKFSL--WVRQCSSTGFICQIYVHLKEGSGADGLDALKNKPQLHSM-----VAKS
Tpb1_TTHERM_000  172 CPQKPVKFGIKEYLFCDY-SGYTLNLIIHSPHEKQNIRDIYQPQTLQTQD------IVNE
Tpb6_DAA80465.1  183 CPGKPYKYGIKEFLLCDF-TGYTILQEIATKKDKQNVYTKNSKDIKNYTHL-----LVKQ
Tpb2_TTHERM_011  174 NPDKPDKFGIKEYVCCDSQNAYTLESQLYYGNHENEEFPEITLSKTNE--------VVMN
Pgm-Pcau_PCAUDP  209 NPDKPTKWGIKEYLLCDASNTYTFQLRLYHGQTMWNNDFKQTMFVNEEDTQHRTMELVLQ
Pgm-Pbi_PBIGNP2  209 NPDKPTKWGIKEYLLCDASNTYTFQLRLYHGQTMWNNDFKQTMFVNEEDTQHRTMELVLQ
Pgm-Psex_PSEXPN  209 NPDKPTKWGIKEYLLCDASNTYTFQLRLYHGQTMWNNDFKQTMFVNEEDTQHRTMELVLQ
Pgm-Ptet_PTET.5  209 NPDKPTKWGIKEYLLCDASNTYTFQLRLYHGQTMWNNDFKQTMFVNEEDTQHRTMELVLQ
Pgbd3-Hs_NP_736  157 IRGKPIRFGYKFWCGATC-LGYICWFQPYQGKNPNTKHEEYGVGAS----------LVLQ
Pgbd1-Hs_NP_001  161 LNGKPIRIGYKIWC-GTTTQGYLVWFEPYQEESTMKVDEDPDLGLGGN--------LVMN
Pgbd2-Hs_NP_733  158 HRGKPVRLGYKIWC-GTTSRGYLVWFEPSQGTLFTKPDRSLDLGGS----------MVIK
Bmo_BAD11135     174 IPNKPAKYGIKILALVDAKNFYVVNLEVYAGKQPSGPYAVSNRPFE----------VVER
Ago_ADU04477     169 IPSKPAKYGIKMFALCDAKTFYTSKIEIYVGKQPPGPYEVSNSPID----------IVKR
Hvi_ABD76335     167 MPNKPTKYGIKHYALVDSATFYLLKFEIYAGVQPEGPYRMPNDTVS----------LVKR
Pgo_ADB45159     167 IPNKPNKYGIKLVMAADVNSKYIVNAIPYLGKGTDPQNQPLATF------------FIKE
Tni_AAA87375     165 IPNKPSKYGIKILMMCDSGTKYMINGMPYLGRGTQTNGVPLGEY------------YVKE
Cag_ADV17598     166 IPNKPDKYGIKFPMVCDAATKYMVDAIPYLGKSTKTQGLPLGEF------------YVKE
Aip_ADV17599     167 IPNKPDKYGIRFPMMCDAATKYMIDAIPYLGKSTKTNGLPLGEF------------YVKE
Har_ABS18391     167 IPNKPDKYGIKFPMMCAAATKYMIDAIPYLGKSTKTNGLPLGEF------------YVKD
Hma_XP_00421184  162 IPTKPGKNGMKIWILCDSETSYCINLQPYIGRVNGERDVGQGTR------------VILE
Tru_Pigibaku1_X  162 IPSKPAKYGIRIWAACDATSSYAWNLQVSTGK---PDGGAPEKNQGMR--------VVLD
Oni_XP_00545891  160 MPKKPAKYGIKIWAACDVKTSYAWRLQVYTGKPAPDRVEVNQGMR-----------VVLD
Pny_XP_00575514  160 IPKKPAKYGIKVWATCDAKTSYAWRLHVYTGRAAGERAEVNQGMR-----------VVLQ
Mlu_Mitra        166 NAGKIVKYGILVRLLCESDTGYICNMEIYCGEGKRLLE------------------TIQT
PLE-wu_ref       163 MPMKPIKRGFKVWCRCDSITGYLYEFDIYTGRDGDRVEDNLGGK------------VVKK
Aca_XP_00509140  169 IRNKPTKWGIKVWCVAEAETGYMLNFRFYTGKINEPMTDGVGHH------------VVMN
Ami_ACT79641     170 MPAKPTKYGIKVWMAADASNGFVINHEVYLGKQRGRVLANGLGYS-----------VVME
Nap_EQB62075_PG  189 IPGKRHKYGVKLYKLC-LPDGYTYNIEIYAGKNNTIIKKSHSHD------------VVMR
Goc_XP_00374069  170 IPGKSHKYGIKIFKLCN-KDGYTFRTKVYTGAEGVRSRALPSD-------------IVIE
Mro_XP_00370823  166 LKQKKHKYGIKIFKLCCS-HGYTYNCHVYTGKALDRENTTPSN-------------VVMS
Xbo_BAF82021_Ur  189 IPSKRSRYGMKFYKLCESSTGYTRSFMFYEGKDTNLDPPGCPLDLTASGK------IVWE
Mfa_EHH62949_PG  176 IPTKRVRFGLKLYVLCESQSGYVWNALVHTGPGMNLKDSADGLKSSR---------IVLT
Ggo_XP_00405596  176 LPTKRVRFGLKLYVLCESQSGYVWNALVHTGPGMNLKDSADGLKSSR---------IVLT
Pgbd4-Hs_NP_689  176 LPTKRVRFGLKLYVLCESQSGYVWNALVHTGPGMNLKDSADGLKSSR---------IVLT
Sbo_XP_00393580  176 IPTKRVRFGLKLYVLCESQSGYVWNALVHTGPGMNLKDSADGLKSSR---------IVLT
consensus        361    k   kygik yllcd  tgyii i vw g                        vvv
```

```
PgmL2b-Psex_PSE   204 MLDPYKHKN---H-KLYFQQ--ELLSLE----------QVYSCGILAQ-----------S
PgmL2a-Psex_PSE   213 MLDPYKHKN---H-KLYFQQ--ELLSLEQVQILVEF-FAIYSCGILAQ-----------S
PgmL2-Ptet_PTET   213 MLDPYKHKN---H-KLYFQQ--ELLTLEQVQILVEF-FAIYSCGILAS-----------T
PgmL2-Pbi_PBIGN   213 MLDPYKHKN---H-KLYFQQ--ELLTLEQVQILVEF-FAIYSCGILAS-----------T
PgmL1-Pcau_PCAU   193 LLAHFKDKN---H-ILYCYD--NILTVEQIIFLKLE-YKIQCVGSIKQ------------
PgmL1-Psex_PSEX   191 VLQQFKDNN---H-TLYVYD--NILNMEQICFLKTK-LKIQCVAMVDS-----------L
PgmL1-Ptet_PTET   191 VLQQFKDNN---H-TLYIYD--NILNLEQIAYLKIK-LKIQCVQKLDS-----------I
PgmL1-Pbi_PBIGN   191 LLQQFKDNN---H-TLYIYD--NILNLEQIAFLKIK-LKIQCIQKLDN-----------M
Lia5_TTHERM_006   186 NLESFTNQN---H-HVYFQNEEFFSNYEKIQELLNS--KIHISSFLNNKF---DQFPQSL
PgmL5a-Ptet_PTE   276 MFEPYHLGR---H-IVYSQ---GLLNAESVLQLYQQ--KIFVCTDL-------------M
PgmL5a-Pbi_PBIG   274 MFEPYHLGR---H-IVYSQ---GLLNAESVLQLYQQ--KIFVCTDL-------------M
PgmL5b-Ptet_PTE   275 MFEPYHLGR---H-IVYSQ---GLLNAESILQLYQQ--KIFVCTDL-------------V
PgmL5b-Pbi_PBIG   274 MFEPYHLGR---H-IVYSQ---GLLNAESILQLYQQ--KIFVCTDL-------------M
PgmL5b-Psex_PSE   274 MFEPYHLGR---H-IVYSQ---GLLNAESVLQLYQQ--KIFVCTDLNQ------------
PgmL5a-Psex_PSE   274 MFEPYHLGR---H-IVYSQ---GLLNAESVLQLYQQ--KIFVCTDL-------------V
PgmL3c-Psex_PSE   193 MLFKYSNHN---H-VLYIM---FELSLERIIQLTDQ--EIFPVISYKK-----------T
PgmL3c-Ptet_PTE   191 MLFKYSNHN---H-ILYVM---FELSLERIIQLTDQ--DIYPVITYEK-----------R
PgmL3c-Pbi_PBIG   193 MLFRYSNRN---H-ILYVM---FELNLERIIQLTDQ--NIYPVISYQK-----------I
PgmL3-Pcau_PCAU   192 MLLKYANQN---H-VMYFM---FEIKLEKIIQLVDQ--GIYPVIYYKN-----------L
PgmL3a-Pbi_PBIG   190 MLYKYSNQN---H-VIYIM---FDLSLEKIIQLVDQ--SIYPVIRYQK-----------I
PgmL3a-Ptet_PTE   190 MLYKYSNQN---H-VTYIL---FDLSLDKIIQLVDQ--SIYPVIRYQN-----------I
PgmL3b-Ptet_PTE   191 MLYKYSNQN---H-VTYIM---FYLSLERIIQLVDQ--SIFPVIRYKN-----------I
PgmL3b-Pbi_PBIG   191 MLYKYSNQN---H-VTYIM---FDLSLERIIQLVDQ--SIYPVIRYQN-----------I
PgmL3a-Psex_PSE   193 MLYKYSNQN---H-VTYIM---FDMSLERIIQLVDQ--SIYPVIRYQN-----------V
PgmL4b-Psex_PSE   323 SLQKYQGKH---H-KLYLGP--EVSSLNLINQLKKK--QFGSLAKVVDKQ---TQLTYDQ
PgmL4a-Psex_PSE   325 SLQKYQGKH---H-KLYLGP--EVSSLNLISQLKKK--QFGSLAKVVDKQ---TQLTQDQ
PgmL4b-Ptet_PTE   323 SLQKYQGKH---H-KLYLGP--DVSSLNLISQLKKK--QFGSLAKLVDKQ---TQLTQEQ
PgmL4b-Pbi_PBIG   324 SLQKYQGKH---H-KLYLGP--DVSSLNLISQLKKK--QFGSLAKLVDKQ---TQLSQEQ
PgmL4a-Ptet_PTE   323 SLQIYQGKH---H-KLYLGP--DVSSLNLIIQLKKK--QFGALAKVVDKQ---TQLTQDQ
PgmL4a-Pbi_PBIG   323 SLQNYQGKH---H-KLYLGP--DVSSLNLITQLKKK--QFGSLAKVVDKQ---TQLTQDQ
Tpb7_TTHERM_006   207 VLDNYKGKG---H-KVVIQN--QLFDADILRSLRHQ--NIGVVTRSHIEN---IQFTPKQ
Pgbd5-Rty_XP_02   219 LCQNLAGKN---Y-IIYTGP--SITSLNLFEEFEKK--GIYCCGLLSSRKSDCTGLPQSM
Pgbd5-Dre_NP_00   220 LCQNLAGRN---A-IIFTGP--SITSLNLFQEFEKQ--GIYCCGLLSIRKSDCTGLPQSM
Pgbd5-Hs_NP_001   219 LCRNAAGKN---Y-IIFTGP--SITSLTLFEEFEKQ--GIYCCGLLRARKSDCTGLPLSM
Pgbd5-Gga_XP_01   219 LCQNASGKN---Y-IIFTGP--SITSLNLFEEFEKQ--EIYCCGLLSARKSDCTGLPPSM
Tpb1_TTHERM_000   225 LIKDYQPLS---GSILIMDN--YYNSLNLIHDLNQQ--NIGVLGTVRPDRMNFTEEQKKM
Tpb6_DAA80465.1   237 LIHGQNNLQ---NCIIFMDN--FYTSISLFEELSQN--KIGAVGTVRLDR---LKLNKEQ
Tpb2_TTHERM_011   226 LLKDYEGRF---H-KVVMDN--YYNSPTLFYLMKQK--SFGALGTMRIGR---LKLPPYL
Pgm-Pcau_PCAUDP   269 MCKDYEHKA---H-KVVMDN--YYSSWQLFRELRNR--GIGAVGTIRHNR---TGLTKKD
Pgm-Pbi_PBIGNP2   269 MCKDYEHKA---H-KVVMDN--YYSSWMLFRELRNR--GIGAVGTIRHNR---TGLTKKD
Pgm-Psex_PSEXPN   269 MCKDYEHKA---H-KVVMDN--YYSSWMLFRELRNR--GIGAVGTIRHNR---TGLTKKD
Pgm-Ptet_PTET.5   269 MCKDYEHKA---H-KVVMDN--YYSSWMLFRELRNR--GIGAVGTIRHNR---TGLTKKD
Pgbd3-Hs_NP_736   206 FSEALTEAHPGQY-HFVFNN--FFTSIALLDKLSSM--GHQATGTVRKDH---IDRVPLE
Pgbd1-Hs_NP_001   212 FADVLLERGQYPY-HLCFDS--FFTSVKLLSALKKK--GVRATGTIRENR---TEKCPLM
Pgbd2-Hs_NP_733   207 FVDALQERGFLPY-HIFFDK--VFTSVKLMSILRKK--GVKATGTVREYR---TERCPLK
Bmo_BAD11135      224 LIQPVARSH---R-NVTFDN--WFTGYELMLHLLNE-YRLTSVGTVRKNK---RQIPESF
Ago_ADU04477      219 LVTPIENSK---R-NLTTDN--WYTSIPLADYLLQK--KITLTGTLKKNK---REIPTEF
Hvi_ABD76335      217 MTEPIWGTG---R-NVTMDN--WFTSVPLANILLKD-HQLTMVGTIRKNK---PEIPTCF
Pgo_ADB45159      215 ITSTLHGTN---R-NITMDN--WFTSVPLANELLMAPYNLTLVGTLRSNK---REIPEKL
Tni_AAA87375      213 LSKPVHGSC---R-NITCDN--WFTSIPLAKNLLQEPYKLTIVGTVRSNK---REIPEVL
Cag_ADV17598      214 LTQTVHGTN---R-NVTCDN--WFTSVPLAKSLLNSPYNLTLVGTIRSNK---REIPEEV
Aip_ADV17599      215 LTKTVHGTN---R-NVTCDN--WFTSIPLAKNMLQAPYNLTIVGTIRSNK---REIPEEI
Har_ABS18391      215 LTKTVHGTN---R-NITCDN--WFTSIPLAKNMLQAPYNLTIVGTIRSNK---REMPEEI
Hma_XP_00421184   210 LADHLNGSG---R-HITADK--FFSNIHLARALLGR--KMTYTGTIKKNK---EEIPKKL
Tru_Pigibaku1_X   211 MSQGLSG-----H-NITCDS--FFTSHKLGQELLKR--KLTIVGTIRKNR---SELPPQL
Tpb7_TTHERM_006    93 MTEGLQG-----H-VVTCDN--FFTSCALADELLKR--KMALVGTIRQNK---PELPPHL
Pny_XP_00575514   209 MTEGLQG-----H-VATCDN--FFTSFALAEELLKR--QIALVGTIRQNE---PEISPVL
Mlu_Mitra         208 VVSPYTDSW---Y-HIYMDN--YYNSVANCEALMKN--KFRICGTIRKNR----GIPKDF
PLE-wu_ref        211 LTEKLKGMA---AVHVTFDN--FFCSYDIMNYLHVN--GISASGTVRRQR---ADLPKLV
Aca_XP_00509140   217 CAADYLGKY---H-CIYFDN--YFSSVRLAEDLLKK--KTYSCATVRTNR-------KGW
Ami_ACT79641      219 LMNPFLNKN---H-HVYFDN--FFSSPKLLEDLQNE--GTYACSTVRAGR---VGLPPSS
Nap_EQB62075_PG   236 LLNGLLFEG---R-ILFTDG--YYTSVPLGEELLQN--NTFICGTVKINK-------KFL
Goc_XP_00374069   216 LSEPFLDDG---W-TLVTDN--YFTSVPLAKELLGR--HTNLLGTLRRHR-------KFL
Mro_XP_00370823   212 LLKNLFHRG---H-TLCTDN--WYTSIDLANRLIEK--NTHLIGTLRTNR---RGNPSEV
Xbo_BAF82021_Ur   243 LITPLLGQG---Y-HLYVDN--FYTSVPLFRTLYWL--DTPACGTVRQNR---KGLPKEL
Mfa_EHH62949_PG   227 LVNDLLGQG---Y-CVFLDN--FNISPMLFRELHQN--RTDAVGTARLNR-------KQI
Ggo_XP_00405596   227 LVNDLLGQG---Y-CVFLDN--FNISPMLFRELHQN--RTDAVGTARLNR-------KQI
Pgbd4-Hs_NP_689   227 LVNDLLGQG---Y-CVFLDN--FNISPMLFRELHQN--RTDAVGTARLNR-------KQI
Sbo_XP_00393580   227 LVNDLLGQG---Y-CVFLDN--FNISPMLFRELHQN--RTDAVGTARLNR-------KQI
consensus         421 ml  y       h  lyvDn  ffssl lv  l       i  vgtlr  k     l   l
```

```
PgmL2b-Psex_PSE   237 IQTQKIDFKDRLVTNNHVMSLKL----LIPNQADFKVFA-----STADGFH---------
PgmL2a-Psex_PSE   255 TQTQKIDFKDGLVTNNHVMLLKL----LTPNQADFKVFA-----STADGFH---------
PgmL2-Ptet_PTET   255 TQTQKIDFKDGLVTNNHVMLLKL----LTPNQADFKVFA-----STADGFH---------
PgmL2-Pbi_PBIGN   255 TQTQNIDFKDGLVTNNHVMLLKL----LTPNQADFKVFA-----STADGFH---------
PgmL1-Pcau_PCAU   234 -------VVNTPYHNSVI---------SIINWQNETIAI--------------------
PgmL1-Psex_PSEX   233 SASQLPQIPLYLDFMSIVKEQNE----IYAVTSGEQIQQ--------------------
PgmL1-Ptet_PTET   233 QASQVPQIPLYLDYMSIIKEQNE----VFAVTSGEQIQQ--------------------
PgmL1-Pbi_PBIGN   233 ISSQLPQIPLYLDYMSIIKEQNE----VFAVTSGDQIQQ--------------------
Lia5_TTHERM_006   237 QNEMLHNKPLKANNSETIFDRQTQTQLLIKSDENLKKEV-----FLTT-----------
PgmL5a-Ptet_PTE   314 QHSLLPHPPTGDQQVVYSSKNKEP---IYLCYKNQQWIT-----CVSLQ----------
PgmL5a-Pbi_PBIG   312 QHSLLPHPPTGDQQVFYSSKNKEP---IYLCYKNQQWIT-----CVSLQ----------
PgmL5b-Ptet_PTE   313 QHSLLPHPPTGDQQVLYSSKSKEP---IYLCYKNQQWST-----CVSLH----------
PgmL5b-Pbi_PBIG   312 QHSLLPHPPTSDQQVFYSSKSKEP---IYLCYKNQQWST-----CVSLP----------
PgmL5b-Psex_PSE   313 -HSLLPHPPNADQQIFYSSKSKEP---IYLCYKNQQWNS-----CVSLQ----------
PgmL5a-Psex_PSE   312 QHSLLPHPPTSDQQIFYSSKSKEP---IYLCYKNQQWIT-----CVSLQ----------
PgmL3c-Psex_PSE   233 QHELFQNLQSGQYQIEQLYLIKN----QYQVHLFPQKQL------ISNKHFLS-------
PgmL3c-Ptet_PTE   231 LHQTIQNLLPGEYQIEQLYLIKN----QYQVHLFPQKQS------ISNQSFLK-------
PgmL3c-Pbi_PBIG   233 QHLTIQNLLPGEYQIEQLYLIKN----QHQAHLFPKKQL------ISNKSFLK-------
PgmL3-Pcau_PCAU   232 QHSLIQTLNDGEYSLDQLYLIKQ----GQEIYIFPQKRL------ISHKHYV--------
PgmL3a-Pbi_PBIG   230 NHNLIQMLQDGEYELDQLFLIRQ----GIQVDVFPQRRL------ISNRHYMT-------
PgmL3a-Ptet_PTE   230 SHNLIQMLQDGEYELDQLFLVKQ----GTQVDVFPQRRL------ISNRPYMT-------
PgmL3b-Ptet_PTE   231 THNLIQMLQDGDYQLDQLFLVKQ----GTQVDVFPQRRL------ISSRHYM--------
PgmL3b-Pbi_PBIG   231 NHNLIQMLQDGDYELDQLFLIKQ----GTQVDVFPQRRL------ISSRHYMT-------
PgmL3a-Psex_PSE   233 NHYLIQMLQDGDYELDQLFLIKQ----GTQVDVFPQRRL------ISNRHYMT-------
PgmL4b-Psex_PSE   372 IQEVKQNAQKGKSTQLLSSDNQA----IMLIYAEKQQHLQQVEGFVSSFADFTRLKPQ--
PgmL4a-Psex_PSE   374 MQEVKQNAQKGQSTQFLSSDNQT----IMLIYAERQQHLQQVEGFVSSFADFTRLKPQ--
PgmL4b-Ptet_PTE   372 IQEVKQNAQKGKSTQFLSSDNQT----IMLIYAERQQHIQQVEGFVSSFADFTRLKPQ--
PgmL4b-Pbi_PBIG   373 IQEVKQNAQKGKSTQFLSSDNQT----IMLIYAERQQHLQQVEGFVSSFADFTRLKPQ--
PgmL4a-Ptet_PTE   372 IQEVKQNAQKGQSTQFLSSDNQT----IMLIFAERQQHLQQVEGFVSSFADFTRLKPQ--
PgmL4a-Pbi_PBIG   372 IQEVKQNAQKGQSTQFLSSDNQN----IMLIYAERQQHLQQIEGFVSSFADFTRLKPQ--
Tpb7_TTHERM_006   256 LKIISENKLFKNFFFIYEREVQA----VITPYSFSHKYA-----FISNFL---------
Pgbd5-Rty_XP_02   271 LVNPEIPQARGQYRVRMKGNM------SLISWSNKGQFY-----FLTNAYSPFKEGVI--
Pgbd5-Dre_NP_00   272 LINTEAPQQRGQSHVKMRGNM------SIINWYNKGNFR-----FLTNAYSPTKEGVI--
Pgbd5-Hs_NP_001   271 LTNPATPPARGQYQIKMKGNM------SLICWYNKGHFR-----FLTNAYSPVQQGVI--
Pgbd5-Gga_XP_01   271 LNNPDTPQSRGQYRIRMKGNM------SLICWYNKGHFR-----FLTNAYSPVQQGVI--
Tpb1_TTHERM_000   278 M--KIQNFNKGETKSLTKDNI------HIFLWRDKDKLV----KMITNFLDNRKIVKS--
Tpb6_DAA80465.1   287 KNEIKKDSLKQTGSISFMQKNMH----LLFWYDNNRAVK-----ILSNCIGMEQVKS---
Tpb2_TTHERM_011   275 L-SQIMEVHQNKVLSYIQGDINLA---IFFRGTNDKEIC-----LMSNFIDQKKVKDDFW
Pgm-Pcau_PCAUDP   318 LTSKHFQQIYNQYHYAYYMNQSNELMLMYFQGTSEKEIA-----LISNFLDNSLNESHMW
Pgm-Pbi_PBIGNP2   318 LTSKHFQQIYNQYHYAYYMNQSNELMLMYFQGTSEKEIA-----LISNFLDNSLNEQHMW
Pgm-Psex_PSEXPN   318 LTSKHFQQIYNQYHYAYYMNQSNELMLMYFQGTSEKEIA-----LISNFLDNSLNEQHMW
Pgm-Ptet_PTET.5   318 LTSKHFQQIYNQYHYAYYLNQSNELMLMYFQGTSEKEIA-----LISNFLDNSLNEQHMW
Pgbd3-Hs_NP_736   258 SDVALKKKERGTFDYRIDGKGN-----IVCRWNDNSVVT-----VASSGAGIHPLCLVSR
Pgbd1-Hs_NP_001   264 NVEHMKKMKRGYFDFRIEENNEI----ILCRWYGDGIIS-----LCSNAVGIEPVNEVSC
Pgbd2-Hs_NP_733   259 DPKELKKMKRGSFDYKVDESEEI----IVCRWHDSSVVN-----ICSNAVGIEPVRLTSR
Bmo_BAD11135      274 IRTDRQP---NSSVFGFQKDITL----VSYAPKKNKVVV-----VMSTMHHDNS------
Ago_ADU04477      268 LPHKKKEV--GSSVFGFQKNKTL----VSYVPRKNKAVI-----LLSTMHH---------
Hvi_ABD76335      267 QPKRTRTEHSSLFGFQEDVTL------CSYVPKKSKAVL-----LISSMHND--------
Pgo_ADB45159      266 KNSKSRAI--GTSMFCYDGDKTL----VSYKAKSNKVVF-----ILSTIHD---------
Tni_AAA87375      264 KNSRSRPV--GTSMFCFDGPLTL----VSYKPKPAKMVY-----LLSSCDE---------
Cag_ADV17598      265 KNSRSRQV--GSSMFCFDGPLTL----VSYKPKPSKMVF-----LLSSCNE---------
Aip_ADV17599      266 KNSRSRPV--GSSMFCFDGPLTL----VSYKPKPSRMVF-----LLSSCDE---------
Har_ABS18391      266 KNSRSRPV--GSSMFCFDGPLTL----VSYKPKPSKMVF-----LLSSCDE---------
Hma_XP_00421184   259 LPALHRPVYSSIFGFQIDSTI------VSYVPKKNKAVI-----LLSTIHHS--------
Tru_Pigibaku1_X   258 LTSKNRPVKSSKFAYTADTSL------VSYVPKKGKNVV-----LMSTLHRD--------
Oni_XP_00545891   256 LQAKKRALFSSIFAFTKTRTL------VSYTARRGKNVL-----LLSTKHRR--------
Pny_XP_00575514   256 RQTKGRAIYSSTFAFTVRHTL------VSYIPRWGWNVL-----LLSTKHRR--------
Mlu_Mitra         256 QT---ISLKKGETKFIRKNDI------LLQVWQSKKPVY-----LISSIHSAEMEESQNI
PLE-wu_ref        261 KSKKKLKLTKGQYKWRVKENV------AFVIWQDTKEVL-----FMTNAFHPKDNETSLP
Aca_XP_00509140   262 PFPTKNKQKKGTLNMKQRGTM------VATQWHDKRPVN-----VLSTCCNPTTTDVTRR
Ami_ACT79641      268 RRKLKRE---GEMICEQKGNL------VYTKWHDKRDVN-----ILSTNFDPLEPKTVKE
Nap_EQB62075_PG   281 PPQAKQKQKRGEL-MHFENRSGV----KFLKWTDKRPVC-----MLTTSKNHRCK-----
Goc_XP_00374069   261 PQNVVTARLDRNQLTGRISRDGI----VVGKWKDKRDVL-----FLSTKHDLRMASSGRR
Mro_XP_00370823   261 IKT---NLKRGQV-IAKENNQGI----TVLKWKDKRDVL-----VLSTKHSNEMIN----
Xbo_BAF82021_Ur   292 VQ---KKLKRGEVHALRSDEL------LALKFADTKPVC-----MLTTIHNESVVVQ---
Mfa_EHH62949_PG   272 PNDLKKRIAKGTTVARFCGEL------MALKWCDEKEVT-----MLSTFHNDTMIEV---
Ggo_XP_00405596   272 PNDLKKRIAKGTTVARFCGEL------MALKWCDGKEVT-----MLSTFHNDTVIEV---
Pgbd4-Hs_NP_689   272 PNDLKKRIAKGTTVARFCGEL------MALKWCDGKEVT-----MLSTFHNDTVIEV---
Sbo_XP_00393580   272 PNDLKKRIAKGTTVARFCGEL------MALKWCDTKEVT-----MLSTFHSDTVIEV---
consensus         481        v g         l      l   w   k i      mlst
```

```
PgmL2b-Psex_PSE  279 --------RNTSQDKKSYAVVIYQEYIKLV-EQFPALSDAYKCLFS-SATID-GAIYTKC
PgmL2a-Psex_PSE  297 --------RNTSQDKKSYAVVIYQEYIKLV-EQFPALSDAYKCLFS-SATVN-GAIYTEM
PgmL2-Ptet_PTET  297 --------RNTSQDKKSYAVVIYQEYIKLV-EQYPALSDAYKCLFS-SATVN-GAIYTEM
PgmL2-Pbi_PBIGN  297 --------RNTSQDKKSYAVVIYQEYIKLV-EQYPALSDAYKCLFS-SATVN-GAIYTEM
PgmL1-Pcau_PCAU  257 ----------TAGDNLEEILVNQQNFRERM-LYYS--IHKNRPLFS-YSSYS-SGIMTEL
PgmL1-Psex_PSEX  268 ------------------LICIYNQYKESM-LLYC--IHKFRPNFS-YLHFS-SAVMTEL
PgmL1-Ptet_PTET  268 ------------------LICTYNQYKETM-LLYC--IHKFRPNFS-YIHYS-SAIMTEL
PgmL1-Pbi_PBIGN  268 ------------------LICTYNQYKESM-LLYC--IHKFRPNFS-YLHYS-SAVMTEL
Lia5_TTHERM_006  280 ----------SGTVKQDKMVEKHKQAIQKITQKLRMLLTEYRFVSINNDT---TSIFEEL
PgmL5a-Ptet_PTE  355 ------SLQMRSQYLQELSQLQFTEEKIAI-QPSEPENTSINSNS--------KIIFIFV
PgmL5a-Pbi_PBIG  353 ------SLQQRNQYLQELSQMQFTEEKIAI-QPSEPENTSINSNS--------KIIFIFV
PgmL5b-Ptet_PTE  354 ------SLQQRNQYLQELSQMQFAEEKIAI-QPSEPENASINSTS--------KIIFIFV
PgmL5b-Pbi_PBIG  353 ------SLQQRFQYLQELSHMQFAEEKIAI-QPSEPENASINSTS--------KIIFIFV
PgmL5b-Psex_PSE  353 ------SLQSRNLYLQEISQQQFTEEKIAI-QPSEPENTSINSTQ--------KIIFIFV
PgmL5a-Psex_PSE  353 ------SLQQRNQYLQELSQTQFTEEKIAI-QPSEPENTSINSTS--------KIIFIFV
PgmL3c-Psex_PSE  276 ------VYQEIQQQYNDNQIMNLLQYRSNF-YLCSQLNSSE------GFNQL-RIEFEEY
PgmL3c-Ptet_PTE  274 ------VYKDVQQQYYEHSIMNLLQYRSNF-YLCSQLNSVE------GFNQL-RIEFEEY
PgmL3c-Pbi_PBIG  276 ------VYKDVQQQYNENPIMNLLQYRSNF-YLCSQLNNTE------GFNQF-RIEFEEY
PgmL3-Pcau_PCAU  274 ------TNHRSIQEEVSEHLLNIQQFRSQF-YFSSQLNSPE------GFQQL-RIEFEEY
PgmL3a-Pbi_PBIG  273 ------SYEKIKNQLQEH-IINLQQFRSNF-YQSSQLNNTE------GFHQL-RVEFEEY
PgmL3a-Ptet_PTE  273 ------SYEKIKEQLQEH-ITNLQQFRSNF-YQSSQLNNTE------GFHQL-RVEFEEY
PgmL3b-Ptet_PTE  273 ------TSYEKIQEQLQEHIINLQQFRSNF-YQSSQLNNTESFHQL-------RIEFEEY
PgmL3b-Pbi_PBIG  274 ------SYEKIKEQLQEH-IINLQQFRSNF-YQSSQLNNTE------QFHQL-RVEFEEY
PgmL3a-Psex_PSE  276 ------SYEKIKEQLQEH-ITNLQQYRSNF-YQSSQLNNTE------GFHQL-RVEFEEY
PgmL4b-Psex_PSE  426 ------DTKIKSDINKPIILYLYDKIKTQY-DKRGKTYQYSQLPHQ-DANQH-LEILVQL
PgmL4a-Psex_PSE  428 ------DTKIKSDVNKPIILYLYDKIKTQY-DKRGKTYQYSQLPHQ-DANQH-LEILVQL
PgmL4b-Ptet_PTE  426 ------DTKIKSDVNKPIILYLYDKIKTQY-DKRGKTYQYSQIPHQ-DANQH-LEILVQL
PgmL4b-Pbi_PBIG  427 ------DTKIKSDVNKPIILYLYDKIKTQY-DKRGKTYQYSQIPHQ-DTNQH-LEILVQL
PgmL4a-Ptet_PTE  426 ------DTKIKSDVNKPIILYLYDKIKTQY-DKRGKTYQYSQIPHQ-NGNQH-LEILVQL
PgmL4a-Pbi_PBIG  426 ------DTKIKSDINKPIILYLYDKIKTQY-DKRGKTYQYSQLPHQ-SANQH-LEILVQL
Tpb7_TTHERM_006  297 ------GLEDTKDLANPKMQQIYSE-KGVV-LDYKDINPRDVE----GVKWD-QLVFLRV
Pgbd5-Rty_XP_02  318 ------IRRKSGEIRCPLAVEAFAAHLGYI-CKYDDKYSKYFISHKANKTW--QQVFWFV
Pgbd5-Dre_NP_00  319 ------IKRKSGEIPCPLAVEAFAAHLSYI-CKYDDKYSKYFIFHKPNKTW--QQVFWLT
Pgbd5-Hs_NP_001  318 ------IKRKSGEIPCPLAVEAFAAHLSYI-CRYDDKYSKYFISHKPNKTW--QQVFWFA
Pgbd5-Gga_XP_01  318 ------IKRKSGEIPCPLAVEAFAAHLSYI-CKYDDKYSKYFISHKPNKTW--QQVFWFA
Tpb1_TTHERM_000  324 ------MKKTGQIKTIPLMVDIYNKYAHSV-DKRNQICQNYRIHKR-SQKWW-KCVFYRL
Tpb6_DAA80465.1  335 ------QNIKNPIKDIPLMVKLYNKYSHSV-DKRNQLLQYYRTIRR-TRKWW-KTIFYRL
Tpb2_TTHERM_011  326 RVLAYVKEDRDIFKEQNLQSAYYNKYKGGV-DRRNSYLAAYRNCRK-NIKWY-RPVFYRM
Pgm-Pcau_PCAUDP  373 DISK--QHYYVPHLKAPYMMYVYNKYKGGV-DRRNSYVVKYRSRFP-AKKWW-QSVFERL
Pgm-Pbi_PBIGNP2  373 DISK--QHYYVPHLKAPYMMYVYNKYKGGV-DRRNSYVVKYRSRFP-AKKWW-QSVFERL
Pgm-Psex_PSEXPN  373 DISK--QHYYVPHLKAPYMMYVYNKYKGGV-DRRNSYVVKYRSRFP-AKKWW-QSVFERL
Pgm-Ptet_PTET.5  373 DISK--QHYYVPHLKAPYMMYVYNKYKGGV-DRRNSYVVKYRSRFP-AKKWW-QSVFERL
Pgbd3-Hs_NP_736  308 YSQ---KLKKKIQVQQPNMIKVYNQFMGGV-DRADENIDKYRASIR-GKKWY-SSPLLFC
Pgbd1-Hs_NP_001  315 CDA---DNEEIPQISQPSIVKVYDECKEGV-AKMDQIISKYRVRIR-SKKWY-SILVSYM
Pgbd2-Hs_NP_733  310 HSG---AAKTRTQVHQPSLVKLYQEKVGGV-GRMDQNIAKYKVKIR-GMKWY-SSFIGYV
Bmo_BAD11135     316 ------IDESTGEKQKPEMITFYNSTKAGV-DVVDELCANYNVSRN-SKRWP-MTLFYGV
Ago_ADU04477     308 ------DSKIDVETRKPEIIIDYNCTKGGV-DTVDKMCAAYSVSRI-TKRWP-LVIFYSL
Hvi_ABD76335     308 ------NNIVESEKKKPEIILYYNSTKGGV-DTNDQMCANYNVGRR-TKRWP-MVIFYHL
Pgo_ADB45159     306 ------QPDINQETGKPEMIHFYNSTKGAV-DTVDQMCSSISTNRK-TQRWP-LCVFYNM
Tni_AAA87375     304 ------DASINESTGKPQMVMYYNQTKGGV-DTLDQMCSVMTCSRK-TNRWP-MALLYGM
Cag_ADV17598     305 ------DAVVNQSNGKPDMILFYNQTKGGV-DSFDQMCSSMSTNRK-TNRWP-MAVFYGM
Aip_ADV17599     306 ------NAVINESNGKPDMILFYNQTKGGV-DSFDQMCKSMSANRK-TNRWP-MAVFYGM
Har_ABS18391     306 ------NAVINESNGKPDMILFYNQTKGGV-DSFDQMCKSMSANRK-TNRWP-MAVFYGM
Hma_XP_00421184  300 ------NDIVMDHNEKPCIILDYKKYKGGI-DTLDRVVRCYSSRRK--------------
Tru_Pigibaku1_X  299 ------GRMCDQEHHKPEIIMDYNATKGGV-DNMDKLVTAYSCKWR-TLRWP-LVIFFDM
Oni_XP_00545891  297 ------PDVSTEGKRKPVIIQDYNKCKGGV-DKLDQVVGTYSCRRR-TNYWP-LALFHNL
Pny_XP_00575514  297 ------PEVSDGEKRKPVIIDDYNQCKGGV-DNLDKVVGTYSCRRR-TNRWP-MAVFHNM
Mlu_Mitra        302 ------DRTSKKKIVKPNALIDYNKHMKGV-DRADQYLSYYSILRR-TVKWT-KRLAMYM
PLE-wu_ref       310 RKG---RDGSKTDVRCPAVVKEYTKRMGGV-DHFDHIKGTYSVGRR-SKRWW-LRIFYFI
Aca_XP_00509140  311 ------TKEGVVPQTIPTPVHDYNQNMSGV-DLADQYRSYYNIGRP-GKKWW-RYGVWFL
Ami_ACT79641     314 RWK---KNGDVVLVEKPACVDLYNTSMGGV-DRTDQLRSYYSACRP-SKKWY-KYLFWFI
Nap_EQB62075_PG  326 ------FVTGTNGKVKPDAVFDYNIAKKGV-DLSDQLSGYYSCLRK-TIKWYRKVVIQLI
Goc_XP_00374069  312 V-----AKKDGVQKVKPLAVIEYNKAKQGV-DISDQLASFYSPLRK-TIRWYHKVVFELL
Mro_XP_00370823  304 ------VKTKRGFCCKPKIIVEYNKAKTSI-DLSDQMSAYSSPLRR-TLKWYKKLAFELC
Xbo_BAF82021_Ur  335 ------HRRGRPAKSKPLCCKEYSKHMGGV-DKTDQIQTYYDATRK-TRAWY-KKAAIYM
Mfa_EHH62949_PG  318 ------NNRNGKKTKKPRVIVDYNENMGAV-DSADQMLTSYPSERKRHKVWY-KKFFHHL
Ggo_XP_00405596  318 ------NNRNGKKTKRPRVIVDYNENMGAV-DSADQMLTSYPSERKRHKVWY-KKFFHHL
Pgbd4-Hs_NP_689  318 ------NNRNGKKTKRPRVIVDYNENMGAV-DSADQMLTSYPSERKRHKVWY-KKFFHHL
Sbo_XP_00393580  318 ------NNRNGKKTKKPRVIVDYNENMGAV-DSADQMLTSYPSERKRHKVWY-KKFFHHL
consensus        541                kp ivv yn  k av D  dql   y   h    hw    if  m
```

```
PgmL2b-Psex_PSE   328 KKLQFGTVSSLLK-------KYLKGQFSNQLFRIQTYSSQII------------------
PgmL2a-Psex_PSE   346 QEIVIWNSFIAFKSI-----QKVNSQINFLEFRLT-LAKQLL------------------
PgmL2-Ptet_PTET   346 QEIVIWNSFIAFKSI-----QKVNSQINFLEFRLT-LAKQLL------------------
PgmL2-Pbi_PBIGN   346 QEIVIWNSFIAFKSI-----QKVNSQINFLEFRLT-LAKQLL------------------
PgmL1-Pcau_PCAU   302 QEIIWNLWVLTGQ-----------KQQSYEQFRLT-FSQELL------------------
PgmL1-Psex_PSEX   305 QEITIWNSYQIQAKI-----KGSSNQQTYEQFRLG-LAQELL------------------
PgmL1-Ptet_PTET   305 QEITIWNSYQIVMKL-----KASQHQQTYEQFRLG-LAQELL------------------
PgmL1-Pbi_PBIGN   305 QEMTIWNSYQIFIKI-----KSLQHQQTYEQFRLG-LAQELL------------------
Lia5_TTHERM_006   327 SEIAIQNSYIIY--------SQAKEKMDYRLFRFK-LAQDLL------------------
PgmL5a-Ptet_PTE   400 IESILHNIRILKKQ-------------EIKTFRTE-LALQLI------------------
PgmL5a-Pbi_PBIG   398 IESILHNIRILKKQ-------------EIKTFKTE-LALQLI------------------
PgmL5b-Ptet_PTE   399 IESILHNIRILKKQ-------------EIKSFRTE-LALQLI------------------
PgmL5b-Pbi_PBIG   398 IESILHNIRILKKQ-------------EIKTFKTE-LALQLI------------------
PgmL5b-Psex_PSE   398 IESILHNIRILKKQ-------------EIKSFRTE-LALQLI------------------
PgmL5a-Psex_PSE   398 IESILHNIRILKKQ-------------EIKTFRTE-LALQLI------------------
PgmL3c-Psex_PSE   322 FEIMIYNTSLMFDD------------FMQSKFRLS-LAKIFT------------------
PgmL3c-Ptet_PTE   320 FEVMIYNTSLLIED------------MLQSNFRLS-LAKIFT------------------
PgmL3c-Pbi_PBIG   322 FEVILYNSTLLIEDMLQ------------SNFRLN-IAKILS------------------
PgmL3-Pcau_PCAU   320 FEVMIHNTFLLFQY------------PSYKSYRHE-LAQVLL------------------
PgmL3a-Pbi_PBIG   318 FEIMIHNTFLLIPQ------------TSQSQFRHD-LAKVLL------------------
PgmL3a-Ptet_PTE   318 FEIMIHNTFLLIQQ------------TSQSQFRHD-LAKILL------------------
PgmL3b-Ptet_PTE   319 FEIMIHNTFLLIQQ------------TSPSQYRHD-LAKVLL------------------
PgmL3b-Pbi_PBIG   319 FEIMIHNTFLLIQQ------------TSQSQFRHD-LAQVLL------------------
PgmL3a-Psex_PSE   321 FEIMIHNTFLLIQQ------------TSPSQFRHD-LAKVLL------------------
PgmL4b-Psex_PSE   477 VYSSVYNANILNKIKNQ---SGPLAPDRSKQMYLE-FVKQLL------------------
PgmL4a-Psex_PSE   479 VYSSIYNANILNKIKNQ---SSSLAPDKAKQMYLE-FVKQLL------------------
PgmL4b-Ptet_PTE   477 VYSSIYNANILNKTKNQ---QTTLAPEKAKQMYLE-FVRQLL------------------
PgmL4b-Pbi_PBIG   478 VYSSIYNANILNKIKNQ---QSSLAPDKAKQMYLE-FVKQLL------------------
PgmL4a-Ptet_PTE   477 VYSSIYNANILNKIKNQ---SASLTPEKAKQMYLE-FVKQLL------------------
PgmL4a-Pbi_PBIG   477 VYSSIYNANILNKIKNQ---SSSLTPEKAKQMYLE-FVKQLL------------------
Tpb7_TTHERM_006   344 LDVIVKNTHSLYQY------INNDSKITKEQIIKD-LKKQFLQSYQAHRELQHKICIEKI
Pgbd5-Rty_XP_02   369 LSIAVNNSYILYKMSE----AYLVRRYTRVQFGER-LVKELLGIEEHLPLE---------
Pgbd5-Dre_NP_00   370 ISIAINNAYILYKMSD----AYHVKRYSRAQFGER-LVKELLDMDDCSPTQ---------
Pgbd5-Hs_NP_001   369 ISIAINNAYILYKMSD----AYHVKRYSRAQFGER-LVRELLGLEDASPTH---------
Pgbd5-Gga_XP_01   369 ISIAINNAYILYKMSE----AYHVTRYSRAQFGER-LVKELLGLEDTSPSH---------
Tpb1_TTHERM_000   375 LDTTLCNAYIIYKILN----EGKKSLLTHKDFRIK-IVEELI------------------
Tpb6_DAA80465.1   386 LETSITNSYILYKLKFENTQNMQFSILTHKEFRLR-LIQEIS------------------
Tpb2_TTHERM_011   383 LDNAIVNAFILYNFN-----LSPKYKISQKEFRIQ-LFKELA------------------
Pgm-Pcau_PCAUDP   428 FETAILNAYLIFRSYNPE--SSYRNKGQMRDFRII-QCINLQ------------------
Pgm-Pbi_PBIGNP2   428 FETAILNAYLIFRSYNPE--SSYRNKGQMRDFRIN-LMYQFA------------------
Pgm-Psex_PSEXPN   428 FETAILNAYLIFRSYNPE--SSYRNKGQMRDFRIN-LMYQFA------------------
Pgm-Ptet_PTET.5   428 FETAILNAYLIFRSYNPE--SSYRNKGQMRDFRIN-LMYQFA------------------
Pgbd3-Hs_NP_736   362 FELVLQNAWQLHK-------TYDEKPVDFLEFRRR-VVCHYL------------------
Pgbd1-Hs_NP_001   369 IDVAMNNAWQLHRA------CNPGASLDPLDFRRF-VAHFYLEHNAHLSD----------
Pgbd2-Hs_NP_733   364 IDAALNNAWQLHRI------CCQDAQVDLLAFRRY-IACVYL------------------
Bmo_BAD11135      367 LNMAAINACIIYR-------TNKNVTIKRTEFIRS-LGLSMI------------------
Ago_ADU04477      359 MNIAGINAQVLFSYS-----KHNNAPKIRRLFLKT-LAFDLM------------------
Hvi_ABD76335      359 LNVAGINAYVIFK-------NKIDHGISRREFLKH-LAVDLV------------------
Pgo_ADB45159      357 LNLSIINAYVVYVYNNV---RNNKKPMSRRDFVIK-LGDQLM------------------
Tni_AAA87375      355 INIACINSFIIYSHNVS---SKGEKVQSRKKFMRN-LYMSLT------------------
Cag_ADV17598      356 LNMAFVNSYIIYCHNML---AKKEKPLSRKDFMKK-LSTDLT------------------
Aip_ADV17599      357 LNMAFVNSYIIYCHNKI---NKQKKPINRKEFMKN-LSTDLT------------------
Har_ABS18391      357 LNMAFVNSYIIYCHNKI---NKQEKPISRKEFMKK-LSIQLT------------------
Hma_XP_00421184   339 ------NNSLMYGI----------------GFR---------------------------
Tru_Pigibaku1_X   350 LDISAYNAFVIWMALNPE--WKGVKLQKRRLFLED-LGKELV------------------
Oni_XP_00545891   348 LDVSLYNAYVLWTSIEPS--WQKQKGYKRRLFIEE-VGEMLV------------------
Pny_XP_00575514   348 IDVSLYNAFVLWTSVDRS--WLQQTPHRRWLYIEE-VGESMI------------------
Mlu_Mitra         353 INCALFNSYAVYK-------SVRQRKMGFKMFLKQ-TAIHWLT-----------------
PLE-wu_ref        364 FDACITNSFLLQGK------NANATKLSNLEYRVA-LARGLI------------------
Aca_XP_00509140   362 IQTAIINAFLLMRRANPNA-RRRSPGADHLHFRIA-LLQDLL------------------
Ami_ACT79641      368 FDVSLVNSFIIFKEN-----VDRRGRRTLVNFRLA-LATQLI------------------
Nap_EQB62075_PG   378 CGTSLVNAWYIHK-------RWGTKNMNLLQFREV-IIDRLL------------------
Goc_XP_00374069   365 LNTAVVNSRIIFN-------KLTGRAMSMKTFRAA-IVEDFL------------------
Mro_XP_00370823   356 LNTAVVNSLFVFQ-------EVTGQKISITEFRRQ-LVNELT------------------
Xbo_BAF82021_Ur   386 IQMALYNSYVVYKA------AVPGPKLSLYNYLLQ-LLPALL------------------
Mfa_EHH62949_PG   370 LHITVLNSYILFKK------DNPEHTMSHINFRLA-LIERML------------------
Ggo_XP_00405596   370 LHITVLNSYILFKK------DNPEHTMSHINFRLA-LIERML------------------
Pgbd4-Hs_NP_689   370 LHITVLNSYILFKK------DNPEHTMSHINFRLA-LIERML------------------
Sbo_XP_00393580   370 LHITVLNSYILFKK------DNPEYTMSHINFRLT-LIERLL------------------
consensus         601 iel i nsfilfk              s r fri  la  ll
```

```
PgmL2b-Psex_PSE      -
PgmL2a-Psex_PSE      -
PgmL2-Ptet_PTET      -
PgmL2-Pbi_PBIGN      -
PgmL1-Pcau_PCAU      -
PgmL1-Psex_PSEX      -
PgmL1-Ptet_PTET      -
PgmL1-Pbi_PBIGN      -
Lia5_TTHERM_006      -
PgmL5a-Ptet_PTE      -
PgmL5a-Pbi_PBIG      -
PgmL5b-Ptet_PTE      -
PgmL5b-Pbi_PBIG      -
PgmL5b-Psex_PSE      -
PgmL5a-Psex_PSE      -
PgmL3c-Psex_PSE      -
PgmL3c-Ptet_PTE      -
PgmL3c-Pbi_PBIG      -
PgmL3-Pcau_PCAU      -
PgmL3a-Pbi_PBIG      -
PgmL3a-Ptet_PTE      -
PgmL3b-Ptet_PTE      -
PgmL3b-Pbi_PBIG      -
PgmL3a-Psex_PSE      -
PgmL4b-Psex_PSE      -
PgmL4a-Psex_PSE      -
PgmL4b-Ptet_PTE      -
PgmL4b-Pbi_PBIG      -
PgmL4a-Ptet_PTE      -
PgmL4a-Pbi_PBIG      -
Tpb7_TTHERM_006  397 S
Pgbd5-Rty_XP_02      -
Pgbd5-Dre_NP_00      -
Pgbd5-Hs_NP_001      -
Pgbd5-Gga_XP_01      -
Tpb1_TTHERM_000      -
Tpb6_DAA80465.1      -
Tpb2_TTHERM_011      -
Pgm-Pcau_PCAUDP      -
Pgm-Pbi_PBIGNP2      -
Pgm-Psex_PSEXPN      -
Pgm-Ptet_PTET.5      -
Pgbd3-Hs_NP_736      -
Pgbd1-Hs_NP_001      -
Pgbd2-Hs_NP_733      -
Bmo_BAD11135         -
Ago_ADU04477         -
Hvi_ABD76335         -
Pgo_ADB45159         -
Tni_AAA87375         -
Cag_ADV17598         -
Aip_ADV17599         -
Har_ABS18391         -
Hma_XP_00421184      -
Tru_Pigibaku1_X      -
Oni_XP_00545891      -
Pny_XP_00575514      -
Mlu_Mitra            -
PLE-wu_ref           -
Aca_XP_00509140      -
Ami_ACT79641         -
Nap_EQB62075_PG      -
Goc_XP_00374069      -
Mro_XP_00370823      -
Xbo_BAF82021_Ur      -
Mfa_EHH62949_PG      -
Ggo_XP_00405596      -
Pgbd4-Hs_NP_689      -
Sbo_XP_00393580      -
consensus        661
```
