# Supplementary material for: Six domesticated PiggyBac transposases together carry out programmed DNA elimination in Paramecium
Source: eLife. 2018 Sep 18;7:e37927. doi: 10.7554/eLife.37927 (PMC6143343; doi:10.7554/eLife.37927)
Supplement: Supplementary file 3. [file elife-37927-supp3.rtf]

Supplementary file 3. Sequences of the cysteine-rich domains used for the alignment shown in Figure 1-figure supplement 1

>Pgbd3Hs_NP_736609.2
HVIVKQGKQTRCAECHKNTTFRCEKCDVALHVKCSVEYHTE

>Pgbd2Hs_NP_733843.1
DMIGHWIIHQDKRTRCALCHSQTNTRCEKCQKGVHAKCFREYHIR

>Mlu_Mitra PNAS 2013_PiggyBat
HTLQAIVGSGKKKNILRRCRVCSVHKLRSETRYMCKFCNIPLHKGACFEKYHTLKNYLE

>Pgo_ADB45159
PVPSVSNVRKIYYLCPSKARRMTKHRCIKCKQAICGPHNIDICSRCIE

>Tni_AAA87375
STEEPVMKKRTYCTYCPSKIRRKANASCKKCKKVICREHNIDMCQSCF

>Cag_ADV17598
DTSFDEPEPKKRRYCGFCSYKKKRMTKTQCFKCKKPVCGEHNIDVCQDCI

>Aip_ADV17599
NSEEPGPKKRSYCGFCSYKKRRMTKTQFYKCKKAICGEHNIDVCQDCV

>Har_ABS18391
ISNEPEPKKRRYCGVCSYKKRRMTKAQCCKCKKAICGEHNIDVCQDCI

>Tru_Pigibaku1_XP_011603527.1
AEPEVVNTSNKKKRCEVCGPKMDRKTQYTCIKCKKYICNTHTVKLCPSCVV

>Oni_XP_005458919_PGBD4 ?
PSPIIKCKGRRQCELCKEKRRRIVNTCCKCEKYTCKDHSVSICNNCSA

>Pny_XP_005755147_PGBD4 ?
AGPSGLTHPKGRKRCELCCDYMRRVGNSCSKCGRFTCRAHSKFICSHCST

>Bmo_BAD11135
PSPRHVNVPGRYVRCQDCPYKKDRKTKRSCNACAKPICMEHAKFLCENCAELDSSL

>Ago_ADU04477
SDVSTTPPSKRGTCFECGRKKNAATSMKCTKCMRFVCKLHSKKIIICEKCSNNDDNGNSE

>Hvi_ABD76335
NAEVQDPGSTSRGGPSTSYKRCHICPRSKDKKIRFMCAKCHHHICHDHSTMICDKCID

>PLEwu_ref ?
RFSNVGDHMPNDIPSYQRCRYCSTKAKDKRSKIKCSKCGVPLCITPCFSNFHKQV

>Aca_XP_005091401_PGBD4 ?
HKLVKIPGRKKTCYVCSKAKRKTNKGRGVETVYGCTYCGIHLCKGRCFTTYHRSLELDEPL

>Ami_ACT79641
PENAPGHFIIRREGNARKRHCVQCKKDGIKTPSNRAKETIYECAQCGIALCKDPCFLRFHSL

>Xbo_BAF82021_Uribo
HIPPTPNKRYAQRTCKVCRSRGVRKDVRYFCAKCPSKPALCFEPCFELYHTVVH

>Sbo_XP_003935808_PGBD4 ?
HFPKSIPPTSGKQNPTGRCKICCSQYDKDGKKIRKETRYFCAECDVPLCVVPCFEIYHTQKNY

>Mfa_EHH62949_PGBD4 ?
HFPKSIPPTSGKQNPTGRCKICCSQYDKDGKRIRKETRYFCAECDVPLCVVPCFEIYHTKKNY

>Ggo_XP_004055963_PGBD4 ?
HFPKSIPATSGKQNPTGRCKICCSQYDKDGKKIRKETRYFCAECDVPLCVVPCFEIYHTKKNY

>Pgbd4Hs_NP_689808.2
HFPKSIPATSGKQNPTGRCKICCSQYDKDGKKIRKETRYFCAECDVPLCVVPCFEIYHTKKNY

>Nap_EQB62075_PGBD4 ?
HVLQSYEGSARESRKRCKECYKKLSEKKGRDYATNKTTRVKTYCGQCKGQPALCLKCFNKLHKKQ

>Goc_XP_003740690_PGBD4 ?
HSLITSERKDMRGRKIRGKCQDCYSRLSTSRGRISAQNTVKKVNTRCQECQRWLCVPCFTKSHQERR

>Mro_XP_003708235_PGBD4 ?
KKEGDVHKVRKYCSGCYAENSRLFGPKIAKNLTKKVVTFCYMCKSQPYFCLECFNKAH

>Tpb2_TTHERM_01107220
KNCDYSAHILVRSRTKKKSCIECKQLTLFSCSTCSNMFKMRIPLCQSGFNQCYDFHASKTYEEVV

>PgmPcau_PCAUDP00182
HIPLQKVKKLLNVLNVCTILKAEIVGLCDEKDTIKCQRFHEFMDFELDK

>PgmPsex_PSEXPNG08735
KIQPHTFIEGEEIVKCSECGNETKVFCQECTILKAEVVGLCHEKDTIKCQRFHEFMDFELDK

>PgmPtet_PTET.51.1.P0490162
KIQPHTFIEGEEIVKCSECGNETKVFCQECTILKAEVVGLCHEKDTIKCQRFHEFMDFELDK

>PgmPbi_PBIGNP26884
KIQPHTFIEGEEIVKCSECGNETKVFCQECTILKAELVGLCHEKDTIKCQRFHEFMDFELDK

>Tpb7_TTHERM_00616500
HFWEENQGGKKQECIVCHTKTRNYCIQCSEKKKQIIGFCGNSNCLQKHNELPAKLLN

>PgmL4bPsex_PSEXPNG14728
NHTLESGDTGTFSCIECGESSQSICRDCSNHFQMIIPVCRNKNELCLKSHIEMLANQ

>PgmL4aPtet_PTET.51.1.P0340197
NHTLESGDTGTFSCIECGESSQTICRECSNHFQMLIPVCRSKNEQCLRSHIEMLASQ

>PgmL4aPbi_PBIGNP34177
NHTLESGDTGTFSCIECGESSQTICRECSNHFQMLIPVCRSKNEQCLKSHIEMLASQ

>PgmL4aPsex_PSEXPNG18382
NHTLESGDTGTFSCIECGESSQTICRECSNHFQMLIPVCRNKNEQCLKSHIEMLINQ

>PgmL4bPtet_PTET.51.1.P0480099
NHTLESGDTGTFSCIECGESSQTICRECSNHFQMLIPVCRSKNEQCLKSHIEMLVNQ

>PgmL4bPbi_PBIGNP06829
NHTLESGDTGTFSCIECGESSQTICRECSNHFQMIIPVCRSKNEQCLKSHIEMLINQ

>PgmL5bPsex_PSEXPNG34282
DQLYHCPIHNGNARCQVCLSKSILSKTTASCIGCNKVLGTNIFLCIYPCFRLFHLNPKLYLKE

>PgmL5aPsex_PSEXPNG18824
DQLYHCPIHNGNARCQVCLSKSILSKTTASCIGCNKVLGTNIFLCIYPCFRLFHLNPKLYLKE

>PgmL5aPtet_PTET.51.1.P0570051
DQLYHCPIHNGNARCQVCLSKSILSKTTASCLGCNKVLGTNIFLCIYPCFRLFHLNPKLYLKE

>PgmL5aPbi_PBIGNP21060
DQLYHCPIHNGNARCQVCLSKSILSKTTASCLGCNKVLGTNIFLCIYPCFRLFHLNPKLYLKE

>PgmL5bPtet_PTET.51.1.P0510172
DQLYHCPIHNGNARCQVCLSKSILSKTTASCLGCNKVLGTNIFLCIYPCFRLFHLNPKLYLKE

>PgmL5bPbi_PBIGNP17746
DQLYHCPIHNGNARCQVCLSKSILSKTTASCLGCNKVLGTNIFLCIYPCFRLFHLNPKIYLKE

>Lia5_TTHERM_00653910
NIRQGGTHVQKKDGKQGICLVCLQEKNIQNNTFITCQECSLQNKKPVYLCDKCFEVYHLEINVNRDN

>Tpb1_TTHERM_000309879
HFLERQNQVGLCSLCKQATFFTCESCNYGNKKIALCPVNCHKEHMKKVYNLID

>Tpb6_DAA80465.1_MICspecific
QELFNQSTKFQLNHFVIFSKYKQKCCICKKITKFACDTCIDPLLNQKLNLCPGFCQKTHMLSFFKNQK

>PgmL2aPsex_PSEXPNG15036
HIPRKCNPPAKLQNKLVCLVCKKVPTEMVECESCSEISGKLITLCATECFSLFHQEPKKFVQS

>PgmL2Ptet_PTET.51.1.P0380073
HVPRKCNPPAKLQNKLVCLVCKKVPTEMVECESCSEISGKLITLCATECFSLFHQEPKKYVQS

>PgmL2Pbi_PBIGNP02170
HVPRKCNPPAKLQNKLVCLVCKKVPTEMVECESCSEISGKLITLCASECFSLFHQEPKKYVQS

>PgmL1Pcau_PCAUDP11456
HIPTILATPKSYAGSMNCLVCKRNTQLITQCKSCSEISGKLVILCACDCFYLFHQNTTEYIIN

>PgmL1Psex_PSEXPNG13991
HIPTIMATPKTYAGSMNCLVCKRNTQLITLCKPCSEISGKLVILCACDCFYLFHQNTLEYIIN

>PgmL1Ptet_PTET.51.1.P0110267
HIPTIMATPKTYAGSMNCLVCKRNTQLITLCKPCSEISGKLVILCACDCFYLFHQNTLEYIIN

>PgmL1Pbi_PBIGNP25908
KSLFHIPTIMATPKTYAGSMNCLVCKRNTQLITLCKPCSEISGKLVILCACDCFYLFHQNTLEYIIN

>PgmL3cPsex_PSEXPNG04876
GELYHTPIPQKQEESFKYCVVCMKFNSLTKPFYRCRLCEKIFNENKIFLCAFPCFELFHRNPSDFIDC

>PgmL3cPtet_PTET.51.1.P0020217
SESYHTPIPQKQEESFKYCVVCMKFNQLTKPFYRCRLCEKLLNENKIFICAFPCFELFHRNPSQFIEC

>PgmL3cPbi_PBIGNP33931
SESYHTPIPQKQEESFKYCVVCMKFNQLTKPLYRCRLCEKLFNENKLFLCAFPCFELFHRNPCNFIEC

>PgmL3Pcau_PCAUDP05663
GEEFHTPFPQKQDDTFKYCLVCMKFEGLTKPYYRCNLCEKLLNQSKIFLCPFPCFELFHRNPSDFISC

>PgmL3bPtet_PTET.51.1.P0080308
ADQFHTPIPQKQEDTFKYCLVCMKFEGLTKPYYRCQLCEKILNVNKIFLCPFPCFELFHKNPSDFVDC

>PgmL3bPbi_PBIGNP32492
ADQFHTPIPQKQEDTFKYCLVCMKFDGLTKPYYRCQLCEKILNVSKIFLCPFPCFELFHRNPSDFVDC

>PgmL3aPsex_PSEXPNG03363
ADQYHTPIPQKQEDTFKYCLVCMKFDGLTKPYYRCQLCEKLLNVSKIFLCPFPCFELFHRNPSDFVDC

>PgmL3aPtet_PTET.51.1.P0010374
ADQFHTPIPQKQEDTFKYCLVCMKFEGLTKPYYRCQLCEKLLKVNKIFLCPFPCFELFHRNPSDFMVC

>PgmL3aPbi_PBIGNP31686
ADQFHTPIPQKQEDTFKYCLVCMKFDGLTKPYYRCQLCEKLLNISKIFLCPFPCFELFHRNPSDFLVC
