# Supplementary material for: Six domesticated PiggyBac transposases together carry out programmed DNA elimination in Paramecium
Source: eLife. 2018 Sep 18;7:e37927. doi: 10.7554/eLife.37927 (PMC6143343; doi:10.7554/eLife.37927)
Supplement: Supplementary file 4. [file elife-37927-supp4.rtf]

Supplementary file 4. Sequences of the transposase core domains used for the alignement shown in Supplementary file 1
>PgmL2b-Psex_PSEXPNG17601
QGERNAYNFWKSQVTNDAKDIYYSLFHRQIVQSILKMINDQMKDHFSEQHEKDVKKRLIKLDQIYEYFGVKILMGYNRMPDPEDYFNYKINKSRKIQIFRINTNLRDVVMIQAKYHNLKQEDVENKFKVEGEVYSYLTKKLNKKFRILYDAGQCLAIIKNNLFEAQDLQGRSIECILFMDVQTQYIIAIRFCYRENTTNTIFNMLDPYKHKNHKLYFQQELLSLEQVYSCGILAQSIQTQKIDFKDRLVTNNHVMSLKLLIPNQADFKVFASTADGFHRNTSQDKKSYAVVIYQEYIKLVEQFPALSDAYKCLFSSATIDGAIYTKCKKLQFGTVSSLLKKYLKGQFSNQLFRIQTYSSQII
>PgmL2a-Psex_PSEXPNG15036
QGERNAYNFWKSQVTNDAKDIYYSLFHRQIVQSILKMINDQMKDHFSEQHEKDVKKRLFKLDQIYEYFGVKILMGYNRMPDPEDYFNDKLPFRNKIGELIKVGRFKFLEQNTNLRDEVMIQAKYHNLKQEDVEKKFKVEGEVYSYLTKKLNKKFRISHDAGQCLAIIKNNLFEAQDLQGRSIECILLMDVQTQYIIAIRFCYRENISNTIFNMLDPYKHKNHKLYFQQELLSLEQVQILVEFFAIYSCGILAQSTQTQKIDFKDGLVTNNHVMLLKLLTPNQADFKVFASTADGFHRNTSQDKKSYAVVIYQEYIKLVEQFPALSDAYKCLFSSATVNGAIYTEMQEIVIWNSFIAFKSIQKVNSQINFLEFRLTLAKQLL
>PgmL2-Ptet_PTET.51.1.P0380073
QGDRNAYNFWKSQVTNDAKDIYYSLFHRQIVQSILKMINDQMKDHFSEQHEKDVKKRLFKLDQIYEYFGVKILMGYNRMPDPEDYFNDKLPFRNKIGELIKVGRFKFLEQNTNLRDEVMIQAKYHNLKQEDVEKKFKVEGEVYSYLTKKLNKKFRISHDAGQCLAVIKNNLFEAQDLQGRSIECILLMDVQTQYIIAIRFCFRENVSNTIFNMLDPYKHKNHKLYFQQELLTLEQVQILVEFFAIYSCGILASTTQTQKIDFKDGLVTNNHVMLLKLLTPNQADFKVFASTADGFHRNTSQDKKSYAVVIYQEYIKLVEQYPALSDAYKCLFSSATVNGAIYTEMQEIVIWNSFIAFKSIQKVNSQINFLEFRLTLAKQLL
>PgmL2-Pbi_PBIGNP02170
QGERNAYNFWKSQVTNDAKDIYYSLFHRQIVQSILKMINDQMKDHFSEQHEKDVKKRLFKLDQIYEYFGVKILMGYNRMPDPEDYFNDKLPFRNKIGELIKVGRFKFLEQNTNLRDEVMIQAKYHNLKQEDVEKKFKVEGEVYSYLTKKLNKKFRISHDAGQCLAVIKNNLFEAQDLQGRSIECILLMDVQTQYIIAIRFCFRENISNTIFNMLDPYKHKNHKLYFQQELLTLEQVQILVEFFAIYSCGILASTTQTQNIDFKDGLVTNNHVMLLKLLTPNQADFKVFASTADGFHRNTSQDKKSYAVVIYQEYIKLVEQYPALSDAYKCLFSSATVNGAIYTEMQEIVIWNSFIAFKSIQKVNSQINFLEFRLTLAKQLL
>PgmL1-Pcau_PCAUDP11456
EPWNLLQKESLVQIYKSLFAPKLVKYMMDQINQKMRESFQNQSEKQIKKKLIKIDEIYDFFGIKIIMGHIKMPTLEDYFDETQLFQCKIFDCFKEGRFKCLEQWSCFTDQELLRKENKQSLNEFSATRVCKKIGKIAISVKNPGRDLILQVNPGFIVQNGQQIILCDAQNKLILWSHFCETTENFTNLEIVQLLAHFKDKNHILYCYDNILTVEQIIFLKLEYKIQCVGSIKQVVNTPYHNSVISIINWQNETIAITAGDNLEEILVNQQNFRERMLYYSIHKNRPLFSYSSYSSGIMTELQEIIIWNLWVLTGQKQQSYEQFRLTFSQELL
>PgmL1-Psex_PSEXPNG13991
NETPIGIYNNIFPKKLIRYLIDQINVKMKTSFANQDEKQIRKKLVKIDQIFDFFGIKIIMGYIKMPHLEDYFNEKQLFQCKIFECFKEGRFKCLEQWSNFTDESLLKNINKVKPSNEFQTIRVCKKIGKTLKNFKAPGKDLILQVNTGFIIESQINAQQIILCDPKNKLIVWQYFCNNIQEEKVGLQIVYVLQQFKDNNHTLYVYDNILNMEQICFLKTKLKIQCVAMVDSLSASQLPQIPLYLDFMSIVKEQNEIYAVTSGEQIQQLICIYNQYKESMLLYCIHKFRPNFSYLHFSSAVMTELQEITIWNSYQIQAKIKGSSNQQTYEQFRLGLAQELL
>PgmL1-Ptet_PTET.51.1.P0110267
NETPMGIYNSIFPKKLIRYLIDQINVKMKTSFANQDEKQIRKKLVKIDQIFDFFGIKIIMGYIKMPHLEDYFNEKQLFQCKIFECFKEGRFKCLEQWSNFTDECLLKNINKAKPINEFQTIRVCKKIGKTLRNIKTPGKDLILQVNSGFIIEQSINSQQIMICDPKSKLIVWQYFCNNIQEEKVGLQIVHVLQQFKDNNHTLYIYDNILNLEQIAYLKIKLKIQCVQKLDSIQASQVPQIPLYLDYMSIIKEQNEVFAVTSGEQIQQLICTYNQYKETMLLYCIHKFRPNFSYIHYSSAIMTELQEITIWNSYQIVMKLKASQHQQTYEQFRLGLAQELL
>PgmL1-Pbi_PBIGNP25908
NETPMGIYNTIFPKKLIRYLIDQINVKMKTSFANQDEKQIRKKLVKIDQIFDFFGIKIIMGYIKMPHLEDYFNEKQLFQCKIFECFKEGRFKCLEQWSNFSDECLLKNINKVKPINEFQTIRVCKKIGKTLRNIKTSGKDLILQVNSGFMIEQSIHSQQIMICDPKSKLIVWQYFCNNIQDEKVGLQIVYLLQQFKDNNHTLYIYDNILNLEQIAFLKIKLKIQCIQKLDNMISSQLPQIPLYLDYMSIIKEQNEVFAVTSGDQIQQLICTYNQYKESMLLYCIHKFRPNFSYLHYSSAVMTELQEMTIWNSYQIFIKIKSLQHQQTYEQFRLGLAQELL
>Lia5_TTHERM_00653910
GKSLQLTNINDSSRLFMYFLGTKILRQLLNKANVKLREIYKKQNDVKEYTIEEIQVYQALKILMGLQQNSSNFDFFLLKDLQKIPPYHQLFTNERFQFLLDCERELNKDILNNEQLIQDFVQRAQNSQTSDQELVLISKKGKVGEEIIHNNSVYTQIFLCELSSAFVFGYFVVKDMSSFANQICINLESFTNQNHHVYFQNEEFFSNYEKIQELLNSKIHISSFLNNKFDQFPQSLQNEMLHNKPLKANNSETIFDRQTQTQLLIKSDENLKKEVFLTTSGTVKQDKMVEKHKQAIQKITQKLRMLLTEYRFVSINNDTTSIFEELSEIAIQNSYIIYSQAKEKMDYRLFRFKLAQDLL
>PgmL5a-Ptet_PTET.51.1.P0570051
GDNDNFQSQQVDKQQKITQLLQQPIIPAQVSNKPLNQQQRKHQALLEEIVQEDDGIVQDIENQDWTLFSQLNNMVPPKFSGQFRMLEYKDNPQDMIRLLFGETRFKDLMKTCQTSEPEFWLYLGTKLIMGYMRLPDINEYFYGEDWIAGGGIKSIITQQQFDDIDQKVEIIQPKIQTESQLAVKLEFNAQSFFRQEFLPQFTQELNDRFKKLILPGQELFLISNFYTIIYSTKIQWYQLIDKESGIILQQFFWATPINKALDLNSHRDLQKRLRMMFEPYHLGRHIVYSQGLLNAESVLQLYQQKIFVCTDLMQHSLLPHPPTGDQQVVYSSKNKEPIYLCYKNQQWITCVSLQSLQMRSQYLQELSQLQFTEEKIAIQPSEPENTSINSNSKIIFIFVIESILHNIRILKKQEIKTFRTELALQLI
>PgmL5a-Pbi_PBIGNP21060
GENDNFQSLQVDKQQKITQLLQQPIPPPQTKPLNQQQRKHQALLEEIVQEDDGIVQDIENQDWTLFTQLNSMIAPKFSGQFRMLEYKDNSQDMIRLLFGETRFKELMKTCQTSEPEFWLYLGTKLIMGYMRLPDINEYFYGEDWIAGGGIKSIITQQQFDDIDQRVEIIQPKLQTESQLAVKLEFNAQSFFRQEYLPQFTQELNDRFKKLILPGQELFLISNFYTLIYSTKIQWYQLIDKESGIILQQFFWATPINKALDLNSHRDLQKRLRMMFEPYHLGRHIVYSQGLLNAESVLQLYQQKIFVCTDLMQHSLLPHPPTGDQQVFYSSKNKEPIYLCYKNQQWITCVSLQSLQQRNQYLQELSQMQFTEEKIAIQPSEPENTSINSNSKIIFIFVIESILHNIRILKKQEIKTFKTELALQLI
>PgmL5b-Ptet_PTET.51.1.P0510172
GENDNFASHQIDKQQKITQLLQQPIPPPVQNKPLNQQQRKHQALLEEIVQEDDGIVQDIENQDWTLFTQLNNMITPKFSGQFRMLEYKDNTQDMIRLLFGETRFKDLMKTCQTSESEFWLYLGTKLIMGYMRLPDINEYFYGEDWIAGGGIKSIITQQQFDDIDQRVEIIQPKLQTESQLAVKLEFNAQTFFRQEYLPQFTQELNDRFKKLILPGQELFLISNFYTLIYSTKIQWYQLIDKESGIILQQFFWATPLSKALDLNNHRDLQKRLRMMFEPYHLGRHIVYSQGLLNAESILQLYQQKIFVCTDLVQHSLLPHPPTGDQQVLYSSKSKEPIYLCYKNQQWSTCVSLHSLQQRNQYLQELSQMQFAEEKIAIQPSEPENASINSTSKIIFIFVIESILHNIRILKKQEIKSFRTELALQLI
>PgmL5b-Pbi_PBIGNP17746
GENDDFSSNQIDKQQKITQILQQPIPPPQNKPLNQQQRKHQALLEEIVQEDDGVVQDIESQDWTLFTQLNTMITPKFSGQFRMLEYKDNTQDMIRLLFGETRFKDLMKTCQTSESEFWLYLGTKLIMGYMRLPDINEYFYGEDWIAGGGIKSIITQQQFEDIDQRLEIIQPKLQTESQLAVKLEFNAQAFFRQEYLPQFTQELNDRFKKLILPGQELFLISNFYTLIYSTKIQWYQLIDKESGIILQQFFWATPINKALDLNNHRDLQKRLRMMFEPYHLGRHIVYSQGLLNAESILQLYQQKIFVCTDLMQHSLLPHPPTSDQQVFYSSKSKEPIYLCYKNQQWSTCVSLPSLQQRFQYLQELSHMQFAEEKIAIQPSEPENASINSTSKIIFIFVIESILHNIRILKKQEIKTFKTELALQLI
>PgmL5b-Psex_PSEXPNG34282
GEIENLTLNQIDKQQKITQLLQQPIACPQNKPLNQQYKKHQALLEEIVQEDDGIVQDVDYQDWSLFNQLNNMTVPKFSGQFRMLEYKDNPQEMIRLLFGESRFKDLMKTCQTNESEFWLYLGTKLIMGYMRLPDINEYFYGEDWIAGGGIKSIITQQQFEDIDQKVEIIQPKLQTESQLAVKLEFNAQTFFRQEFLPQFTQELNDRFKKLILPGQELFLISNFYTLIYSTKIQWYQLIDKESGIILQQFFWATPINKTLDLNNHRDLQKRLRMMFEPYHLGRHIVYSQGLLNAESVLQLYQQKIFVCTDLNQHSLLPHPPNADQQIFYSSKSKEPIYLCYKNQQWNSCVSLQSLQSRNLYLQEISQQQFTEEKIAIQPSEPENTSINSTQKIIFIFVIESILHNIRILKKQEIKSFRTELALQLI
>PgmL5a-Psex_PSEXPNG18824
GENENFSSNQMDKQQKITQLLQQPTPIPQAKPLNQQQKKHQALLDDIVQEDDGIVQDIDYQDWSLFNQANNMTAPKFSGQFRMLEYKDNPQDMIRLLFGEIRFKDLMKTCQTSESEFWLYLGTKLIMGYMRLPDINEYFYGEDWIAGGGIKSIITQQQFDDIDQKVEIIQPKLQTESQLAVKLEFNAQTFFRQEYLPQFTQELNDRFKKLILPGQELFLISNFYTLIYSTKIQWYQLIDKESGIILQQFFWATPMNKTLDLNNHRDLQKRLRMMFEPYHLGRHIVYSQGLLNAESVLQLYQQKIFVCTDLVQHSLLPHPPTSDQQIFYSSKSKEPIYLCYKNQQWITCVSLQSLQQRNQYLQELSQTQFTEEKIAIQPSEPENTSINSTSKIIFIFVIESILHNIRILKKQEIKTFRTELALQLI
>PgmL3c-Psex_PSEXPNG04876
SQYSAKKLIQLQTPQNIYDHFIPNSLIGTLTELVNQYLLFILEQDLKSQPSNIQRQFIKKKYKETEIQLYFGLQILFGIYRFPQIDDYWNAEPWLRSGIEYMMPIGRFKFVDTHLFLYLNEKAKSKLQDEIQRLQKSVKCLCQPEQELILIEQIYKAYIAYYLFDFYKLFVIDLIIVSHRVKQEDRINRLMKMLFKYSNHNHVLYIMFELSLERIIQLTDQEIFPVISYKKTQHELFQNLQSGQYQIEQLYLIKNQYQVHLFPQKQLISNKHFLSVYQEIQQQYNDNQIMNLLQYRSNFYLCSQLNSSEGFNQLRIEFEEYFEIMIYNTSLMFDDFMQSKFRLSLAKIFT
>PgmL3c-Ptet_PTET.51.1.P0020217
YHAKKIVQLQTPQNVYDHFFPTSLIVTLTDLVNQYLFSILEEDLKQQPSKIASQYNKKKYKETEIQLYFGLQILFGIFRFPYIDDYWNAEPWLRCGIEYMMPIGRFKFLDTHLFMYLNQNAKSKLLNEVLQFQKQVKSICQPEQELILIEQIYKAYTAYYLFDFSRLIIIDLIVVSDRIQQEDRINRLMRMLFKYSNHNHILYVMFELSLERIIQLTDQDIYPVITYEKRLHQTIQNLLPGEYQIEQLYLIKNQYQVHLFPQKQSISNQSFLKVYKDVQQQYYEHSIMNLLQYRSNFYLCSQLNSVEGFNQLRIEFEEYFEVMIYNTSLLIEDMLQSNFRLSLAKIFT
>PgmL3c-Pbi_PBIGNP33931
SQYPAKRQVQPQTPQNTYDHFFPNSMIVTLTDLVNQYLLAILEEDLKQQPSKIPSQYHKKKYKETEIQLYFGLQILFGIFRFPYIDDYWNQEPWLRCGIEHMMPIGRFKFLDTHLFMYLNEKSKSKLQNELQQLQKSVKSICQPEQELILIEQIYKAYTVYYLFEFQRLFIIDLIVVSHRIHFEDRINRLMRMLFRYSNRNHILYVMFELNLERIIQLTDQNIYPVISYQKIQHLTIQNLLPGEYQIEQLYLIKNQHQAHLFPKKQLISNKSFLKVYKDVQQQYNENPIMNLLQYRSNFYLCSQLNNTEGFNQFRIEFEEYFEVILYNSTLLIEDMLQSNFRLNIAKILS
>PgmL3-Pcau_PCAUDP05663
LDAPQLKMSTLQSPKNVIHYFIPNDFISSLTEMINKYLMAALLQDQKQQTQQTQQLYNKKKYKDTEIQFYLGLQILFGMYRFPQLDEYWNAEPWLRGGIEISMPIGRLKFVDMYLCSHDEETQRATISSQLKKFKQKIKALYNPDQELVVTQQIYKAYTTYYLFDCESSQILDLLVVVHGNEEEKLNKIIRMLLKYANQNHVMYFMFEIKLEKIIQLVDQGIYPVIYYKNLQHSLIQTLNDGEYSLDQLYLIKQGQEIYIFPQKRLISHKHYVTNHRSIQEEVSEHLLNIQQFRSQFYFSSQLNSPEGFQQLRIEFEEYFEVMIHNTFLLFQYPSYKSYRHELAQVLL
>PgmL3a-Pbi_PBIGNP31686
TPQLKMTSFQSPQNVLNYFLPSSFVSTITEMINHYLLMVLQEDLKQQHQTQQIYHKKKYKESEIQFYLGLQILFGVYRFPSLEEYWNAESWLKGGVEVTMPIGRFKFVDLHIFSFFDELQRAQLQLEVSKFSKKLKALYNPDQELIVVEQNQKAYKTYYIFDYDSSQIIDLLVVCNNVKNEDRINRVMRMLYKYSNQNHVIYIMFDLSLEKIIQLVDQSIYPVIRYQKINHNLIQMLQDGEYELDQLFLIRQGIQVDVFPQRRLISNRHYMTSYEKIKNQLQEHIINLQQFRSNFYQSSQLNNTEGFHQLRVEFEEYFEIMIHNTFLLIPQTSQSQFRHDLAKVLL
>PgmL3a-Ptet_PTET.51.1.P0010374
TPQLKMTSLQSPQNVLNYFLPNSFVSTLTEMINHYLLMVLQEDVKQQQQTQQIYHKKKYKEAEIQFYLGLQILFGVYRFPSLDDYWNAESWLKGGVEVTMPIGRFKFVDLHIFSYFDEQQRAKLQLEVSKFSKKLKSLYNPDQELIIVEQNQKAYKAYYIFDYDSSQIIDLLVVCNNVKNEDRINRVMRMLYKYSNQNHVTYILFDLSLDKIIQLVDQSIYPVIRYQNISHNLIQMLQDGEYELDQLFLVKQGTQVDVFPQRRLISNRPYMTSYEKIKEQLQEHITNLQQFRSNFYQSSQLNNTEGFHQLRVEFEEYFEIMIHNTFLLIQQTSQSQFRHDLAKILL
>PgmL3b-Ptet_PTET.51.1.P0080308
TPQLKMTSLQSPQNVLNYFLPSSFVSTLTEMINHYLLTVLQEDLKQQQQQTQQIYHKKKYKEAEIQFYLGLQILFGVYRFPQLDDYWNAESWLRGGVEVTMPIGRFKFVDLHIFSYFDEVQRAKLQLEVRKFSKKLKALYNPDQELIVVEQNQKAYNAYYIFDYDSSQIIDLLVVCNNVKNEDRINRVMRMLYKYSNQNHVTYIMFYLSLERIIQLVDQSIFPVIRYKNITHNLIQMLQDGDYQLDQLFLVKQGTQVDVFPQRRLISSRHYMTSYEKIQEQLQEHIINLQQFRSNFYQSSQLNNTESFHQLRIEFEEYFEIMIHNTFLLIQQTSPSQYRHDLAKVLL
>PgmL3b-Pbi_PBIGNP32492
TPQLKMTSLQSPLNVLNYFLPCSFISTLTEMINHYLLMVLQEDLKQQQQQTQQIYHKKKYKEAEIQFYLGLQILFGVYRFPSLDDYWNAESWLKGGVEVTMPIGRFKFVDLHIFSYFDELQRAKLQLEVRKFSRKLKALYNPDQELIVVEQNQKVYKAYYIFDYDSSQIIDLLVVCNNVKNEDRINRVMRMLYKYSNQNHVTYIMFDLSLERIIQLVDQSIYPVIRYQNINHNLIQMLQDGDYELDQLFLIKQGTQVDVFPQRRLISSRHYMTSYEKIKEQLQEHIINLQQFRSNFYQSSQLNNTEQFHQLRVEFEEYFEIMIHNTFLLIQQTSQSQFRHDLAQVLL
>PgmL3a-Psex_PSEXPNG03363
LDTPQLKMTSLQSAQNVLNYFLPSSFVSTLTEMINHYLLMVLQEDLKQQQQQTQQMYHKKKYKEAEIQFYLGLQILFGVYRFPSLDDYWNVDSWLKGGVEVTMPIGRFKFVDLHIFSYFDELQRAKLQLEVRKFSKKLKALYNPDQELIVVEQNQKAYKAYYIFDYDSSQIIDLLVVCNNVKTEDRINRVMRMLYKYSNQNHVTYIMFDMSLERIIQLVDQSIYPVIRYQNVNHYLIQMLQDGDYELDQLFLIKQGTQVDVFPQRRLISNRHYMTSYEKIKEQLQEHITNLQQYRSNFYQSSQLNNTEGFHQLRVEFEEYFEIMIHNTFLLIQQTSPSQFRHDLAKVLL
>PgmL4b-Psex_PSEXPNG14728
MNHKFLGMNQNQSRFSNSPQRVRDMITQQYTSSYQVDRPPIMPSRQQPVFQQQTLEKNINYELNQVKKNKEQLSSHQQSQLRESQIKLQAKLQESVKRQSVAQPIIEDNDSDFMDDVMESQAFVDFKRAGNLINGSQQSNQQQYWQEINFNTDIPKEYYMNEKFRVEISQDIEISDPYELFKLYFDQSIFKYICQISNKRQCIRIDEDILESFVSALIYIFYIQLLGMREIKRVRFDHILDYVTFGHICKEIRIDELEDYSFIFEKISKNFKNYYQPEEFLTLDSPIFYQNHSVEGLISLSDGMKGYILDFIYGIKDQKIIQSLQKYQGKHHKLYLGPEVSSLNLINQLKKKQFGSLAKVVDKQTQLTYDQIQEVKQNAQKGKSTQLLSSDNQAIMLIYAEKQQHLQQVEGFVSSFADFTRLKPQDTKIKSDINKPIILYLYDKIKTQYDKRGKTYQYSQLPHQDANQHLEILVQLVYSSVYNANILNKIKNQSGPLAPDRSKQMYLEFVKQLL
>PgmL4a-Psex_PSEXPNG18382
LNHKFLGMNQNQSRISNSPQRVRDMITQQYTSSYQVDRPPVMPSRQQPVFQQQILEKNINYEINQVKKNKEQLSSHQQSQLRESQIKLQAKLQESVKRQSVAQPTIEENDSDFMDDIMDSQAFIDFKRAGNLMCGSQQSLQQQQQYWQEINFNTDIPKDYYMNEKFRVDVVEDIDINDPYELFKMYFDQSIFKYICQISNKRQCIRIDEDILESFISALIYIFYIQLLGMREIKRVRFDHIIDYVTFGHICKEIRIDELEDYSFIFEKISKNFKNLYQPDEFLTLDSPIFYQNHCVEGLISLSDGMKGYVLDFIYGIKDQKIIQSLQKYQGKHHKLYLGPEVSSLNLISQLKKKQFGSLAKVVDKQTQLTQDQMQEVKQNAQKGQSTQFLSSDNQTIMLIYAERQQHLQQVEGFVSSFADFTRLKPQDTKIKSDVNKPIILYLYDKIKTQYDKRGKTYQYSQLPHQDANQHLEILVQLVYSSIYNANILNKIKNQSSSLAPDKAKQMYLEFVKQLL
>PgmL4b-Ptet_PTET.51.1.P0480099
INHKFLGMNQNQSRFSNSPPRVRDMITQQYTSSYQVDRPPIMPSRQQPIFQQQILEKNITYELNQVKKNKEQLSSHQQSQLRESQIKLQAKLQESVKRQSVAQPTIEENDSDFLDEVMDSQAFIDFKKAGNLICGTQQSIQQQHWQEINFNTDIPKDYYMNEKFRVDVGEDIEICDPYELFKLYFDQSIFKYICQISNKRQCIRIDEDILESFITALIYIFYIQLLGMREIKRVRFDHIIDYVTFGHICKEIRIDELEDYSFIFEKISKNFKNNYQPEEFLTLDSPIFYQNHSVEGLISLSDGMKGYVLDFIYGIKDQKILQSLQKYQGKHHKLYLGPDVSSLNLISQLKKKQFGSLAKLVDKQTQLTQEQIQEVKQNAQKGKSTQFLSSDNQTIMLIYAERQQHIQQVEGFVSSFADFTRLKPQDTKIKSDVNKPIILYLYDKIKTQYDKRGKTYQYSQIPHQDANQHLEILVQLVYSSIYNANILNKTKNQQTTLAPEKAKQMYLEFVRQLL
>PgmL4b-Pbi_PBIGNP06829
LNHKFLGMNQNQSRISNSPSRVRDMITQQYTSSYQVDRPPNMPSRQQPIFQQQILEKNINYELNQVKKNKEQLSSHQQSQLRESQIKLQAKLQESVKRQSVAQPTIEENDSDFMDDVMDSQAFIDFKRAGNLICGSQQQQIQQYYWQEINFNTDIPKDYYMNEKFRVDVGQDIEIFDPYELFKLYFDQSIFKYICQISNKRQCIRIDEDILESFISALIYIFYIQLLGMREIKRVRFDHILDYVTFGHICKEIRIDELEDYSFIFEKISKNFKNNYQPEEFLTLDSPIFYQNHSVEGLISLSDGMKGYVLDFIYGIKDQKILQSLQKYQGKHHKLYLGPDVSSLNLISQLKKKQFGSLAKLVDKQTQLSQEQIQEVKQNAQKGKSTQFLSSDNQTIMLIYAERQQHLQQVEGFVSSFADFTRLKPQDTKIKSDVNKPIILYLYDKIKTQYDKRGKTYQYSQIPHQDTNQHLEILVQLVYSSIYNANILNKIKNQQSSLAPDKAKQMYLEFVKQLL
>PgmL4a-Ptet_PTET.51.1.P0340197
LNHKFLEMNQNQSRISNSPSRIRDMITLQYTSSYQVDRPPIMPSRQQPVFQQQTLERNINYELNQAKKNKEQLSGHQQSQLRESQIKLQAKIQESVKRQSVVQPTIEENDSDFMDDGMDSQPFIDFKRAGNIPCASQQQTYQQYWQEINFNTDIPKDYYMNEKFRVDLEQEIETSDPYELFKLYFDQSIFKYICLISNKRQCIRIDEDILESFISALIYIFYIQLLGMREIKRVRFDHILDYVTFGHICKEIRIDELEDYSFIFEKISKNFKSHYQPEEFLTLDSPIFYQNHSVDGLISLSDGMKGYVLDFIYGIKEQKILQSLQIYQGKHHKLYLGPDVSSLNLIIQLKKKQFGALAKVVDKQTQLTQDQIQEVKQNAQKGQSTQFLSSDNQTIMLIFAERQQHLQQVEGFVSSFADFTRLKPQDTKIKSDVNKPIILYLYDKIKTQYDKRGKTYQYSQIPHQNGNQHLEILVQLVYSSIYNANILNKIKNQSASLTPEKAKQMYLEFVKQLL
>PgmL4a-Pbi_PBIGNP34177
LNHKFLGMNQNQSRLSNSPSRVRDMITQQYTSSYQVDRPPNMPSRQQPVFQQQTLEKNINYELNQAKKNKEQLSSHQQSQLRESQIKLQAKLQESVKRQSNVQPTIEENDSDFMDDVMDSQAFIDFKRAGNLMCGSQQQMQQQYWQEINFNTDIPKDYYMNEKFRVDLEQEIETSDPYELFKLYFDQSISKYICLISNKRQCIKIDEDILESFISALIYIFYIQLLGMREIKRVRFDHILDYVTFGHICKEIRIDELEDYSFIFEKISKNFKNHYQPEEFLTLDSPIFYQNHSVEGLISLSDGMKGYVLDFIYGIKDQKIIHSLQNYQGKHHKLYLGPDVSSLNLITQLKKKQFGSLAKVVDKQTQLTQDQIQEVKQNAQKGQSTQFLSSDNQNIMLIYAERQQHLQQIEGFVSSFADFTRLKPQDTKIKSDINKPIILYLYDKIKTQYDKRGKTYQYSQLPHQSANQHLEILVQLVYSSIYNANILNKIKNQSSSLTPEKAKQMYLEFVKQLL
>Tpb7_TTHERM_00616500
WKFKGQDKVNLEYGKNYDASTIFRMLFTQPLYTHIIQNIYKKFNKRITKRNIFKDPIEINSYDIDAYISLLLHSQCLQHPNLINTINKINQDMDHHILEFIQNNFSVGAKVQDENGCFNEIAKIEQFISMVNQRLKQLVQPKEYLQLLHKTIEGNYFKSNSQYFLDEFRLYDYSNSFIVDVQFDKSTPELKKSQDYVQKLMRVQANVLDNYKGKGHKVVIQNQLFDADILRSLRHQNIGVVTRSHIENIQFTPKQLKIISENKLFKNFFFIYEREVQAVITPYSFSHKYAFISNFLGLEDTKDLANPKMQQIYSEKGVVLDYKDINPRDVEGVKWDQLVFLRVLDVIVKNTHSLYQYINNDSKITKEQIIKDLKKQFLQSYQAHRELQHKICIEKIS
>Pgbd5-Rty_XP_020370470.1
GPVHQMPLNASAIDFFQLFVPDNVIRNMVVQTNMYAKKYQERFGCDEGWSDVDLPEMKTFLGYMISTSIHHCESVLSIWSGGFYSNKSIALMMTQMRFEKILKYFHIVAFRSSQTTHGLYKIQPFLDCLQMTFDTAFKPSQTQVLHEPLIDEDPLFIATCTDRELRKRKKRKFSLWVRQCAATGYICQVYVNLKEGTASDGLDTLKNRPQLHSLVAKSLCQNLAGKNYIIYTGPSITSLNLFEEFEKKGIYCCGLLSSRKSDCTGLPQSMLVNPEIPQARGQYRVRMKGNMSLISWSNKGQFYFLTNAYSPFKEGVIIRRKSGEIRCPLAVEAFAAHLGYICKYDDKYSKYFISHKANKTWQQVFWFVLSIAVNNSYILYKMSEAYLVRRYTRVQFGERLVKELLGIEEHLPLE
>Pgbd5-Dre_NP_001139077.1
GPTQKMSATATAMDFFQLFVPDNVIQNMVTQTNMYAKKFQERFGSDEGWTNVTLAEMKAFLGYVTSTSVNRCESVLSIWSSGFFSNRSIALKMSQARFEKILKYFHVVAFRSSQGGSQGLYKIQPFLDSLQQSFSSSFRPSQTQVLHEPLIDEDPVFITTCTERELRKRKKRKFSLWVRQCSSTGFICQIYVHLKEGPGPDGLDTLKNKPQLHSLVAKQLCQNLAGRNAIIFTGPSITSLNLFQEFEKQGIYCCGLLSIRKSDCTGLPQSMLINTEAPQQRGQSHVKMRGNMSIINWYNKGNFRFLTNAYSPTKEGVIIKRKSGEIPCPLAVEAFAAHLSYICKYDDKYSKYFIFHKPNKTWQQVFWLTISIAINNAYILYKMSDAYHVKRYSRAQFGERLVKELLDMDDCSPTQ
>Pgbd5-Hs_NP_001245240.1
GPTRKMPPSASAVDFFQLFVPDNVLKNMVVQTNMYAKKFQERFGSDGAWVEVTLTEMKAFLGYMISTSISHCESVLSIWSGGFYSNRSLALVMSQARFEKILKYFHVVAFRSSQTTHGLYKVQPFLDSLQNSFDSAFRPSQTQVLHEPLIDEDPVFIATCTERELRKRKKRKFSLWVRQCSSTGFIIQIYVHLKEGGGPDGLDALKNKPQLHSMVARSLCRNAAGKNYIIFTGPSITSLTLFEEFEKQGIYCCGLLRARKSDCTGLPLSMLTNPATPPARGQYQIKMKGNMSLICWYNKGHFRFLTNAYSPVQQGVIIKRKSGEIPCPLAVEAFAAHLSYICRYDDKYSKYFISHKPNKTWQQVFWFAISIAINNAYILYKMSDAYHVKRYSRAQFGERLVRELLGLEDASPTH
>Pgbd5-Gga_XP_015139871.1
GPTRKMPLTASAMDFFQLFVPDNVLKNMVVQTNMYAKKYQERFGSDDTWIDVTLTEMKAFLGYMISTSIHHCESVLSIWSSGFYSNKSIALIMTQSRFEKILKYFHIVAFRSSQTTHGLYKIQPFLDSLQNGFDSAFRPSQAQVLHEPLIDEDPVFIATCTERELRKRKKRKFSLWVRQCSSTGFICQIYVHLKEGSGADGLDALKNKPQLHSMVAKSLCQNASGKNYIIFTGPSITSLNLFEEFEKQEIYCCGLLSARKSDCTGLPPSMLNNPDTPQSRGQYRIRMKGNMSLICWYNKGHFRFLTNAYSPVQQGVIIKRKSGEIPCPLAVEAFAAHLSYICKYDDKYSKYFISHKPNKTWQQVFWFAISIAINNAYILYKMSEAYHVTRYSRAQFGERLVKELLGLEDTSPSH
>Tpb1_TTHERM_000309879
KPQVKNIPLESTPVQIFQKLWSDEIWKLITDETNKYSKQSFDLNQYNLDSQSLKKQKICFFSQDQIKRFIICEILMGIQRLPSFSDYFSSDPLLSGGLNRILGRENYQLLTRYLHISDNQSRMVHVDDHTKFKQFQSILNRNYQQFYVPSNYLAIDEGIIPFKGKTKFKVYCPQKPVKFGIKEYLFCDYSGYTLNLIIHSPHEKQNIRDIYQPQTLQTQDIVNELIKDYQPLSGSILIMDNYYNSLNLIHDLNQQNIGVLGTVRPDRMNFTEEQKKMMKIQNFNKGETKSLTKDNIHIFLWRDKDKLVKMITNFLDNRKIVKSMKKTGQIKTIPLMVDIYNKYAHSVDKRNQICQNYRIHKRSQKWWKCVFYRLLDTTLCNAYIIYKILNEGKKSLLTHKDFRIKIVEELI
>Tpb6_DAA80465.1_MICspecific
PPQLIGLDECKTPKNFFDKIWDDRIWEMLTVYSNIYAEQYFQKKGIRMDQTFKDQIKQDKILYQLLKHFAPFTKNDIKRFIICEILQGIKRLPCFLDYWSTNPLISGGLNRFISKMKYNICVMFFHASDSSDISLKKDEIGKIKELQNLLNQNFSKYYKHSNFLAIDEGVIPFKGKSHLKVYCPGKPYKYGIKEFLLCDFTGYTILQEIATKKDKQNVYTKNSKDIKNYTHLLVKQLIHGQNNLQNCIIFMDNFYTSISLFEELSQNKIGAVGTVRLDRLKLNKEQKNEIKKDSLKQTGSISFMQKNMHLLFWYDNNRAVKILSNCIGMEQVKSQNIKNPIKDIPLMVKLYNKYSHSVDKRNQLLQYYRTIRRTRKWWKTIFYRLLETSITNSYILYKLKFENTQNMQFSILTHKEFRLRLIQEIS
>Tpb2_TTHERM_01107220
DLNRDFIPYNVPHSPYHLFRMFFDDRIYKLIIEETNRYKHQKYQEALLNLPPDKTLPKEAMDIDSIDLDAYLSILIFMGVQRMKSVKDYWKRKNYINSEIACIMKYSRFEQIDKHLHLADNEDPKIRSDPIGKVRQYMDYLNENFKKYYYPGEFLAIDEGMIPFNGKVAFKVYNPDKPDKFGIKEYVCCDSQNAYTLESQLYYGNHENEEFPEITLSKTNEVVMNLLKDYEGRFHKVVMDNYYNSPTLFYLMKQKSFGALGTMRIGRLKLPPYLLSQIMEVHQNKVLSYIQGDINLAIFFRGTNDKEICLMSNFIDQKKVKDDFWRVLAYVKEDRDIFKEQNLQSAYYNKYKGGVDRRNSYLAAYRNCRKNIKWYRPVFYRMLDNAIVNAFILYNFNLSPKYKISQKEFRIQLFKELA
>Pgm-Pcau_PCAUDP00182
GPRSIEKNKIKSEYDAFRLFFDNDIYATIIKHTRERYQQKVEEQIYSYIHGMVHMGIRAKKPSLMQWEFTEYELEAYFAVQIFFGIVRLSNQRDYWKSSARQRPIKKAETGRRKLRELAMEKMDRYAHWVTQRMSSIVSYERFKTIRNCLNISGAEALKLKGRDPIWKIRDFLNQMNTKFAKYYYPGEFITIDEGMIPFAGKVQFKVYNPDKPTKWGIKEYLLCDASNTYTFQLRLYHGQTMWNNDFKQTMFVNEEDTQHRTMELVLQMCKDYEHKAHKVVMDNYYSSWQLFRELRNRGIGAVGTIRHNRTGLTKKDLTSKHFQQIYNQYHYAYYMNQSNELMLMYFQGTSEKEIALISNFLDNSLNESHMWDISKQHYYVPHLKAPYMMYVYNKYKGGVDRRNSYVVKYRSRFPAKKWWQSVFERLFETAILNAYLIFRSYNPESSYRNKGQMRDFRIIQCINLQ
>Pgm-Pbi_PBIGNP26884
GPRSIDKNKIKSEYDAFRLFFDNDIYNTIIKHTRERYQQKVEEQIYSYIHGMVHMGIRAKKPSLMQWEFTEYELEAYFAVQIFFGIVRLSNQRDYWKSSARQKPIKKAETGRRKLRELAQEKMDRYAHWVTQRMSSIVSYEKFKTIRNCLNISGAEALKLKGRDPIWKIRDFLNQMNMRFAKYYYPGEFITIDEGMIPFAGKVQFKVYNPDKPTKWGIKEYLLCDASNTYTFQLRLYHGQTMWNNDFKQTMFVNEEDTQHRTMELVLQMCKDYEHKAHKVVMDNYYSSWMLFRELRNRGIGAVGTIRHNRTGLTKKDLTSKHFQQIYNQYHYAYYMNQSNELMLMYFQGTSEKEIALISNFLDNSLNEQHMWDISKQHYYVPHLKAPYMMYVYNKYKGGVDRRNSYVVKYRSRFPAKKWWQSVFERLFETAILNAYLIFRSYNPESSYRNKGQMRDFRINLMYQFA
>Pgm-Psex_PSEXPNG08735
GPRSIDKNKIKSEYDAFRLFFDNDIYNTIIKHTRERYQQKVEEQIYSYIHGMVHMGIRAKKPSLMQWEFTEYELEAYFAVQIFFGIVRLSNQRDYWKSSARQKPIKKAETGRRKLRELAQEKMDRYAHWVTQRMSSIVSYEKFKTIRNCLNISGAEALKLKGRDPIWKIRDFLNQMNMRFAKYYYPGEFITIDEGMIPFAGKVQFKVYNPDKPTKWGIKEYLLCDASNTYTFQLRLYHGQTMWNNDFKQTMFVNEEDTQHRTMELVLQMCKDYEHKAHKVVMDNYYSSWMLFRELRNRGIGAVGTIRHNRTGLTKKDLTSKHFQQIYNQYHYAYYMNQSNELMLMYFQGTSEKEIALISNFLDNSLNEQHMWDISKQHYYVPHLKAPYMMYVYNKYKGGVDRRNSYVVKYRSRFPAKKWWQSVFERLFETAILNAYLIFRSYNPESSYRNKGQMRDFRINLMYQFA
>Pgm-Ptet_PTET.51.1.P0490162
GPRSIDKSKIKSEYDAFRLFFDNDIYNTIIKHTRERYQQKVEEQIYSYIHGMVHMGIRAKKPTLMQWEFTEYELEAYFAVQIFFGIVRLSNQRDYWKSSARQKPIKKAETGRRKLRELAQEKMDRYAHWVTQRMSSIVSYEKFKTIRNCLNISGAEALKLKGRDPIWKIRDFLNQMNMRFAKYYYPGEFITIDEGMIPFAGKVQFKVYNPDKPTKWGIKEYLLCDASNTYTFQLRLYHGQTMWNNDFKQTMFVNEEDTQHRTMELVLQMCKDYEHKAHKVVMDNYYSSWMLFRELRNRGIGAVGTIRHNRTGLTKKDLTSKHFQQIYNQYHYAYYLNQSNELMLMYFQGTSEKEIALISNFLDNSLNEQHMWDISKQHYYVPHLKAPYMMYVYNKYKGGVDRRNSYVVKYRSRFPAKKWWQSVFERLFETAILNAYLIFRSYNPESSYRNKGQMRDFRINLMYQFA
>Pgbd3-Hs_NP_736609.2
APPNDFFTVMRTPTEILELFLDDEVIELIVKYSNLYACSKGVHLGLTSSEFKCFLGIIFLSGYVSVPRRRMFWEQRTDVHNVLVSAAMRRDRFETIFSNLHVADNANLDPVDKFSKLRPLISKLNERCMKFVPNETYFSFDEFMVPYFGRHGCKQFIRGKPIRFGYKFWCGATCLGYICWFQPYQGKNPNTKHEEYGVGASLVLQFSEALTEAHPGQYHFVFNNFFTSIALLDKLSSMGHQATGTVRKDHIDRVPLESDVALKKKERGTFDYRIDGKGNIVCRWNDNSVVTVASSGAGIHPLCLVSRYSQKLKKKIQVQQPNMIKVYNQFMGGVDRADENIDKYRASIRGKKWYSSPLLFCFELVLQNAWQLHKTYDEKPVDFLEFRRRVVCHYL
>Pgbd1-Hs_NP_001171672.1
FPSWSALDSGLLNLKSEKLNPVELFELFFDDETFNLIVNETNNYASQKNVSLEVTVQEMRCVFGVLLLSGFMRHPRREMYWEVSDTDQNLVRDAIRRDRFELIFSNLHFADNGHLDQKDKFTKLRPLIKQMNKNFLLYAPLEEYYCFDKSMCECFDSDQFLNGKPIRIGYKIWCGTTTQGYLVWFEPYQEESTMKVDEDPDLGLGGNLVMNFADVLLERGQYPYHLCFDSFFTSVKLLSALKKKGVRATGTIRENRTEKCPLMNVEHMKKMKRGYFDFRIEENNEIILCRWYGDGIISLCSNAVGIEPVNEVSCCDADNEEIPQISQPSIVKVYDECKEGVAKMDQIISKYRVRIRSKKWYSILVSYMIDVAMNNAWQLHRACNPGASLDPLDFRRFVAHFYLEHNAHLSD
>Pgbd2-Hs_NP_733843.1
DPHIEDLKSQELSPVGLFELFFDEGTINFIVNETNRYAWQKNVNLSLTAQELKCVLGILILSGYISYPRRRMFWETSPDSHHHLVADAIRRDRFELIFSYLHFADNNELDASDRFAKVRPLIIRMNCNFQKHAPLEEFYSFGESMCEYFGHRGSKQLHRGKPVRLGYKIWCGTTSRGYLVWFEPSQGTLFTKPDRSLDLGGSMVIKFVDALQERGFLPYHIFFDKVFTSVKLMSILRKKGVKATGTVREYRTERCPLKDPKELKKMKRGSFDYKVDESEEIIVCRWHDSSVVNICSNAVGIEPVRLTSRHSGAAKTRTQVHQPSLVKLYQEKVGGVGRMDQNIAKYKVKIRGMKWYSSFIGYVIDAALNNAWQLHRICCQDAQVDLLAFRRYIACVYL
>Bmo_BAD11135
QVKNIARDASTEYECWNIFVTSDMLQEILTHTNSSIRHRQTKTAAENSSAETSFYMQETTLCELKALIALLYLAGLIKSNRQSLKDLWRTDGTGVDIFRTTMSLQRFQFLQNNIRFDDKSTRDERKQTDNMAAFRSIFDQFVQCCQNAYSPSEFLTIDEMLLSFRGRCLFRVYIPNKPAKYGIKILALVDAKNFYVVNLEVYAGKQPSGPYAVSNRPFEVVERLIQPVARSHRNVTFDNWFTGYELMLHLLNEYRLTSVGTVRKNKRQIPESFIRTDRQPNSSVFGFQKDITLVSYAPKKNKVVVVMSTMHHDNSIDESTGEKQKPEMITFYNSTKAGVDVVDELCANYNVSRNSKRWPMTLFYGVLNMAAINACIIYRTNKNVTIKRTEFIRSLGLSMI
>Ago_ADU04477
GPKECARNIVSEIDAFLKIIDLDMIDEIVTCTNMYISNMRQRVQYSRPRDCLDTSRCEILAYFGLLFLIGIKKAHHANVKELWSADGSGTEITRATMSYKRFLFLCRCLRFDDRGTRAERRVIDKLSPIRTTFDLFLKNINKNYNLSEYTTIDEMLHPFRGRCQWIQYIPSKPAKYGIKMFALCDAKTFYTSKIEIYVGKQPPGPYEVSNSPIDIVKRLVTPIENSKRNLTTDNWYTSIPLADYLLQKKITLTGTLKKNKREIPTEFLPHKKKEVGSSVFGFQKNKTLVSYVPRKNKAVILLSTMHHDSKIDVETRKPEIIIDYNCTKGGVDTVDKMCAAYSVSRITKRWPLVIFYSLMNIAGINAQVLFSYSKHNNAPKIRRLFLKTLAFDLM
>Hvi_ABD76335
GPKGRAKEIQTISEAFFCMFSMDTVNLVLQQTNDYIKSIQEKFQRERDCKVLEYEELLAYLGLLYMSGVLRSSHLNFKDLWATDGTGIEFFQNTMSFNRFLFISRCVRFDDKNTKSERLKTDKLAAVREFTDLMNNNFINNYCASENVTLDEQLPAFRGRFSGVVYMPNKPTKYGIKHYALVDSATFYLLKFEIYAGVQPEGPYRMPNDTVSLVKRMTEPIWGTGRNVTMDNWFTSVPLANILLKDHQLTMVGTIRKNKPEIPTCFQPKRTRTEHSSLFGFQEDVTLCSYVPKKSKAVLLISSMHNDNNIVESEKKKPEIILYYNSTKGGVDTNDQMCANYNVGRRTKRWPMVIFYHLLNVAGINAYVIFKNKIDHGISRREFLKHLAVDLV
>Pgo_ADB45159
GPKERAREVSEPIDIFSLFISEDMLQQVVTFTNAEMLIRKNKYKTETFTVSPTNLEEIRALLGLLFNAAAMKSNHLPTRMLFNTHRSGTIFKACMSAERLNFLIKCLRFDDKLTRNVRQRDDRFAPIRDLWQALISNFQKWYTPGSYITVDEQLVGFRGRCSFRMYIPNKPNKYGIKLVMAADVNSKYIVNAIPYLGKGTDPQNQPLATFFIKEITSTLHGTNRNITMDNWFTSVPLANELLMAPYNLTLVGTLRSNKREIPEKLKNSKSRAIGTSMFCYDGDKTLVSYKAKSNKVVFILSTIHDQPDINQETGKPEMIHFYNSTKGAVDTVDQMCSSISTNRKTQRWPLCVFYNMLNLSIINAYVVYVYNNVRNNKKPMSRRDFVIKLGDQLM
>Tni_AAA87375
GPTRMCRNIYDPLLCFKLFFTDEIISEIVKWTNAEISLKRRESMTGATFRDTNEDEIYAFFGILVMTAVRKDNHMSTDDLFDRSLSMVYVSVMSRDRFDFLIRCLRMDDKSIRPTLRENDVFTPVRKIWDLFIHQCIQNYTPGAHLTIDEQLLGFRGRCPFRMYIPNKPSKYGIKILMMCDSGTKYMINGMPYLGRGTQTNGVPLGEYYVKELSKPVHGSCRNITCDNWFTSIPLAKNLLQEPYKLTIVGTVRSNKREIPEVLKNSRSRPVGTSMFCFDGPLTLVSYKPKPAKMVYLLSSCDEDASINESTGKPQMVMYYNQTKGGVDTLDQMCSVMTCSRKTNRWPMALLYGMINIACINSFIIYSHNVSSKGEKVQSRKKFMRNLYMSLT
>Cag_ADV17598
GPTRMCRNIVDPLLCFQLFIKEEIVEEIVKWTNVEMVQKRVNLKDISASYRDTNEMEIWAIISMLTLSAVMKDNHLSTDELFNVSYGTRYVSVMSRERFEFLLRLLRMGDKLLRPNLRQEDAFTPVRKIWEIFINQCRLNYVPGTNLTVDEQLLGFRGRCPFRMYIPNKPDKYGIKFPMVCDAATKYMVDAIPYLGKSTKTQGLPLGEFYVKELTQTVHGTNRNVTCDNWFTSVPLAKSLLNSPYNLTLVGTIRSNKREIPEEVKNSRSRQVGSSMFCFDGPLTLVSYKPKPSKMVFLLSSCNEDAVVNQSNGKPDMILFYNQTKGGVDSFDQMCSSMSTNRKTNRWPMAVFYGMLNMAFVNSYIIYCHNMLAKKEKPLSRKDFMKKLSTDLT
>Aip_ADV17599
GPTRMCRNIVDPLLCFQLFITDEIIHEIVKWTNVEMIVKRQNLIDISASYRDTNTMEMWALVGILTLTAVMKDNHLSTDELFDATFSGTRYVSVMSRERFEFLIRCMRMDDKTLRPTLRSDDAFIPVRKLWEIFINQCRLNYVPGGNLTVDEQLLGFRGRCPFRMYIPNKPDKYGIRFPMMCDAATKYMIDAIPYLGKSTKTNGLPLGEFYVKELTKTVHGTNRNVTCDNWFTSIPLAKNMLQAPYNLTIVGTIRSNKREIPEEIKNSRSRPVGSSMFCFDGPLTLVSYKPKPSRMVFLLSSCDENAVINESNGKPDMILFYNQTKGGVDSFDQMCKSMSANRKTNRWPMAVFYGMLNMAFVNSYIIYCHNKINKQKKPINRKEFMKNLSTDLT
>Har_ABS18391
GPTRMCRNIVDPLLCFQLFITDEIIHEIVKWTNVEIIVKRQNLKDISASYRDTNTMEIWALVGILTLTAVMKDNHLSTDELFDATFSGTRYVSVMSRERFEFLIRCIRMDDKTLRPTLRSDDAFLPVRKIWEIFINQCRQNHVPGSNLTVDEQLLGFRGRCPFRMYIPNKPDKYGIKFPMMCAAATKYMIDAIPYLGKSTKTNGLPLGEFYVKDLTKTVHGTNRNITCDNWFTSIPLAKNMLQAPYNLTIVGTIRSNKREMPEEIKNSRSRPVGSSMFCFDGPLTLVSYKPKPSKMVFLLSSCDENAVINESNGKPDMILFYNQTKGGVDSFDQMCKSMSANRKTNRWPMAVFYGMLNMAFVNSYIIYCHNKINKQEKPISRKEFMKKLSIQLT
>Hma_XP_004211847_PGBD1 ?
GPTRFAAQVCGQRLDTAFKLFITPEIRIIVNCTNAEARRIRLEGWVDTTVNELFEFIGVLLLAGVFHSKNQNIKEFWSKLDGIPIFSISMQRDRFVNLQRCIRFDEREIRNQRRFEDKFAPLRNIMEMFITKCKSNCNPSAYLTVDEQLVTFRGRCPFKMFIPTKPGKNGMKIWILCDSETSYCINLQPYIGRVNGERDVGQGTRVILELADHLNGSGRHITADKFFSNIHLARALLGRKMTYTGTIKKNKEEIPKKLLPALHRPVYSSIFGFQIDSTIVSYVPKKNKAVILLSTIHHSNDIVMDHNEKPCIILDYKKYKGGIDTLDRVVRCYSSRRKNNSLMYGIGFR
>Tru_Pigibaku1_XP_011603527.1
GPTRMAVTHTQDIKSSFELFIPDSIQEIILDCTNLEGRRVFGERWKELDQTQLHAYFGVLILAGVFRSKGESAESLWDAETGREIFRATMSLENFHIISRIMRFDNRDDRPARRQRDKLGAIRTVWDKWVRRLPLLYNPGPNVTIDGQLMPFRGHCPFRQYIPSKPAKYGIRIWAACDATSSYAWNLQVSTGKPDGGAPEKNQGMRVVLDMSQGLSGHNITCDSFFTSHKLGQELLKRKLTIVGTIRKNRSELPPQLLTSKNRPVKSSKFAYTADTSLVSYVPKKGKNVVLMSTLHRDGRMCDQEHHKPEIIMDYNATKGGVDNMDKLVTAYSCKWRTLRWPLVIFFDMLDISAYNAFVIWMALNPEWKGVKLQKRRLFLEDLGKELV
>Oni_XP_005458919_PGBD4 ?
GPTRYATSRIVDPISSFALLLTDEIVQHIVSMTNLHGKRKIPGWRDIDAEEFRAYVGLLVLSGVYRSKHESTISLWSEKWGRSIFRATMSQKRFQHISRALRFDDKLSRPPRRVDKLAPFRKVWNMWTHRLEMLFSPDRDLCVDEQLVSFKGRCSFRQYMPKKPAKYGIKIWAACDVKTSYAWRLQVYTGKPAPDRVEVNQGMRVVLDMTEGLQGHVVTCDNFFTSCALADELLKRKMALVGTIRQNKPELPPHLLQAKKRALFSSIFAFTKTRTLVSYTARRGKNVLLLSTKHRRPDVSTEGKRKPVIIQDYNKCKGGVDKLDQVVGTYSCRRRTNYWPLALFHNLLDVSLYNAYVLWTSIEPSWQKQKGYKRRLFIEEVGEMLV
>Pny_XP_005755147_PGBD4 ?
GPTHYAVARISDPLSNFRLFLTDEIMHHIVEMTNLHGRRTISDWRDLDNDELLAYVGLLILAGVYRSKHEATTSLWSEKTGRSIFRAAMSQKRFSHITSALRFDDKLSRPRRHTDKLAPFHEVWDMWTHRLMMLFSPDRDLTVDEQLVPFKARCSFRQYIPKKPAKYGIKVWATCDAKTSYAWRLHVYTGRAAGERAEVNQGMRVVLQMTEGLQGHVATCDNFFTSFALAEELLKRQIALVGTIRQNEPEISPVLRQTKGRAIYSSTFAFTVRHTLVSYIPRWGWNVLLLSTKHRRPEVSDGEKRKPVIIDDYNQCKGGVDNLDKVVGTYSCRRRTNRWPMAVFHNMIDVSLYNAFVLWTSVDRSWLQQTPHRRWLYIEEVGESMI
>Mlu_Mitra PNAS 2013_PiggyBat
GLNTDAVINNIEDAVKLFIGDDFFEFLVEESNRYYNQNRNNFKLSKKSLKWKDITPQEMKKFLGLIVLMGQVRKDRRDDYWTTEPWTETPYFGKTMTRDRFRQIWKAWHFNNNADIVNESDRLCKVRPVLDYFVPKFINIYKPHQQLSLDEGIVPWRGRLFFRVYNAGKIVKYGILVRLLCESDTGYICNMEIYCGEGKRLLETIQTVVSPYTDSWYHIYMDNYYNSVANCEALMKNKFRICGTIRKNRGIPKDFQTISLKKGETKFIRKNDILLQVWQSKKPVYLISSIHSAEMEESQNIDRTSKKKIVKPNALIDYNKHMKGVDRADQYLSYYSILRRTVKWTKRLAMYMINCALFNSYAVYKSVRQRKMGFKMFLKQTAIHWLT
>PLE-wu_ref ?
TPTRVLPSNARPIRYFEKFFTQEVFELIITETNRYACQNNVIGWTILDIKELKAFLGILIIMGYNILPTFELYWSSDPEFRVDEIASTMTFRRFKQILRCLHLNDNSKQPARLSPEYDKLFKIRPLLTLINTSFQENAHNSSSQSIDESMILFKGRSTLKQYMPMKPIKRGFKVWCRCDSITGYLYEFDIYTGRDGDRVEDNLGGKVVKKLTEKLKGMAAVHVTFDNFFCSYDIMNYLHVNGISASGTVRRQRADLPKLVKSKKKLKLTKGQYKWRVKENVAFVIWQDTKEVLFMTNAFHPKDNETSLPRKGRDGSKTDVRCPAVVKEYTKRMGGVDHFDHIKGTYSVGRRSKRWWLRIFYFIFDACITNSFLLQGKNANATKLSNLEYRVALARGLI
>Aca_XP_005091401_PGBD4 ?
GPLHNLDENSKPIDFFHLFVPPTFLDEIVVQTNLYAEQCQNNKGKRDTYWKAVTVSDIRKFLYLNIMFGIHHVPDSRLYWSVDPVLRVPAVADVMSRQRFEKINQYFHLNDSTKMPNRGEENYDPLFKLRPLLDTVRTACGSSYKPGRNISIDEAMIGFNGRLHFKQYIRNKPTKWGIKVWCVAEAETGYMLNFRFYTGKINEPMTDGVGHHVVMNCAADYLGKYHCIYFDNYFSSVRLAEDLLKKKTYSCATVRTNRKGWPFPTKNKQKKGTLNMKQRGTMVATQWHDKRPVNVLSTCCNPTTTDVTRRTKEGVVPQTIPTPVHDYNQNMSGVDLADQYRSYYNIGRPGKKWWRYGVWFLIQTAIINAFLLMRRANPNARRRSPGADHLHFRIALLQDLL
>Ami_ACT79641
SPVGITFEIGNEARELDVLKKLFNDEILNVIVRETNRHARQKLAGDALDKWQDVTLEEIKAFLGVSVVMGVNILPSISDYWSSNQFLGNEGIQKVMTKNRYENISRFFHFNDSSVEPRRGEDGYDRLYKVRPILSHFNAKIQEIYKPGKNISVDEGMIGFKGRLSFRQYMPAKPTKYGIKVWMAADASNGFVINHEVYLGKQRGRVLANGLGYSVVMELMNPFLNKNHHVYFDNFFSSPKLLEDLQNEGTYACSTVRAGRVGLPPSSRRKLKREGEMICEQKGNLVYTKWHDKRDVNILSTNFDPLEPKTVKERWKKNGDVVLVEKPACVDLYNTSMGGVDRTDQLRSYYSACRPSKKWYKYLFWFIFDVSLVNSFIIFKENVDRRGRRTLVNFRLALATQLI
>Nap_EQB62075_PGBD4 ?
GVEKWQAVKEITSVINEYLEEEKFLYDTDCNDPFALYKLFFTDYILEMIVEETNKYATQGIKNSSSSSRIHQKAWQSVTKDEVNTFIGILLIMGVVQLPEIRLYWSNKDMYANARIKKAMKRDRFLSILKFLHFADNTTARTEDRLYKIRNIFETIVDSFKSSIRPGKDIVIDESMVPWRGRLRFRQYIPGKRHKYGVKLYKLCLPDGYTYNIEIYAGKNNTIIKKSHSHDVVMRLLNGLLFEGRILFTDGYYTSVPLGEELLQNNTFICGTVKINKKFLPPQAKQKQKRGELMHFENRSGVKFLKWTDKRPVCMLTTSKNHRCKFVTGTNGKVKPDAVFDYNIAKKGVDLSDQLSGYYSCLRKTIKWYRKVVIQLICGTSLVNAWYIHKRWGTKNMNLLQFREVIIDRLL
>Goc_XP_003740690_PGBD4 ?
PQSSYLGDPQNLRPAQVYRDFFDKDLVRMIVAETNRNAKNYIDANTIGRSSRAQRWEDTSSDEIEKFFGIVLYMGLVPYPSIPDYWSTNDLYGNRIVATTMSRNRFQLLLRFVHFSNNQKENASGRSSKFIRVLEALQDKFCLAYTPGDMLVVDETMVPFRGRLSFRQYIPGKSHKYGIKIFKLCNKDGYTFRTKVYTGAEGVRSRALPSDIVIELSEPFLDDGWTLVTDNYFTSVPLAKELLGRHTNLLGTLRRHRKFLPQNVVTARLDRNQLTGRISRDGIVVGKWKDKRDVLFLSTKHDLRMASSGRRVAKKDGVQKVKPLAVIEYNKAKQGVDISDQLASFYSPLRKTIRWYHKVVFELLLNTAVVNSRIIFNKLTGRAMSMKTFRAAIVEDFL
>Mro_XP_003708235_PGBD4 ?
GAKTTNEKVRICTKAGEFYALFVTDEIFQHISEQTNHYATQCRIISKRTTNWTPTNKNELKRLFGLLIWMGMVNLPSLRLYWSQDPLFSQTFPRTIMSRDRFEILMRMVHFADNEAAVANNRLSKIQFIIDELNTNFQKYYDPPELLCIDESLIPFRGRIVFRQYLKQKKHKYGIKIFKLCCSHGYTYNCHVYTGKALDRENTTPSNVVMSLLKNLFHRGHTLCTDNWYTSIDLANRLIEKNTHLIGTLRTNRRGNPSEVIKTNLKRGQVIAKENNQGITVLKWKDKRDVLVLSTKHSNEMINVKTKRGFCCKPKIIVEYNKAKTSIDLSDQMSAYSSPLRRTLKWYKKLAFELCLNTAVVNSLFVFQEVTGQKISITEFRRQLVNELT
>Xbo_BAF82021_Uribo
GPPCNYAPEIPPFTAVSGVKVDTTNFEIMDFFNLFITEAILQDMVHFTNLYAEQYLASHSLPVYSRAQAWYPTNVNEIKKFLALTLAMGLVELNTIASYWDTTTVISVPLFSAVMPRNRYQILLRFLHFNDNAAAVPPNEPGHDRLYKLRPLIDSLSQRFAEVYTPSQNICVDESLLLFKGRLKFRQYIPSKRSRYGMKFYKLCESSTGYTRSFMFYEGKDTNLDPPGCPLDLTASGKIVWELITPLLGQGYHLYVDNFYTSVPLFRTLYWLDTPACGTVRQNRKGLPKELVQKKLKRGEVHALRSDELLALKFADTKPVCMLTTIHNESVVVQHRRGRPAKSKPLCCKEYSKHMGGVDKTDQIQTYYDATRKTRAWYKKAAIYMIQMALYNSYVVYKAAVPGPKLSLYNYLLQLLPALL
>Mfa_EHH62949_PGBD4 ?
TPGRKVDVSDITDPLQYFELFFTEELVSKITRETNAQAALLASKPPGPKGFSRMDKWKDTDNDELKVFFAVMLLQGIVQKPELEMFWSTRPLLDTPYLRQIMTGERFLLLFRCLHFVNNSSISAGQSKAHISLQKIKPVFDFLVNKFSTVYTPNRNIAVDESLMLFKGPLAMKQYIPTKRVRFGLKLYVLCESQSGYVWNALVHTGPGMNLKDSADGLKSSRIVLTLVNDLLGQGYCVFLDNFNISPMLFRELHQNRTDAVGTARLNRKQIPNDLKKRIAKGTTVARFCGELMALKWCDEKEVTMLSTFHNDTMIEVNNRNGKKTKKPRVIVDYNENMGAVDSADQMLTSYPSERKRHKVWYKKFFHHLLHITVLNSYILFKKDNPEHTMSHINFRLALIERML
>Ggo_XP_004055963_PGBD4 ?
TPGRKVDVSDITDPLQYFELFFTEELVSKITRETNAQAALLASKPPGPKGFSRMDKWKDTDNDELKVFFAVMLLQGIVQKPELEMFWSTRPLLDTPYLRQIMTGERFLLLFRCLHFVNNSSISAGQSKAQISLQKIKPVFDFLVNKFSTVYTPNRNIAVDESLMLFKGPLAMKQYLPTKRVRFGLKLYVLCESQSGYVWNALVHTGPGMNLKDSADGLKSSRIVLTLVNDLLGQGYCVFLDNFNISPMLFRELHQNRTDAVGTARLNRKQIPNDLKKRIAKGTTVARFCGELMALKWCDGKEVTMLSTFHNDTVIEVNNRNGKKTKRPRVIVDYNENMGAVDSADQMLTSYPSERKRHKVWYKKFFHHLLHITVLNSYILFKKDNPEHTMSHINFRLALIERML
>Pgbd4-Hs_NP_689808.2
TPGRKVDVSDITDPLQYFELFFTEELVSKITRETNAQAALLASKPPGPKGFSRMDKWKDTDNDELKVFFAVMLLQGIVQKPELEMFWSTRPLLDTPYLRQIMTGERFLLLFRCLHFVNNSSISAGQSKAQISLQKIKPVFDFLVNKFSTVYTPNRNIAVDESLMLFKGPLAMKQYLPTKRVRFGLKLYVLCESQSGYVWNALVHTGPGMNLKDSADGLKSSRIVLTLVNDLLGQGYCVFLDNFNISPMLFRELHQNRTDAVGTARLNRKQIPNDLKKRIAKGTTVARFCGELMALKWCDGKEVTMLSTFHNDTVIEVNNRNGKKTKRPRVIVDYNENMGAVDSADQMLTSYPSERKRHKVWYKKFFHHLLHITVLNSYILFKKDNPEHTMSHINFRLALIERML
>Sbo_XP_003935808_PGBD4 ?
TPGRKVDVSDITDPLQYFELFFTEELVSKITRETNAQAALLASKPPGPKGFSRMDKWKDTDNDELKVFFAVMLLQGIVQKPELEMFWSTRPLLDTPYLRQIMTGERFLLLFRCLHFVNNSSISAGQSKAQISLQKIKPVFDFLVNKFSTVYTPNRNIAVDESLMLFKGPLAIKQYIPTKRVRFGLKLYVLCESQSGYVWNALVHTGPGMNLKDSADGLKSSRIVLTLVNDLLGQGYCVFLDNFNISPMLFRELHQNRTDAVGTARLNRKQIPNDLKKRIAKGTTVARFCGELMALKWCDTKEVTMLSTFHSDTVIEVNNRNGKKTKKPRVIVDYNENMGAVDSADQMLTSYPSERKRHKVWYKKFFHHLLHITVLNSYILFKKDNPEYTMSHINFRLTLIERLL
