# Supplementary material for: Six domesticated PiggyBac transposases together carry out programmed DNA elimination in Paramecium
Source: eLife. 2018 Sep 18;7:e37927. doi: 10.7554/eLife.37927 (PMC6143343; doi:10.7554/eLife.37927)
Supplement: Supplementary file 5. [file elife-37927-supp5.rtf]

Supplementary file 5. Sequence of the synthetic PGML genes used for protein production in insect cells (fasta file)

All sequences are displayed from the ATG initiating codon to the TGA stop codon

>Synthetic PGML1
ATGTATCCCTATGATGTTCCGGACTATGCAGGATCCATGGAACTGGAGAAACCGATTAAACAGGATGTCCACAACTATCCTCCTAAAATTGAGCAGGCTCAGGAACAGCAAACTGTTCAGATTGAATTCAAGCAGATCCTGCAGGGGCTGGAAGATCAGATTGCGCAGGCGTTCCAAAACAGCGCCTATTTTGAGAAAGAAAACTTTAAAGACCACCATTCAATCCAGATGGTGATCTTTAAAGAATTGCGTGCTACCGAACAGTGGCAACAGCTGCAGAATGAAACTCCGATGGGCATTTATAATTCCATCTTCCCGAAAAAGTTGATTCGCTATCTGATTGACCAGATCAACGTCAAAATGAAAACGAGCTTTGCAAATCAGGATGAGAAACAGATTCGTAAGAAACTCGTGAAGATCGATCAGATTTTCGACTTCTTTGGGATCAAAATCATTATGGGCTATATCAAAATGCCCCATCTGGAAGATTACTTTAACGAAAAGCAGCTGTTTCAGTGCAAAATCTTCGAGTGCTTTAAAGAAGGTCGCTTTAAATGCTTAGAACAGTGGTCTAACTTTACCGATGAATGTCTGCTGAAGAACATTAATAAAGCCAAGCCGATCAACGAATTCCAAACAATTCGTGTGTGCAAGAAAATTGGCAAAACATTACGCAACATCAAAACTCCAGGTAAAGACCTTATTCTGCAAGTTAACTCGGGCTTTATCATTGAACAATCTATCAACTCCCAGCAGATTATGATCTGTGATCCGAAAAGTAAACTGATTGTGTGGCAATATTTCTGCAACAATATTCAGGAAGAGAAAGTAGGGCTGCAGATTGTCCATGTGCTTCAGCAGTTTAAAGATAACAACCATACGCTTTACATCTACGATAACATTCTCAACTTAGAGCAAATCGCGTATCTGAAGATTAAACTCAAAATTCAATGTGTGCAGAAACTGGACAGCATTCAAGCGAGTCAGGTTCCGCAAATTCCGCTGTATCTGGATTACATGAGCATTATCAAAGAGCAAAATGAAGTGTTCGCCGTAACATCGGGTGAGCAGATTCAACAGCTCATTTGTACATACAATCAGTATAAAGAAACCATGCTTTTGTACTGCATCCACAAATTTCGGCCGAATTTTAGCTACATTCACTACAGCTCGGCGATCATGACGGAACTGCAGGAAATTACCATTTGGAATTCTTACCAGATTGTGATGAAGTTGAAAGCATCACAACATCAGCAAACGTATGAACAGTTCCGCTTAGGACTGGCCCAAGAATTACTGAAGAATAAGATCACCCAGATCAAAAACGTTCCGACCTTACTGCACAAACAGAAAGTGGTCAATGTAGCACTTGGCACCGAGATTTTCGGCGACTACGGTCAAGGCACGAAAATTAACACCAAAAGCTTATTCCATATCCCAACTATCATGGCTACCCCTAAGACCTATGCGGGCTCAATGAATTGCCTCGTATGTAAACGCAACACGCAGCTGATCACGCTGTGCAAACCATGTTCCGAAATCTCTGGTAAACTGGTTATTCTGTGTGCGTGTGATTGCTTTTATCTGTTTCACCAAAATACCTTGGAGTACATCATTAATGGTGAAGATGTTACTCAGACCCAATACAAAAGCATTGAGCAGAGTTCGCAACAGTTGTACCATCAGGCCTTTCAACAGAAACCAGGTCTGATGTATGAAAATGTGGACAAGGGCAAAAGTGTCCAATATGCCAAACAAGTCGAAATTGAATGA
>Synthetic PGML2
ATGGATATCGAGCAAGAAAACGACCGCGAAGTGGCGGAAGATCAGAATCCGCAGGAACAGATCAATAATAACAATAACAACGAAGAAGAACAGGCTGTTGTGGAAGTGAATAATCAGAAGAATCAAATCTCCCAGTCCGTCATCGACTGTGAAGTGCAACTTCCGGCAGCGCCTATCAAACTCGATAAACAGGAGCCACTGAAACATGAGCATGCCCCGGGCCAGACCATTCGCTCTGAACAGTTACGCTTACAAGTGGGGAAGAAATTGGCCGAAAACCCTGATTTCTACAGCAAAAGTAAATGGCAGTCTATCAATGCTTCCTCAACCACGACCAAACAGCTGCCGTTTCAGGGTGACCGCAATGCATATAACTTTTGGAAAAGCCAGGTAACCAACGACGCAAAAGATATCTACTACTCACTGTTTCATCGTCAGATTGTGCAGTCGATTCTGAAAATGATTAATGACCAGATGAAAGATCACTTTAGCGAACAACATGAGAAAGACGTCAAGAAACGCCTGTTCAAATTAGACCAAATTTATGAGTATTTTGGGGTGAAAATCTTGATGGGCTATAACCGCATGCCTGATCCCGAAGATTACTTTAACGATAAACTTCCGTTTCGCAACAAAATTGGCGAACTCATTAAGGTAGGCCGCTTCAAATTTCTGGAACAGAACACCAATTTGCGTGACGAAGTTATGATCCAAGCGAAATACCACAACCTCAAACAAGAAGATGTGGAAAAGAAGTTCAAAGTTGAAGGCGAGGTCTATTCATACCTGACTAAAAAACTGAACAAGAAATTCCGTATTAGTCATGATGCCGGGCAATGTCTGGCCGTCATTAAGAACAACCTGTTTGAGGCTCAGGATCTCCAAGGTCGTTCGATTGAGTGCATCTTACTGATGGATGTGCAAACACAGTATATTATCGCCATTCGGTTCTGCTTTCGCGAAAATGTGTCGAATACGATTTTCAATATGCTGGACCCGTATAAACACAAAAACCACAAACTGTATTTCCAGCAGGAGCTGCTGACCCTGGAACAAGTACAGATTCTGGTCGAGTTCTTTGCGATCTACAGCTGTGGTATTCTGGCCTCTACAACCCAAACGCAGAAAATTGACTTTAAAGATGGACTGGTTACCAACAACCATGTTATGCTCCTGAAATTACTGACGCCCAATCAAGCTGACTTCAAAGTGTTCGCTTCTACGGCAGATGGATTTCACCGGAACACCTCACAGGACAAGAAATCCTATGCAGTGGTAATTTATCAGGAGTATATCAAACTTGTTGAACAGTATCCGGCACTGAGCGATGCCTACAAATGCCTGTTTAGCTCTGCCACGGTTAACGGTGCGATCTACACTGAGATGCAGGAAATTGTCATTTGGAACTCGTTTATCGCGTTCAAGAGCATTCAGAAGGTGAATTCGCAAATCAACTTTCTGGAATTCCGTTTAACGCTGGCCAAGCAACTGCTGCGCACCAAAATGAAGAATATCCCGAATGTGAGCGAAACTCACCATAACCTTCAGACTGCGCAGCCGCGTTATAGCACAATCGGTATTGTTACCGATATTATGGGTTCATTTAACCCACAGGATATGGAGCTTTTGCCGAAAATTAACTTCCATGTGCCACGTAAATGTAATCCTCCGGCGAAGTTGCAGAACAAATTAGTCTGCTTGGTTTGCAAAAAAGTTCCCACGGAAATGGTAGAATGCGAATCTTGCTCGGAAATTAGTGGCAAACTCATTACCCTTTGTGCAACTGAATGTTTCAGTTTGTTTCACCAAGAACCGAAAAAATACGTCCAAAGTGCGATGGATCTGCCAGTACTGCAGCAGTCCGCGCAGTTTGAGCAAAGCACACTGGGCAATAGCGAAGCGTATCAACAGTCCTACATGAATCGCCAGAGTAAATGA
>Synthetic PGML3a
ATGTATCCATATGATGTACCGGATTATGCAGGATCCATGAAACAGGAAACCCTGTCCAACAGCACCACCCAAACAATTGGTTCTTCACAGCAGACGCAGTCACTGGACGAAATCGAAAAATACAACTGCTTTTGGAAGCAAGAAATTATTTTGAGCGATTGCGAAGAACAGTTCGTACAGATTGTCTCGGACGACGAACGGGACTGTGCTGCGATGGTATCGTTCGGTAAAGCCCTGAATATCCAGCCGACCGATATTAGCCAGATCACGGAAATTCTGAATTACCTGGACACTCCGCAACTGAAAATGACCAGTCTGCAAAGCCCACAGAATGTCCTGAACTACTTCTTACCCAACTCGTTTGTGTCGACGCTGACTGAGATGATCAATCACTATCTGCTGATGGTTTTACAGGAAGATGTAAAACAGCAGCAACAAACGCAACAGATTTATCACAAGAAGAAATACAAAGAAGCCGAAATTCAGTTCTACCTTGGCCTGCAAATCTTATTTGGCGTGTATCGCTTCCCGAGCCTGGATGATTATTGGAATGCGGAATCTTGGTTGAAAGGCGGCGTGGAAGTCACTATGCCGATTGGGCGCTTTAAATTTGTCGATTTACACATCTTCTCGTATTTCGATGAGCAGCAACGCGCCAAACTGCAACTTGAAGTGTCGAAATTCAGCAAAAAACTGAAATCCCTGTACAACCCGGATCAGGAATTGATTATCGTTGAGCAGAACCAGAAAGCGTATAAAGCCTACTATATCTTCGACTATGACAGTTCACAAATCATTGATCTGCTCGTTGTGTGTAACAACGTGAAGAATGAGGACCGTATCAATCGCGTGATGCGTATGCTCTATAAATACTCTAATCAGAACCACGTGACCTATATTCTGTTTGATTTGAGCTTAGACAAAATCATTCAGCTGGTCGATCAATCGATTTATCCCGTTATTCGCTATCAAAACATTAGCCACAACCTGATTCAGATGCTGCAAGATGGGGAGTACGAACTGGACCAACTGTTTCTGGTGAAGCAAGGTACCCAAGTGGATGTTTTCCCGCAACGTCGCTTAATCAGCAATCGTCCTTATATGACTAGTTACGAAAAGATTAAGGAACAGTTACAGGAGCATATCACCAATTTACAGCAATTCCGCAGCAACTTTTATCAAAGTTCCCAGTTGAATAATACGGAAGGCTTTCATCAGTTGCGCGTTGAATTTGAAGAATACTTTGAGATTATGATCCATAACACGTTCCTGCTGATTCAGCAGACATCACAGAGTCAGTTTCGCCATGATCTTGCAAAGATCCTTCTGAACGAGAAAGGTCAGCAGATCGAGCGTGATCGTAATGAAGCGAAACGCAAACTTAACGTGACCTATCAGGAGAACACCTCAAACACCGATCAGCTCTCTTTTGGCCTTCAGAAACAATATGCTGATCAGTTTCATACGCCAATTCCGCAAAAGCAGGAGGATACCTTTAAATACTGCCTGGTGTGTATGAAATTCGAAGGCCTCACAAAACCGTATTACCGCTGTCAGCTGTGCGAGAAATTACTGAAAGTCAACAAAATTTTCTTGTGTCCGTTTCCCTGCTTTGAACTGTTTCATCGGAACCCGAGTGACTTTATGGTTTGCGACATGAAAATGCTGGAACCACTTGGAGAAGGTGCGTACTTCAAGAATGAATCCACGATGCAGCTCGTTTCCAATGGTCAGGAAGAGGATGCATCTGGGAAAAAACGCAAATACAACAAACCTCCACCGCGTACCTTTTGGTCCTTCAATAAGGAAATCTACAAACAGGCAAACATCAACGATGATCAGAATCTGAATGATATTCTCAACACCTTCAAGAAACCTCAGGTACCGGCGCCTAGTCCCAATTTGCTGCAACCGATTGTGCCTCAAGAGCAGCAAGTCCAAACATCTACTCTCGGTCACAAGAAAGACATGAAAGACATCGACCATCTGCTTAACCAGGAACGCGAACAGAAAGACAAACTGATTAAAGAGATGCAAGAACGTGATAAGCAGCTGCAGGAACAGTATTTTCAGCAACAGTATGAGAAACAATTGCAGAAAGAACAGGAGTCTAAAAAAGGCAAAAAAGCTCAGAAGGAAAGCGAGAAAAAAAAACAACTGGAAGAACAGCTGAAGAATGTAAGCCCGCTCGAAAAATTTATGGAACAGATTAACAAACGTGCCAAAGTTGAGGATGTCGAATGA
>Synthetic PGML4a
ATGTTTAATGAAGAGCTGGGAGTCATCGACTATGAGGTCGAATCCGATGACAGCATTGAAATGATTGATAATCCCGAAGATATTGTGATCCCGACTCCTATTCAGGAACAGAAACAGATCACCCAACAAATCATTGAGCAACAGCCGCAACAGCAGCTTAATATTGAAAATACCGAAACTATCGAACGCCAACAATTGGAAGAAGAGCAGATGCTGCGTCGTCCATATGCGCTCCGTTGGAAAAAAAAATCCATCAAAAAACTGGAGAAAATCACCTCTAAGCTGACAGATCAGGACTATGACGAAATCAAAGACGTTCTGACCAAGAAAATCGCCATGAGCGAATCTAAAATTAACAACTCTAATTACTACAATAAGAACAATACCACCACCAATAACAATACGAATAACGACGTCATCATTAGCGGCAGTATCCTCAAAAACCCGCAGACGACTTTGAACCAAGTGCAGAAACAGAAACAGTTTCAGGTGTTAGTGGAGCATTTCGCCAACAAACCGGATTTTCTGGAAGCGATCATTGCGCAGGATCTTGAACAAGAGAACGAAAATCATGACGGCGGCATTCTGAAAAAACCCATGATTGTTCCGCCAAAAGCAGGCCTGAAGAAGCAGCAGGGTCGCAAAAAAGTCAATGTTACATTTGAAGATCAGTCGCAACCGCCTAACGGCGGGATTCAGTTTATTAAAACCAGCGTTCTGCCGCAGAAACAGCCGCCACCGCCACAACCTCAGCAACAGATTCAGAATATTTTATCAAAATTCCAGGAAATCCGCGAGAAAAGTGCAGCCCCACCGCAAACGCAATATACGACAGCGGTACAAGAGTCCAGTAACATGCCGTTAGGCACCGGTGCATGGTATCCGAATAAGCAAGACCGCAAGAGCCAGCCCTTTGTACCTCCGCACGCAGGAAGTAACCCGAAAATGTTACCCACCGTGTCGTCTGTGCAAATGATTTCGAGCTCTCAGGCCGCACAGCAGTCTCTGAACCACAAGTTTCTCGAAATGAATCAGAATCAGAGCCGCATTAGTAACTCTCCGAGCCGTATTCGTGATATGATCACGCTTCAGTATACGAGCTCATACCAAGTGGACCGTCCTCCGATCATGCCGAGTCGTCAGCAACCGGTCTTTCAGCAACAGACGTTGGAGCGCAATATCAACTACGAGCTGAACCAGGCCAAAAAAAACAAAGAACAGTTGTCCGGCCACCAACAATCTCAGTTGCGCGAAAGCCAAATTAAGCTTCAGGCGAAAATTCAGGAATCTGTGAAACGCCAGAGCGTGGTGCAGCCTACGATTGAGGAAAACGACTCCGATTTTATGGATGATGGTATGGATTCCCAACCGTTTATCGATTTTAAGCGGGCGGGTAACATCCCTTGCGCCAGCCAACAACAGACGTATCAGCAGTATTGGCAAGAAATTAATTTCAATACCGACATTCCAAAAGATTATTACATGAACGAGAAATTCCGCGTTGATCTGGAACAGGAAATTGAAACATCCGATCCATACGAGCTGTTCAAGCTGTATTTCGACCAGTCTATTTTCAAATATATCTGCCTGATTTCCAACAAACGTCAGTGCATCCGCATTGACGAGGATATCCTGGAATCGTTCATTTCAGCGCTGATTTATATCTTCTACATTCAGCTGCTGGGTATGCGCGAAATCAAACGCGTCCGTTTTGACCATATTCTGGATTACGTAACGTTTGGCCATATTTGCAAAGAAATCCGCATTGATGAGCTCGAGGATTACAGCTTTATCTTTGAGAAAATCAGTAAGAACTTCAAGTCACATTATCAGCCAGAGGAATTCCTGACTCTGGATAGCCCCATTTTCTATCAAAATCACTCGGTTGATGGCTTGATTTCGTTATCTGACGGTATGAAAGGCTACGTACTGGACTTCATTTATGGAATCAAGGAACAGAAGATTCTGCAATCTCTGCAGATTTATCAAGGTAAACACCATAAACTGTACCTGGGTCCGGATGTTTCTTCGCTGAATCTGATCATCCAGCTGAAGAAGAAACAGTTTGGAGCTCTGGCTAAAGTGGTAGATAAGCAGACTCAGCTGACCCAAGACCAAATTCAAGAAGTTAAACAGAACGCGCAGAAAGGCCAAAGTACGCAGTTTCTCAGCAGCGATAACCAGACCATTATGTTGATTTTCGCGGAACGTCAGCAACACCTTCAGCAAGTCGAAGGGTTCGTGTCGAGCTTCGCAGACTTTACCCGTTTAAAACCGCAGGATACTAAAATCAAAAGCGATGTCAACAAACCGATTATTCTGTACCTGTATGACAAAATCAAGACGCAGTATGATAAACGGGGTAAAACATACCAATACTCACAAATCCCGCATCAGAACGGCAACCAGCATCTGGAAATTTTGGTCCAGCTGGTATATTCAAGTATTTACAATGCCAATATTCTGAACAAAATTAAGAACCAGTCGGCTTCCTTAACTCCGGAGAAAGCTAAACAGATGTATCTGGAATTTGTCAAACAGCTGTTACATTCCTTTTATGTTCGCTCGTTAAAACGGCGCGGCATCAACCAGTTCCCGCAGAACCACACCTTGGAAAGTGGTGATACAGGGACGTTTAGCTGTATTGAATGTGGGGAATCCTCGCAGACCATCTGTCGCGAATGCTCAAATCACTTTCAGATGCTCATTCCTGTGTGTCGCAGCAAAAATGAACAATGCTTACGCTCCCACATTGAAATGCTTGCATCACAGCCCAAAATTACCAACACTAATCGCAAACTCACCAACAAACAGGCGATTGTGAAATTTGAATTCTATCGTAGCCAGATTGAAGCGTCGAAAGATAGCAATGCGCAATCAGCCCTGCCGGTGATCGAAGATACCCTTCAACAACTGGATACCCTGGATCCAGATGAAGAATTATCAATCGGTATTCAGACCCTGCTTGTGTCACTGTCGGACATGATTCAGAAAATGTTTCAGCAGCCGAACGTACTCGTTCCGCAGAAAATCCAACAACATAAACAGGAGGTTTACACATTGAGTCAAATTGATCCTCCCATCAAAAGCGTTCAACAACTCCAGAAATCGATCAGTCAGGTCTACAACAATAGTCAGGAAGTGAGCCATAAATCCCAGATCGTGCCGCAAATCTATAAAACCAAAATTGGGGAACAGATGAACATCGAGCCAAGCATTCAGAAACTGCAGGCTCGGGTTGCCCGTATTAATAAGTGA
>Synthetic PGML5a
ATGTACCCGTATGATGTTCCGGATTATGCGGGATCCATGAGCGAAATCACCAGTAATCAACCGCAGTTAGAAAGCCAATTTCTGTATGAGCTGGTGAACCAGATCCCTCAACCGATCATCCAGAATCAATCCTCGCAGCATGTCTCAATTGATCCGCCTCCAATCCAGACCCATGACCAGCTGCCGCAGACCAATAATACGACCGAACCAATTGTACAGCAGCCGATTCAGGAAGATAATACCGATCCACAAGACCAGATTCAAAACCAGGCACAGCTTCAACATCCACAGCCCTCAACCAACCAAGAACCCCAGCAACAGGAGGATCAGCTGATTCCGCCTCGTCAGGAAGTTAACTCATTAGTCATCTTGGCTGCCCTGGAATCTAATGACGAGGATAAAAAGTCCCAAGAAGAAGCGGATTGCAAATTACTGTACAAGAAACAGCGCCAAACTTCGATCATTATTGATGACGATATTCAGGACGACAAAACGACCCCGAAAAAAACGGCTAAGAACGAAAAGAATAATGTGCTGTCGCCGCAGCAAGACGATCCACTGGCCAATAGTCAGAACGATTCTGAGAAACAGGAAGTAGTTCTTCCGCCGCCGAGTCAACGCTCGTCAGAAGAGATCCAGCCTGAAATTCAGAAGCCTTTTGAGAACAAAACTGCGGAAGATAAATCAAGCCTGTTAGATCAGATGTTGAAACCAGAGCCGGCGCTCAAAAAAACGCAGAAAGAATTTTTTTCTTTTACTCAGCCTGTTCACCCCACGACCGTACCTCTGACCACCCAAAAGAAAACCAAAAACCCGATTGTGATTGACAGCGATGACGAACTGCCGCCGCCAAGTAACATTAGCATTCTGAAACAGAAAATTGAGGATACAACCCCGTCCAGCTTCTTTCGTAAGAAACCGGTGGAAAACCAGACGCCGATCGAAGTTGGCGACAATGATAATTTTCAGAGCCAACAAGTTGACAAACAACAGAAAATTACCCAGCTGCTCCAGCAGCCTATTATCCCGGCACAAGTGTCGAACAAACCGCTTAATCAGCAGCAGCGTAAACACCAAGCGCTTCTGGAAGAAATCGTTCAAGAGGATGATGGCATCGTCCAAGACATTGAAAACCAAGACTGGACCTTATTTAGCCAACTGAATAACATGGTGCCACCCAAATTTAGTGGACAGTTTCGTATGCTGGAGTACAAGGACAATCCGCAGGATATGATTCGTCTGCTGTTCGGCGAAACTCGCTTTAAAGACCTTATGAAAACCTGTCAAACCTCTGAACCGGAATTTTGGCTTTATCTGGGGACCAAACTGATCATGGGGTATATGCGGCTGCCGGACATTAACGAATACTTCTACGGGGAAGATTGGATTGCGGGTGGTGGCATCAAAAGCATTATTACGCAGCAGCAGTTCGATGACATCGATCAAAAGGTCGAAATTATCCAGCCGAAAATTCAGACAGAATCCCAGTTGGCGGTGAAACTTGAATTCAATGCCCAGAGCTTCTTCCGGCAGGAATTCCTCCCACAGTTCACACAGGAACTGAACGACCGTTTTAAAAAACTCATCCTGCCAGGTCAGGAGCTGTTTCTGATTTCCAATTTCTATACTATTATTTATTCGACCAAAATCCAATGGTATCAGCTGATTGACAAGGAATCGGGCATCATCCTGCAGCAATTCTTCTGGGCAACGCCGATTAACAAAGCGCTGGATCTGAACAGCCATCGGGATCTGCAGAAACGCTTGCGCATGATGTTTGAACCATATCACCTCGGACGTCATATTGTTTATAGTCAAGGCCTGCTGAATGCCGAGTCTGTTCTGCAGCTCTATCAGCAGAAAATTTTCGTGTGCACCGATCTCATGCAGCATAGCCTGTTGCCCCATCCTCCTACCGGGGATCAACAGGTAGTCTATTCGAGCAAGAACAAAGAACCGATCTACCTGTGTTATAAAAACCAGCAGTGGATCACGTGTGTGTCATTGCAATCCCTGCAGATGCGCAGTCAGTACTTGCAAGAATTAAGTCAGTTACAGTTTACCGAAGAGAAAATTGCCATCCAGCCTAGTGAGCCGGAAAATACGTCGATTAATTCTAACAGCAAAATTATCTTTATCTTTGTGATTGAGTCGATTCTGCACAACATCCGCATTTTAAAAAAGCAAGAAATCAAAACGTTTCGCACTGAACTGGCCTTGCAGCTGATTAACAAGGTCATTGATTACAAAAACCCCAAACCGTTGTTACGCAAAGATGGCTCATTGCAAGTCGATCGCTCTTGTCAGACGGACCAGACGGTGGGCATCGTGAACGATCAGCTGTACCATTGCCCAATCCATAATGGTAATGCACGCTGCCAGGTATGCCTTTCAAAATCCATTCTGTCTAAAACGACAGCGTCCTGTCTGGGTTGCAATAAAGTGCTGGGCACTAACATTTTCCTTTGCATCTATCCCTGCTTTCGCCTCTTTCACTTAAACCCAAAACTGTATCTCAAAGAAGGTATGTATTACCAGATCGTTTGTGGCTACGACCAGTATGATCAGGACCAAGAGGATGAAGAAGTGGATCAACAGTTCCTGGATGCGAATTTACGCTTTAAGCCGGATACCAGTGAATTGGACAGCATTTACGCTAAAAGCTATCAGGATTTTAAAAACATGATTCCGATTAACTACGAGGATAATTATCACAAGAAAACAACAATCGTCGTCATTCAGCGCGAGAAAGGCGGGAATCGTCGTGGTGGCGGCCGCCCCAAGGCCTCCCGCCAACAGCTGCAGTCTGCTCAAAATCAAGCCGCACAGGCAGAACAAGGTAACCAGGGTGACACCCAGGATGCTCCGAAGAAAGTGCGTAAACAAAACAAAAAGGCGTTTAACGCACCGCAGCAACAGGGTCAACCGGAGAAAGAGACTGAACAAGCTAGCAGCGCCATGTTCGATTTCTTCTCTCAGGTACGTAAACAGGCGTACAAAAAACAAACACAACCGTGA
